# Revisiting the Heider and Simmel experiment for social meaning attribution in virtual reality

**DOI:** 10.1038/s41598-024-65532-0

**Published:** 2024-07-24

**Authors:** Carlos Marañes, Diego Gutierrez, Ana Serrano

**Affiliations:** Universidad de Zaragoza, I3A, Zaragoza, Spain

**Keywords:** Human behaviour, Computer science

## Abstract

In their seminal experiment in 1944, Heider and Simmel revealed that humans have a pronounced tendency to impose narrative meaning even in the presence of simple animations of geometric shapes. Despite the shapes having no discernible features or emotions, participants attributed strong social context, meaningful interactions, and even emotions to them. This experiment, run on traditional 2D displays has since had a significant impact on fields ranging from psychology to narrative storytelling. Virtual Reality (VR), on the other hand, offers a significantly new viewing paradigm, a fundamentally different type of experience with the potential to enhance presence, engagement and immersion. In this work, we explore and analyze to what extent the findings of the original experiment by Heider and Simmel carry over into a VR setting. We replicate such experiment in both traditional 2D displays and with a head mounted display (HMD) in VR, and use both subjective (questionnaire-based) and objective (eye-tracking) metrics to record the observers’ visual behavior. We perform a thorough analysis of this data, and propose novel metrics for assessing the observers’ visual behavior. Our questionnaire-based results suggest that participants who viewed the animation through a VR headset developed *stronger* emotional connections with the geometric shapes than those who viewed it on a traditional 2D screen. Additionally, the analysis of our eye-tracking data indicates that participants who watched the animation in VR exhibited fewer shifts in gaze, suggesting greater engagement with the action. However, we did not find evidence of differences in how subjects perceived the roles of the shapes, with both groups interpreting the animation’s plot at the same level of accuracy. Our findings may have important implications for future psychological research using VR, especially regarding our understanding of social cognition and emotions.

## Introduction

**Figure 1:**
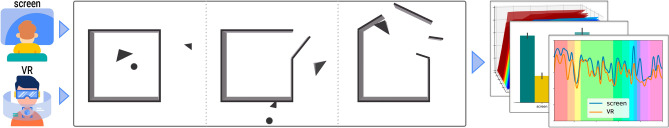
We analyze the differences in social meaning attribution when observers watch a simple animation presented on a traditional display (screen) and in virtual reality wearing a head mounted display (HMD). We follow the seminal work of Heider and Simmel^1^, where participants observing an animation made up of simple geometric shapes described it in terms of human actions and emotions. We introduce novel metrics to analyze objective and subjective data. Our results suggest that participants using a VR device develop stronger emotional connections with the shapes in the animation. These findings may have direct implications in fields like psychology or storytelling in VR.

Humans have the ability to attribute different mental states to other entities, even besides other humans. We infer their goals, desires, intentions, and even emotions by observing their actions, movement, and behavior. This ability affects the way we relate to each other, while being also a key survival trait^2,3^. Such unique ability does not only work with other live entities, but it extrapolates to inanimate objects or stylized animations as well, such as those in cartoons. The brain can attach personalities and emotions even to a set of simple shapes, building a complex, multidimensional mental construct^1,4^.

The studies of Michotte^5^ and Heider and Simmel^1^ were among the first works to study this phenomenon, and have had a significant impact in diverse fields ranging from psychology to narrative storytelling. In their experiments, participants watched a short animation consisting of three geometrical shapes (a big triangle, a small triangle, and a circle, as depicted in Fig. 1) moving in different directions and at different speeds. There was only one other figure in the frame, a rectangle with a door-like opening and a closing mechanism. After watching the animation, participants were given the following, simple instructions: *write down what happened*. Despite the actual nature of the scene, all but one described what they had seen in terms of human actions, feelings, and emotions. Moreover, such descriptions made up a connected and meaningful *story*, confirming that humans, as social beings, learn, teach, and communicate information better through stories than in abstract terms^6,7^.

The Heider and Simmel animated picture^1^ has been a recurrent subject of study in the field of the theory of mind to study the ability to attribute mental states to oneself and others, and it has influenced many follow-up works^4,8–18^. For instance, it has been employed to assess social attribution in children with autism^19–21^, and even explored in groups, where intentionality is also extended to other perceived individuals^22^.

On the other hand, VR has become an established technology that provides unique opportunities to enhance immersion, presence, and engagement of the user; in other words, “a fundamentally different type of experience”^23^. More than just a new technology, VR dramatically changes the way we perceive and interact with visual content. Many recent works have leveraged this technology for psychology-related studies in areas like enhancing social skills, social cognition, or social functioning in autistic young adults^24–26^. Other existing studies have found strong correlations between the increased feeling of presence that VR elicits and the emotions evoked by the content^27,28^. However, although VR does offer many new possibilities, it also presents new challenges when conveying engaging stories^29–34^. Given the central role of Heider and Simmel’s experiment in theorizing social agency across many subfields, it is important to examine whether the well-established findings from their experiment hold true under the novel viewing paradigm offered by VR, and to investigate what unique or different insights we may gain. Testing the foundational psychological phenomena observed in the Heider and Simmel experiment in VR is thus valuable to understand the boundaries and robustness of the cognitive processes involved, as well as our tendency to impose narrative meaning and create emotional connections even with simple shapes.

In this paper, we thus aim to explore and analyze how the findings in Heider and Simmel’s original study translate into VR, where the viewing conditions and level of engagement are vastly different and could significantly influence the attribution of mental states to other entities. By reproducing Heider and Simmel’s animation using *both* a traditional 2D display and a VR display, we allow for a systematic comparison. Therefore, the value of this work lies not only in our findings, but in systematically exploring whether and how these fundamental social cognition processes manifest in VR, as well. This exploration addresses whether foundational psychological phenomena observed in the Heider and Simmel’s experiment are consistent across different viewing conditions, which is essential for integrating VR into psychological research and applications. Specifically, we aim to address the following important questions:

- Personality attribution: How do participants attribute personalities to the elements in the scene?
- Participant congruency: Is viewing behavior similar between participants?
- Viewing behavior: Do participants’ viewing behaviors differ in VR and 2D displays?
- Viewing-Personality correlation: How does viewing behavior relate to the perception of the elements in the scene?
- Presence: Is there a difference in the feeling of presence between VR and 2D displays?

Our study does not only replicate but also expands upon Heider and Simmel’s foundational work by introducing the dimension of VR. We recreate the original animation and adapt it to be visualized both on a traditional screen and in VR. We devise a between-subjects design where one group watches the animation on a traditional screen (*screen* group) while the other uses a VR head-mounted display (*VR* group). Both groups have their gaze recorded using an eye-tracker. We follow a similar procedure as previous works^1,20^: Participants view the animation twice, the first time without any context or specific instructions, while the second time they are encouraged to interpret the animation imagining that the shapes are acting as humans. In both cases, they are asked to describe what they saw and answer additional questions about the animation. To analyze the results, we use established metrics for social attribution tasks and attributed personalities, while introducing additional novel metrics to understand the participants’ visual and attentional behavior. We report qualitative and quantitative results, including statistical analyses and in-depth discussions of our findings. Our results suggest that while many fundamental aspects of Heider and Simmel’s seminal study remain consistent in VR, important differences also emerge. Specifically, VR not only maintains the core findings observed in traditional 2D displays but also enhances engagement. Participants who viewed the animation through a VR headset developed *stronger* emotional connections with the geometric shapes compared to those who viewed it on a traditional 2D screen, despite following similar visual patterns. This highlights VR’s unique potential to deepen emotional engagement while reinforcing the robustness of the original findings across different mediums.

## Results

Our collected data includes subjective answers (Social Attribution Task^20^, Greenberg-Strickland ratings^15^) and objective metrics (eye-tracking data). Our study follows a between-subjects design with two groups, the *screen* group and the *VR* group, who visualize the stimulus on a traditional screen or in VR respectively. Note that some metrics (e.g., eye-tracking metrics) can be computed twice, since participants watch the animation twice. This is what we term *first impression* and *second impression*, respectively. For simplicity, in the following analyses, we refer as *screen* $1{\text {st}}$ and *screen* $2{\text {nd}}$ as the group of participants that watched the animation on a traditional screen in the first and second impression and as *VR* $1{\text {st}}$ and *VR* $2{\text {nd}}$ the group of participants that watched the animation through a VR device the first and second impression, respectively. The planned analyses include evaluating participants’ responses using the Social Attribution Task (SAT) and Greenberg-Strickland ratings, assessing inter-observer congruence (IOC) through eye-tracking data, examining visual and attentional behaviors, and assessing the feeling of presence using the Igroup Presence Questionnaire. Post-hoc analyses explore additional insights, such as detailed scene-wise analysis of gaze shifts and extended Spearman’s Rank-Order Correlation analyses.

For performing the statistical analyses, we consider two different non-parametric statistical tests, since Shapiro–Wilk test shows evidence of non-normality for some of the metrics (please refer to Sec. S6.1 in the supplementary). The comparison between the *VR* and *screen* groups in the same impression (*VR* $1{\text {st}}$ vs. *screen* $1{\text {st}}$ and *VR* $2{\text {nd}}$ vs. *screen* $2{\text {nd}}$), follows a between-subjects design (two independent groups), therefore we perform a Mann-Whitney U-Test. On the other hand, when analyzing how much a metric has varied across the first and second impressions (*VR* $1{\text {st}}$ vs. *VR* $2{\text {nd}}$ and *screen* $1{\text {st}}$ vs. *screen* $2{\text {nd}}$), we perform a Wilcoxon Signed-Rank test that accounts for repeated measures. To mitigate the risk of false positives, we employ multiple comparisons correction using the Benjamini-Hochberg false discovery rate method^35^. Additionally, if applicable, we investigate the interaction between time (first impression, second impression) and modality (*screen*, *VR*) computing first the differences of the metrics between the impressions and then performing a Mann-Whitney U-Test contrasting these new two variables. We perform statistical analyses to compare our conditions, setting a significance level of $p=0.05$.

### Personality attribution: How do participants attribute personalities to the elements in the scene?

In their original experiment Heider and Simmel used a visually intuitive approach to analyze participants’ answers and group-specific keywords. However, this approach lacked specific rules to compare different interpretations in a quantitative manner. To address this limitation, we compute quantitative indicators that provide useful information in line with the goals of the study. In particular, in this section we analyze participants’ answers to the Social Attribution Task^20^ and the Greenberg-Strickland ratings^15^ (please, refer to the Methods section for details about the procedure).

#### Social attribution task

The Social Attribution Task (SAT) was proposed by Klin^20^ to study social attribution. It was used to measure the quality of answers in Heider and Simmel’s experiment on various groups, including neurotypical and autistic participants. The SAT has seven indices indicating different aspects of social attribution. We consider non-parametric tests as described above since the Shapiro–Wilk test shows evidence of non-normality for *VR* $1{\text {st}}$ and *VR* $2{\text {nd}}$ groups for the animation index ($W(26)=0.918$, $p=0.041$; $W(26)=0.913$, $p=0.030$), *screen* $1{\text {st}}$ for the cognition and affective index ($W(27)=0.909$, $p=0.022$; $W(27)=0.918$, $p=0.035$), and *VR* $2{\text {nd}}$ and *screen* $2{\text {nd}}$ for the person index ($W(26)=0.919$, $p=0.043$; $W(27)=0.908$, $p=0.020$).

**Figure 2:**
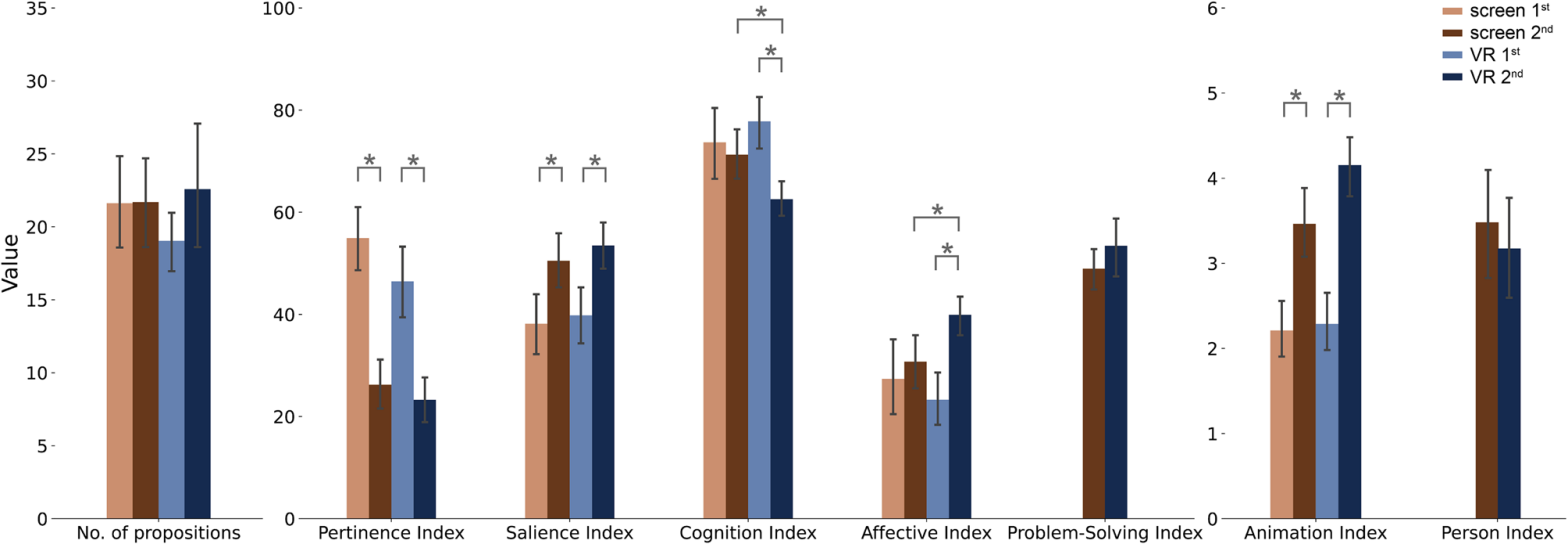
Results of the Klin indices^20^ for the *VR* and *screen* groups in the first and second impression. Asterisks mark significant differences. Error bars correspond to a 95% confidence interval.

We discuss here some selected effects summarized in Table 1, please refer to Sec. S6.2 in the supplementary for all pairwise differences. For a visual representation of the results, refer to Fig. 2.

##### Cognition and affective indices

 The cognition index measures the frequency of cognitive mental state terms used in the narrative (e.g., hiding, protecting, finding, etc.), while the affective index measures the frequency of affective mental state terms (e.g., happy, bullying, fighting, etc.). The indices are calculated by dividing the number of relevant propositions that include cognitive or affective terms by the total number of pertinent propositions. A proposition is considered pertinent if it includes at least one cognitive or affective term. Results show that the *VR* $2{\text {nd}}$ group reported less cognitive terms than the *screen* $2{\text {nd}}$ group ($U=194.500$, $p=0.044$). From this result, it can be hypothesized that when participants are explicitly encouraged to interpret the animation, those who watch it on a traditional screen report a story with a higher load of cognitive terms. This might be due to a higher familiarity with the *screen* or less sensory engagement compared to *VR*, which might prompt a more rational rather than emotional response. Additionally, the analysis shows that only the *VR* group decreased the cognition index in the second impression ($Z=-3.340$, $p=0.001$), which suggests that participants in the *VR* group reported less cognitive terms when they are encouraged to interpret the animation. Possible implications of this have to be discussed together with the affective index. For the affective index, results show that in the second impression the *VR* group reported a higher affective index ($U=192.500$, $p=0.044$). When looking jointly with the cognition index it can be hypothesized that the *VR* group reports a story with less cognitive terms but more affective ones, meaning that they report stories with more terms that involve emotions. The increased affective index in the *VR* group, especially on the second impression, indicates a stronger emotional connection with the content. This could be attributed to the immersive nature of VR. In general, participants describe the story involving more emotions, e.g., *the circle was afraid*, *the big triangle was hitting aggressively the small triangle*, etc. From this conclusion, one potential take-down is that the usage of VR to consume video content can make viewers empathize more with the actors, understanding their feelings and emotions. Regarding the interaction between time and modality, the affective index is influenced by the type of display ($U=224.000$, $p=0.037$). The *VR* group increased the affective index from 23.3% to 39.9% whereas the *screen* group only from 27.9% to 30.7%. The increase in the affective index over time in the *VR* group, as opposed to the *screen* group, suggests a cumulative effect of VR on emotional engagement. This could indicate that the longer participants engage with VR, the more emotionally connected they become to the content, potentially leading to more profound psychological connections with the content over time^36^.

##### Pertinence index

 Participants may include non-pertinent propositions (e.g., figures spinning around) while providing a narrative, which are not relevant to social attribution. The non-pertinent index does not only indicate the participants’ ability to explain the animation with relevant information, but also to the ability to inhibit reasoning processes that are not relevant in social attribution. Therefore, the score in this index corresponds to the percentage of propositions contained in the participant’s spontaneous narratives that were non-pertinent. Our analysis shows that both groups (*VR* $1{\text {st}}$ vs. *VR* $2{\text {nd}}$ and *screen* $1{\text {st}}$ vs. *screen* $2{\text {nd}}$) reduced the pertinence index in the second impression ($Z=-3.915$, $p<0.001$; $Z=-4.407$, $p<0.001$). This suggests that participants included less irrelevant information and focused more on relevant events when encouraged to interpret the animation.

##### Animation index

 The animation index, ranging from 0 to 6, indicates the participants’ capacity for attributing social meaning to ambiguous stimuli. A score of 0 indicates no social attribution, while a score of 6 indicates a high capacity for social attribution. This index is calculated hierarchically, considering terms that involve social attributions, such as behaviors, sensory experiences, emotions, or relationships. Our results indicate that both the *VR* and *screen* groups improved their animation index in the second impression ($Z=-4.185$, $p<0.001$; $Z=-3.808$, $p<0.001$). This suggests that participants improved their ability to give meaning to ambiguous stimuli when encouraged to interpret the animation.

##### Salience index

 The salience index indicates how many of the representative events participants have noticed in the animation, despite the diverse interpretations of its plot. There are a total of 20 identified events, and this index is calculated as the percentage of events detected out of the total number. The analysis reveals that both the *VR* and *screen* groups improved their salience index in the second impression ($Z=-3.284$, $p=0.003$; $Z=-3.088$, $p=0.003$). This suggests that participants become more aware of the key events during the second impression, presumably because they have gained prior knowledge from the first impression.

##### Person index

 Participants rate the personality of each shape, with scores ranging from 0 (no personality attribution) to 6 (high level of complex personality attribution). The index is hierarchical, taking into account physical properties of the shapes (e.g., big, small) and higher developmental features (e.g., curious, timid). This index is only calculated for the second impression. Results indicate no significant difference in the person index between the *VR* $2{\text {nd}}$ and *screen* $2{\text {nd}}$ groups, which may suggest that participants interpret each shape’s role similarly regardless of the device used to watch the animation.

##### Problem-solving index

 This index reflects the participants’ ability to provide accurate answers to the questions regarding the plot of the animation. It is computed by dividing the number of correct aspects in the participants’ answers by the total number of correct features. A value of 1 indicates that all questions were answered correctly, while a value of 0 indicates that no correct answers were provided. This metric is also only computed for the second impression. There is no evidence to suggest that the problem-solving index differs between groups (*VR* $2{\text {nd}}$ vs. *screen* $2{\text {nd}}$), suggesting that both groups may have interpreted the animation’s plot at the same level of accuracy.

**Table 1:** Results of the Klin indices^20^ for the *VR* and *screen* groups.

Numbers of propositions and SAT Index Scores	VR $1{\text {st}}$	screen $1{\text {st}}$	VR $2{\text {nd}}$	screen $2{\text {nd}}$	(VR $1{\text {st}}$ - VR $2{\text {nd}}$)	(screen $1{\text {st}}$ - screen $2{\text {nd}}$)
No. of propositions (SD)	19	21.3	22.6	21.7	− 3.5	− 0.1
	(5.4)	(8.0)	(11.0)	(8.0)	(9.8)	(8.7)
Pertinence Index (SD)	46.5%^*^	54.7%^†^	23.3%^*^	26.2%^†^	23.2	28.7
	(17.4)	(16.0)	(11.6)	(13.0)	(19.0)	(17.0)
Salience Index (SD)	39.8%^*^	38.3%^†^	53.5%^*^	50.5%^†^	-13.7	-12.3
	(13.6)	(14.9)	(12.0)	(13.5)	(17.5)	(16.5)
Cognition Index (SD)	77.8%^†^	73.1%	62.6%^*,†^	71.3%^*^	15.2	2.4
	(13.8)	(18.8)	(9.1)	(12.9)	(18.1)	(26.0)
Affective Index (SD)	23.3%^†^	27.9%	39.9%^*,†^	30.7%^*^	− 16.6 ^‡^	− 3.4 ^‡^
	(13.8)	(18.5)	(9.6)	(13.7)	(18.8)	(25.7)
Animation Index (0-6) (SD)	2.3^*^	2.2^†^	4.2^*^	3.5^†^	1.9	1.9
	(0.9)	(0.8)	(0.9)	(1.1)	(0.9)	(0.8)
Person Index (0-6) (SD)	–	–	3.2	3.6	–	–
	–	–	(1.6)	(1.7)	–	–
Problem-Solving Index (SD)	–	–	53.4%	49.6%	–	–
	–	–	(14.0)	(10.5)	–	–

The SD rows refer to the standard deviation of the corresponding upper row metric. The indices are computed for the first ($1{\text {st}}$), and second ($2{\text {nd}}$) impression (the person and problem-solving indices correspond to the answers given only in the second viewing). Matching superscripts in each row indicate statistically significant differences ($p<0.05$). Please see the main text for a discussion of the observed effects.

**Figure 3:**
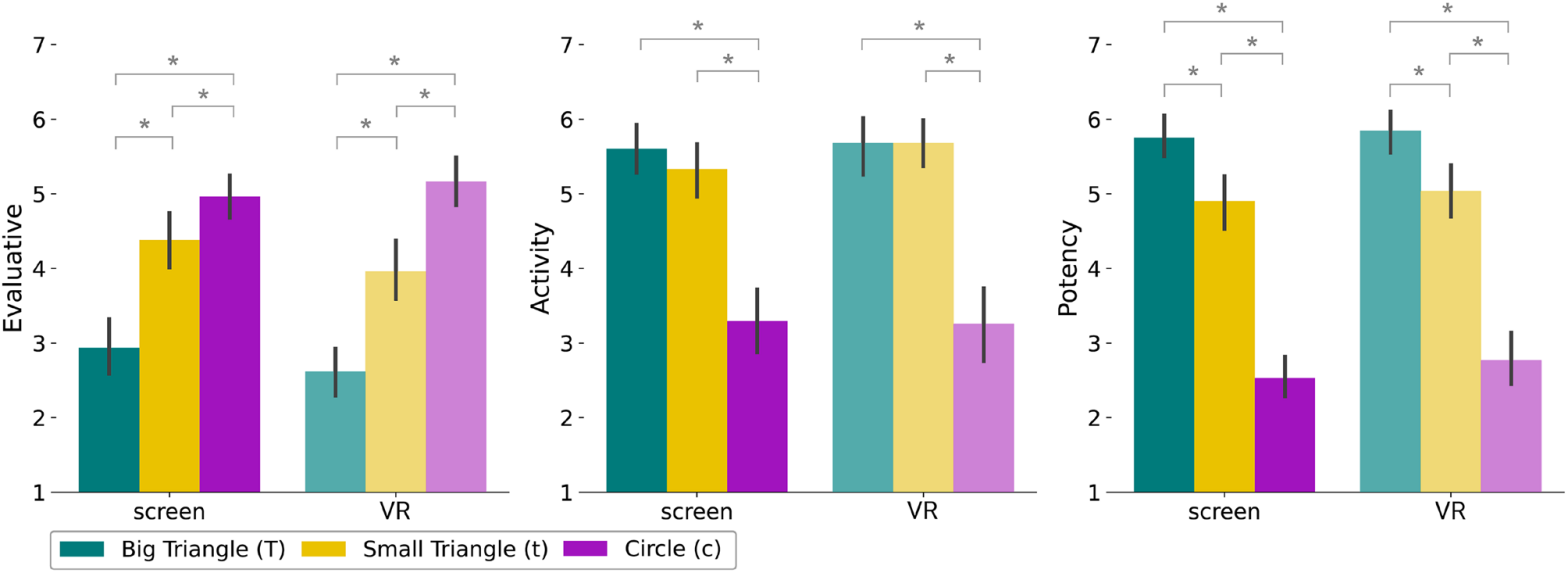
Responses for the personality traits grouped by cluster. **Left**: Evaluative cluster. A rating of 7 means that the shape is seen as the *nicest* while a rating of 1 means it is the most *bad*. **Center**: Activity cluster. A rating of 7 means that the shape is seen as the most *active* while a rating of 1 means it is the most *passive*. **Right**: Potency cluster. A rating of 7 means that the shape is seen as the most *potent* while a rating of 1 means it is the most *impotent*. Asterisks mark significant differences. Error bars correspond to a 95% confidence interval.

#### Greenberg-strickland questionnaire

The Greenberg-Strickland questionnaire^15^ rates each shape based on different personality traits, using a scale from 1 to 7. We consider three clusters (evaluative, activity, potency) with three traits in each cluster, and calculate the mean rating for each cluster (see Table 2). To prevent participant fatigue, we shorten the questionnaire and select a subset of traits based on high factor loading and meaningfulness^15^.

**Table 2:** Clusters of personality traits.

Evaluative	Activity	Potency
Bad–good	Passive–active	Fragile–tough
Foolish–wise	Moderate–violent	Cowardly–brave
Disreputable–reputable	Slow–fast	Weak–strong

Participants were asked to rate between 1 and 7 pairs of adjectives for describing the personality of each shape taking part in the animation, which are then grouped by clusters for the analysis.

We present the results for each cluster in Fig. 3. We consider non-parametric statistical tests, since Shapiro–Wilk test shows evidence of non-normality for the *VR* and *screen* groups for the *big triangle* *evaluative* ($W(26)=0.897$, $p=0.014$; $W(27)=0.880$, $p=0.005$), *small triangle* *activity* ($W(26)=0.806$, $p<0.001$; $W(27)=0.891$, $p=0.008$) attributes, the *VR* group for the perceived *circle* *activity* ($W(26)=0.879$, $p=0.006$), *circle* *potency* ($W(26)=0.910$, $p=0.026$) and *screen* *small triangle* *potency* ($W(27)=0.878$, $p=0.004$). We also run a Spearman’s Rank-Order Correlation analysis to investigate how these results relate to the SAT indices previously reported (Table S6.23 and Figure S6.17 in the supplementary).

##### Evaluative

 This pertains to traits assessing moral and intellectual qualities of the shapes, like bad-good, foolish-wise, and disreputable-reputable. In the *evaluative* cluster, both groups (*screen* and *VR*) rated the *circle* as nicer than the *small triangle* ($\mu _{screen, c}=4.963$, $\sigma _{screen, c}=0.991$, $\mu _{screen, t}=4.383$, $\sigma _{screen, t}=1.203$, $Z=-3.019$, $p=0.007$; $\mu _{VR, c}=5.167$, $\sigma _{VR, c}=1.245$, $\mu _{VR, t}=3.962$, $\sigma _{VR, t}=1.406$, $Z=-3.847$, $p=0.001$) and nicer than the *big triangle* ($\mu _{screen, c}=4.963$, $\sigma _{screen, c}=0.991$, $\mu _{screen, T}=2.938$, $\sigma _{screen, T}=1.391$, $Z=-3.448$, $p=0.002$; $\mu _{VR, c}=5.167$, $\sigma _{VR, c}=1.245$, $\mu _{VR, T}=2.615$, $\sigma _{VR, T}=1.061$, $Z=-3.877$, $p=0.001$), while the *small triangle* was rated as nicer than the *big triangle* ($\mu _{screen, t}=4.383$, $\sigma _{screen, t}=1.203$, $\mu _{screen, T}=2.938$, $\sigma _{screen, T}=1.391$, $Z=-2.684$, $p=0.014$; $\mu _{VR, t}=3.962$, $\sigma _{VR, t}=1.406$, $\mu _{VR, T}=2.615$, $\sigma _{VR, T}=1.061$, $Z=-2.617$, $p=0.015$). This can be observed in Fig. 3 (left). This ranking may be due to the *big triangle* being interpreted as *aggressive* and *evil* since it initiates the *fight*, whereas the *small triangle* tries to cover itself, and the *circle* does not participate in any *fight*. However, in the original work by Greenberg and Strickland, the *small triangle* was perceived as the nicest one^15^. One possible explanation for this difference could be that participants in the original experiment (50 years ago) perceived the *small triangle* as the nicest because it wanted to defend and protect the *circle*, while current participants may perceive the shape that does not participate in any fight as the nicest one. Although no statistically significant differences were observed between the groups, the *VR* group shows a more linear trend from *circle* to *small triangle* to *big triangle*, with clearer distinctions between each figure. The correlation analysis yields a negative correlation in the *VR* group between how nice is the *small triangle* and the cognition index in the second impression ($r_s(26)=-0.490, p=0.011$), but positive for the affective index ($r_s(26)=0.438, p=0.025$). This negative correlation between the niceness of the small triangle and the cognition index, coupled with its positive correlation with the affective index, may suggest that a more emotionally driven interpretation in VR leads to different trait attributions compared to a more cognitive approach. This could indicate that VR’s immersive experience shifts the viewer’s focus from analytical observation to emotional engagement.

##### Activity

 This cluster relates to dynamism and energy attributes, with traits like passive-active, moderate-violent, and slow-fast. For both groups (*screen* and *VR*), participants significantly rated the *circle* as less active than the *small triangle* ($\mu _{screen, c}=3.296$, $\sigma _{screen, c}=1.105$, $\mu _{screen, t}=5.333$, $\sigma _{screen, t}=1.054$, $Z=-4.256$, $p<0.001$; $\mu _{VR, c}=3.256$, $\sigma _{VR, c}=1.006$, $\mu _{VR, t}=5.679$, $\sigma _{VR, t}=1.048$, $Z=-3.917$, $p<0.001$) and less active than the *big triangle* ($\mu _{screen, c}=3.296$, $\sigma _{screen, c}=1.105$, $\mu _{screen, T}=5.605$, $\sigma _{screen, T}=0.743$, $Z=-4.233$, $p<0.001$; $\mu _{VR, c}=3.256$, $\sigma _{VR, c}=1.006$, $\mu _{VR, T}=5.679$, $\sigma _{VR, T}=0.670$, $Z=-4.272$, $p<0.001$). However, there is no significant evidence that the *big triangle* is more active than the *small triangle*, as shown in Fig. 3 (center). Notably, both groups ranked the *circle* as the least active shape. This may be because it is the shape that contributes the least to the animation, which could be interpreted as an attempt to avoid potential conflict. This ranking is consistent with the ratings of participants in the original work by Greenberg and Strickland^15^. The correlation analysis with the SAT indices reveals a positive correlation between the activity of the *small triangle* and both the affective and animation indices in the first impression ($r_s(26)=0.436, p=0.274$; $r_s(26)=0.466, p=0.017$). This suggests that participants in the *VR* group perceive the small triangle as more active when they attribute more human-like qualities to the animation. For the *circle*, there is a negative correlation to the problem-solving index in the *VR* group ($r_s(26)=-0.409, p=0.038$). This suggests that participants might view the circle as less central to the plot development or problem-solving within the animation. This could be due to its perceived passivity or non-involvement in the main action, highlighting how the role of different shapes is differently interpreted in the *VR* group in terms of narrative importance. These results highlight VR’s potential to distinctly affect narrative engagement and character interpretation in storytelling.

##### Potency

 This focuses on the strength and assertiveness aspects, with traits including fragile-tough, cowardly-brave, and weak-strong. Participants in both groups (*screen* and *VR*) rated the *circle* as less potent than the *small triangle* ($\mu _{screen, c}=2.531$, $\sigma _{screen, c}=0.833$, $\mu _{screen, t}=4.901$, $\sigma _{screen, t}=1.140$, $Z=-4.125$, $p<0.001$; $\mu _{VR, c}=2.769$, $\sigma _{VR, c}=1.377$, $\mu _{VR, t}=5.038$, $\sigma _{VR, t}=1.279$, $Z=-3.762$, $p<0.001$) and less potent than the *big triangle* ($\mu _{screen, c}=2.531$, $\sigma _{screen, c}=0.833$, $\mu _{screen, T}=5.753$, $\sigma _{screen, T}=0.712$, $Z=-4.547$, $p<0.001$; $\mu _{VR, c}=2.769$, $\sigma _{VR, c}=1.377$, $\mu _{VR, T}=5.846$, $\sigma _{VR, T}=0.723$, $Z=-4.227$, $p<0.001$). The *big triangle* was rated as more potent than the *small triangle* ($\mu _{screen, T}=5.753$, $\sigma _{screen, T}=0.833$, $\mu _{screen, t}=4.901$, $\sigma _{screen, t}=1.140$, $Z=-2.912$, $p<0.001$; $\mu _{VR, T}=5.846$, $\sigma _{VR, T}=0.723$, $\mu _{VR, t}=5.038$, $\sigma _{VR, t}=1.279$, $Z=-2.309$, $p=0.033$). These results are shown in Fig. 3 (right). Both groups ranked the *circle* as the least powerful shape, possibly because participants could not evaluate its potency due to its lack of direct interaction with other shapes. The *small triangle* was ranked higher than the *circle*, and the *big triangle* was ranked the highest. This could be due to the larger size of the *big triangle*, making it more difficult for the *small triangle* to push it in any of the *fights*. This ranking is also consistent with the one from Greenberg and Strickland^15^. The correlation analysis shows a negative relationship between the potency of the *big triangle* and the pertinence index in the second impression in the *VR* group ($r_s(26)=-0.592, p=0.001$), suggesting that when participants attribute greater potency to the big triangle, they provide narratives with fewer extraneous details. Conversely, a positive correlation is observed when comparing it with the *circle* ($r_s(26)=0.471, p=0.015$). This suggests that participants may prioritize different character traits in their narratives. Specifically, when the big triangle is perceived as more potent, participants streamline the narrative, while a positive correlation with the circle implies that when the circle is viewed as more potent, participants may emphasize different narrative aspects. The correlations offer insights into how character traits influence narrative focus in the VR experience.

The interaction between the shape (*big triangle*, *small triangle*, *circle*) and the modality (*VR*, *screen*) does not yield any statistically significant findings. Similarly, when exploring the three-way interaction considering also the clusters (*evaluative*, *activity*, *potency*), no significant results are observed. For the detailed analyses, please refer to Table S6.13 in the supplementary.

Aside from these observed differences, the fact that there are no significant differences in rankings between the screen *screen* and *VR* groups (see Table S6.12 in the supplementary) suggests that the medium does not drastically alter the perceived roles of the shapes in the narrative. This indicates a fundamental consistency in how participants, regardless of the viewing medium, interpret the characters’ roles in the animation.

##### Correlations between shapes and clusters

 We compute the Spearman’s Rank-Order Correlation coefficient for a correlation analysis of the various figures and clusters. Here, we present the key findings, while all correlations are available in the Table S6.23 and Figure S6.16 in the supplementary. With respect to the same figure and different clusters, there is a negative correlation for both groups (*VR* and *screen*) between the activity of the *big triangle* and the evaluative attribute ($r_s(26)=-0.452, p=0.020$; $r_s(27)=-0.602, p=0.001$). This is, the *big triangle* is usually seen as less nice and more active. For the evaluative and activity attributes of the *small triangle*, there is only a negative correlation for the *screen* group ($r_s(26)=-0.630, p<0.001$). When analyzing the results of the correlations between different figures in the same cluster, we find that there is a negative correlation for both groups (*VR* and *screen*) between the evaluative attribute of the *big triangle* and the *small triangle* ($r_s(26)=-0.747, p<0.001$; $r_s(27)=-0.480, p=0.011$), the *big triangle* and the *circle* ($r_s(26)=-0.671, p<0.001$; $r_s(27)=-0.414, p=0.032$), and the *circle* and the *small triangle* ($r_s(26)=-0.634, p<0.001$; $r_s(27)=-0.618, p=0.001$). This trend was consistent for both the *VR* and *screen* groups, suggesting a clear distinction in how participants rated the niceness of each figure. Additionally, when participants perceived the circle as active, they also perceived the big triangle as more active, both in the *VR* ($r_s(26)=-0.622, p=0.001$) and *screen* ($r_s(27)=-0.523, p=0.005$) groups. This implies a relationship between character attributes, especially regarding activity. Within the potency cluster, a negative correlation emerged between the *circle* and the *big triangle* specifically in the *VR* group ($r_s(26)=-0.469, p=0.016$). This finding suggests an interplay between character attributes, particularly in the context of VR, where perceiving the big triangle as more potent coincided with viewing the circle as less potent. This observation emphasizes the distinctive impact of the HMD on character perception.

### Participant congruency: Is viewing behavior similar between participants?

**Figure 4:**
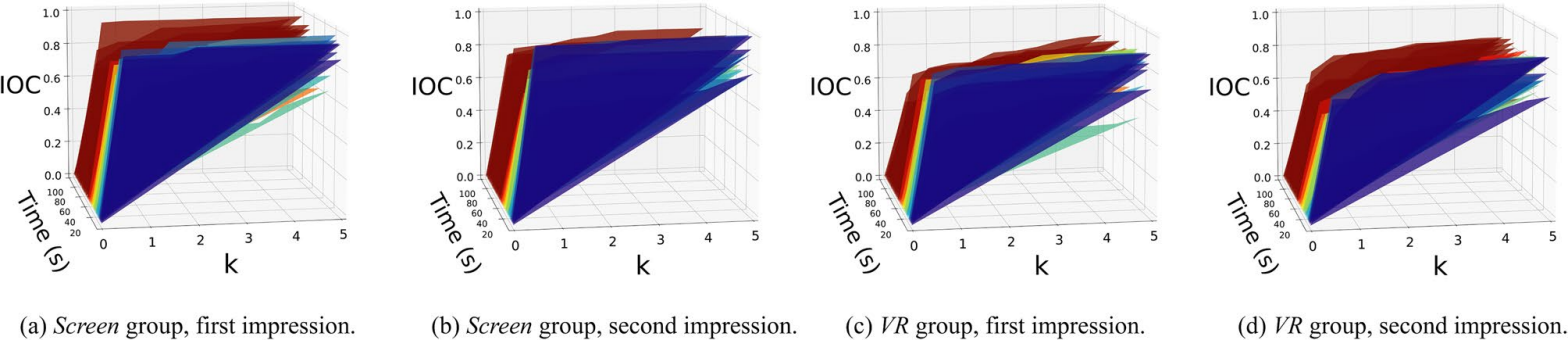
IOC curves for the *screen* and *VR* groups for the first and second impression. Each time slice is color coded, *k* refers to the percentage threshold used to define the most salient regions in the saliency map. The IOC can be interpreted as the agreement in the regions observed by the participants over time while watching the animation (a higher value indicates higher agreement). In all visualizations the agreement of participants was high, indicating that they were observing roughly the same regions of the scene and therefore were following the plot similarly.

In this section, we describe how eye-tracking data was used to assess the similarity in viewing behavior between participants in the same group and whether it is related to our previous findings.

We first compute the *inter-observer congruence* (IOC) metric by means of a *receiver operating characteristic curve* (ROC)^37^. This metric calculates the ability of the $i_{\text {th}}$ participant to predict the *saliency map* (a heatmap that represents which areas of the scene are more prone to be observed by a user) composed from the fixations of all the other participants averaged. Following previous work^29,30^, we use a leave-one-out approach where the $i_{\text {th}}$ participant is excluded and the saliency maps of the other participants are combined by accumulating two-second windows. The percentage of gaze points of the $i_{\text {th}}$ participant that falls within the k% most salient regions predicted by the saliency map is then computed for $k \in [0\%..5\%]$ in increments of 0.5. This process is repeated for all participants, and the mean value is calculated. Intuitively, the IOC gives an estimation of how well other participants’ data approximate the behavior of the $i_{\text {th}}$ participant. A high value of this metric indicates that most of the participants are viewing the same regions of the scene whereas a low value indicates that participants are scattered watching different regions. Figure 4 shows the IOC curves computed for the *screen* and *VR* groups for the first and second impression. In the *VR* group, IOC values show a slight difference compared to the *screen* group. This variation may be due to the fact that participants in VR had more mobility, while those in the other group had a more stationary experience. Nevertheless, in general, it can be observed that for both groups the IOC values are very similar and high during the whole animation, this indicates a high agreement of the regions observed by the participants. Additionally, we provide in the supplementary videos showing the saliency maps and gaze points from participants of the *VR* and *screen* groups for the first impression and second impression.

**Figure 5:**
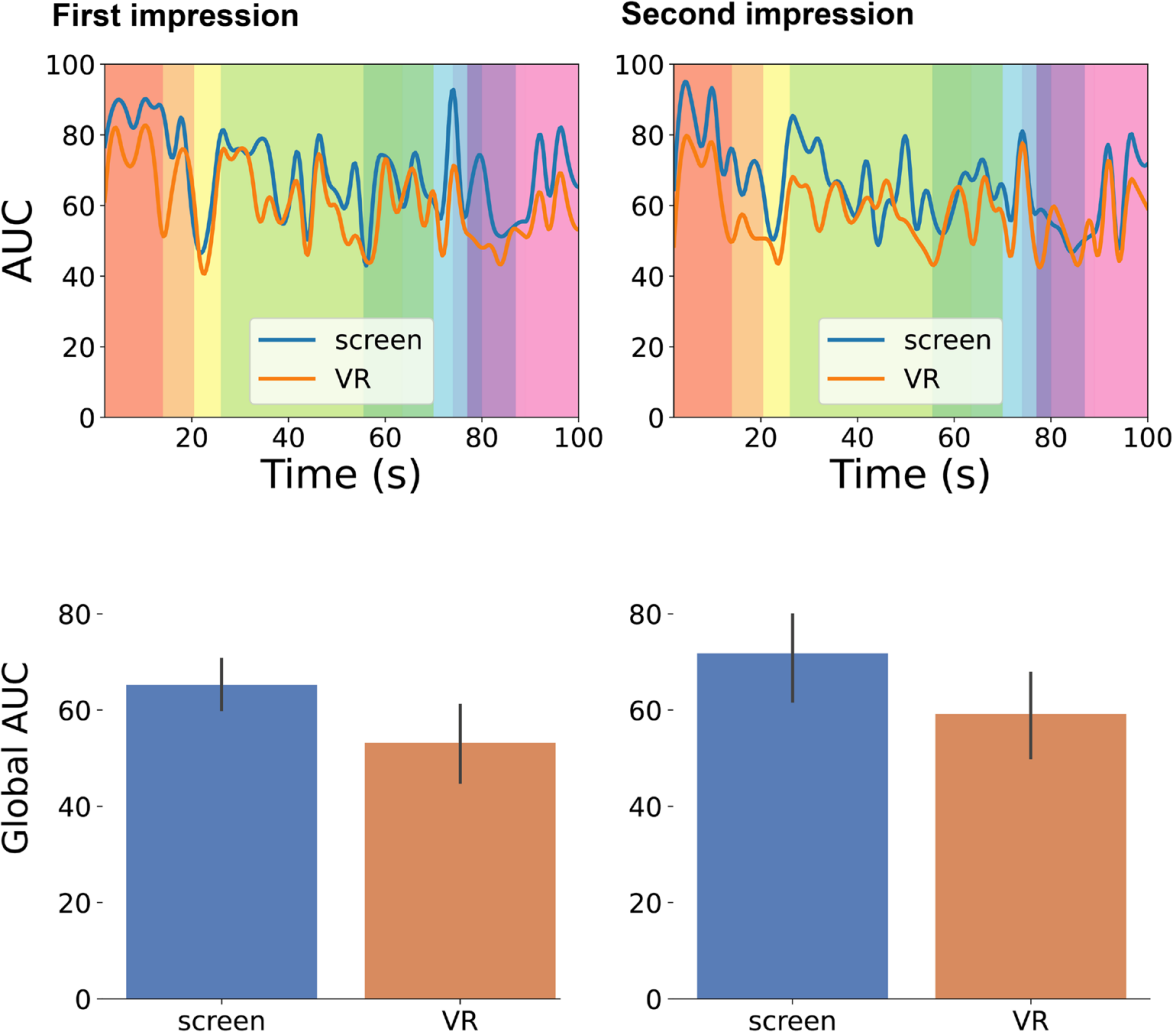
**Top**: AUC curves for the *screen* and *VR* groups for the first and second impression. Different background colors code different scenes in the animation. For instance, in the third scene (yellow), the *big triangle* opens the door and moves out towards the *small triangle*. In this case, the AUC drops because participants have to distribute their attention between these two shapes. In contrast, in the first scene (red), the *big triangle* is moving into the house and it is alone in the scene, attracting all the participants’ attention. **Bottom**: Global AUC metric for the *screen* and *VR* groups for the first and second impression. Error bars correspond to a 95% confidence interval.

To provide an easier interpretation of the evolution of the IOC over time, the Area Under the Curve (AUC) is computed for the IOC curves. Following the interpretation of the IOC, the AUC takes values between 0 (no congruency between subjects) and 100 (total congruency)^30,31^. The resulting curves are shown in Fig. 5 (top). Each colored region represents a meaningful scene of the plot. It can be seen that there are variations along with the animation. Low peaks, where participants are observing different animation elements, are usually produced because they have to distribute their attention among the three shapes since they are performing different actions. AUC remains high when there is only a shape on stage or when only a shape is acting. In general, we do not observe prominent differences between the *VR* and the *screen* group, indicating that in both groups participants were roughly following the plot similarly. However, around 20-30s the *VR* group decreased its AUC with respect to the first impression. In this part of the animation, the *fight* between the *big triangle* and the *small triangle* is taking place. The fact that VR participants focused on different parts of the scene during this particular moment suggests their active efforts to gather information about the various figures and gain insights into their behavior.

To compare more specifically whether there are significant differences in the agreement of the participants when viewing the animation, we compute the AUC for each participant globally, i.e., when selecting a time window spanning the whole animation duration. Results are shown in Fig. 5 (bottom). Shapiro–Wilk test shows evidence of non-normality for *VR* $1{\text {st}}$ group, and *screen* $2{\text {nd}}$ group ($W(26)=0.898$, $p=0.014$; $W(26)=0.735$, $p<0.001$, respectively). Although there is no significant difference, the trend in the results seems to suggest that participants who watched the animation in VR show less agreement in the second impression than those who watched it through the screen ($\mu _{VR\ 2\text{nd}}=59.172$, $\sigma _{VR\ 2\text{nd}}=23.353$, $\mu _{screen\ 2\text{nd}}=71.797$, $\sigma _{screen\ 2\text{nd}}=23.582$, $U=227.500$, $p=0.043$). This observation suggests that participants in the *VR* group may indeed be more inclined to explore different aspects of the scene in the second impression. This behavior could be indicative of increased engagement with the content and could be related to the stronger emotional connection observed in the affective index. Participants who feel a stronger emotional connection to the content may be more motivated to explore various aspects of the scene to immerse themselves further in the narrative. Therefore, the increased emotional connection observed in the *VR* group might be a contributing factor to their heightened exploratory behavior in the second impression. This connection between emotional engagement and exploratory behavior could be a potential avenue for further investigation to better understand the dynamics of viewer interaction with immersive content.

Our analysis does not show significant evidence, but a tendency that the *screen* group increased their agreement in the second impression ($\mu _{screen\ 1{\text{st}}}=65.201$, $\sigma _{screen\ 1{\text{st}}}=13.187$, $\mu _{screen\ 2\text{nd}}=71.797$, $\sigma _{screen\ 2\text{nd}}=23.582$, $Z=-2.085$, $p=0.078$). This again is indicative of a less exploratory behavior in the screen condition. Please refer to Table S6.14 in the supplementary for these results. We also did not observe significant differences in the interaction of time (first impression, second impression) vs. modality (*screen*, *VR*) ($U=297.000$, $p=0.598$).

### Viewing behavior: Do participants’ viewing behaviors differ in VR and 2D displays?

In order to detect differences in the visual and attentional behaviors between the two visualization groups, we propose a set of metrics and indicators that leverage the eye-tracking information. We do not consider pupil dilation in our analysis as controlling light conditions during the experiment was challenging, especially within the VR environment. Our observations consistently showed smaller pupil sizes in the VR condition, likely influenced by the high brightness of the HMD display.

#### Figure saliency

**Figure 6:**
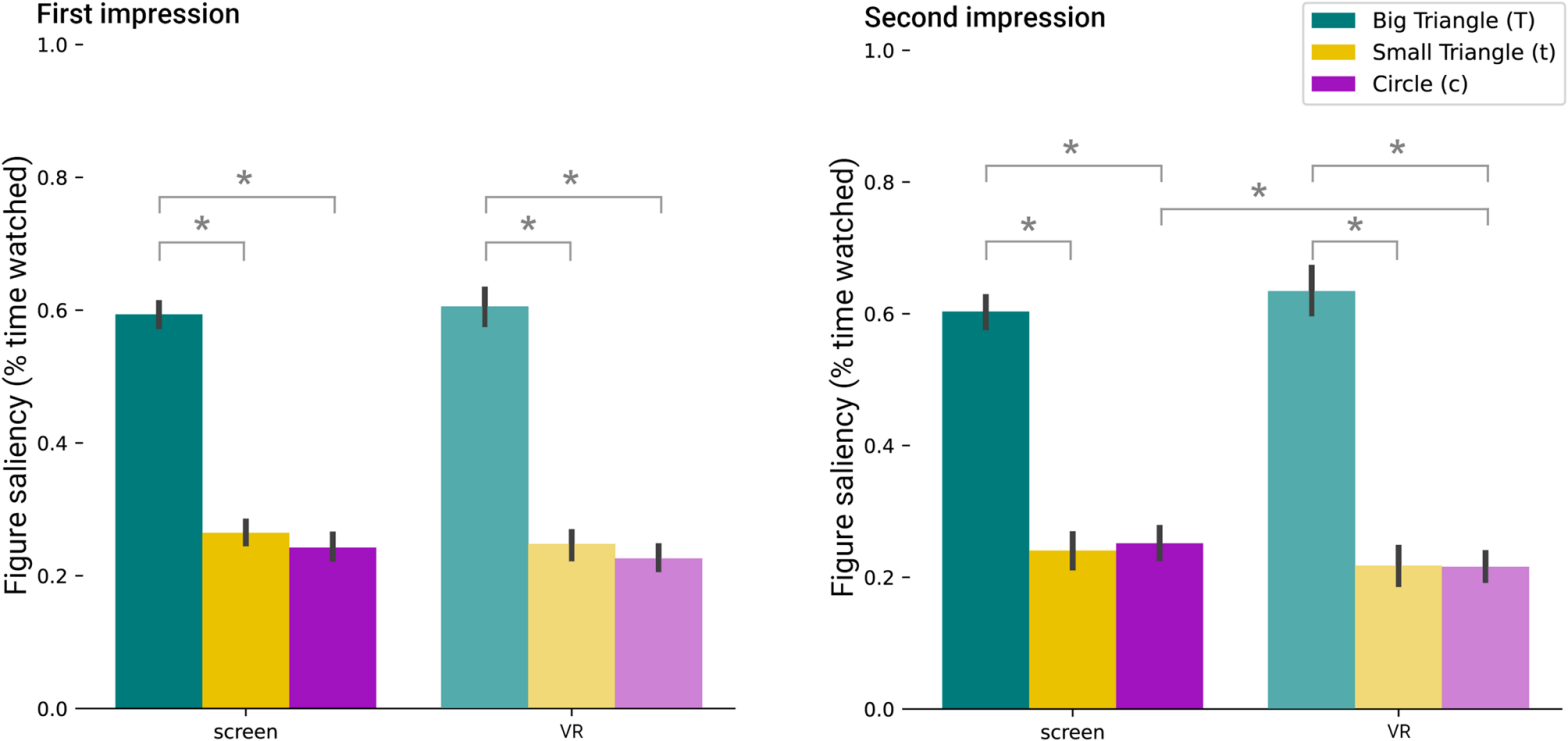
Figure saliency. Mean across participants of the percentage of time watching a shape in each group for the first (left) and the second (right) time they watch the animation. Asterisks mark significant differences. Error bars correspond to a 95% confidence interval.

 This metric computes the percentage of time spent watching the different shapes in the animation. This is indicative of the participants’ interest in each of the shapes in a time unit. The gaze location is used to compute this metric, which is provided by the eye-trackers. Note that the eye-tracker does not have perfect accuracy, so a confidence area of 4 degrees of visual angle is used^38^. This implies that the total sum of the percentages does not always add up to 1. This metric can be computed for the full animation and also for each of the twelve scenes (see Sec. S6.4.2 in the supplementary for the results per scene and the pairwise differences). Results are shown in Fig. 6. The Shapiro–Wilk test shows evidence of non-normality for the *screen* group for the perceived figure saliency of the *circle* in the first ($W(26)=0.890$, $p=0.009$) and second ($W(26)=0.884$, $p=0.007$) impressions, therefore, we again resort to non-parametric tests.

Overall, the rankings of figure saliency remain the same (*big triangle* > *small triangle* > *circle*) regardless of the device used and the first or second impression. This may suggest that the type of display does not play a role when deciding which shape to observe. Note that in this animation, all action takes place in a reduced field of view, so even when using an HMD device for VR, participants’ are able to see all the action in the same field of view. We believe this insight may not hold for the VR condition if the action were scattered around the $360^{\circ }$ of the scene. In that case, visual content^29,30^ or directional sound^39^ may play a strong role in guiding subjects’ attention. We do not observe significant differences for the two-way interaction of time (first impression, second impression) vs. modality (*screen*, *VR*) nor for the three-way interaction (including also the figure). Please refer to Table S6.17 in the supplementary for the results.

#### Number of figure changes

**Figure 7:**
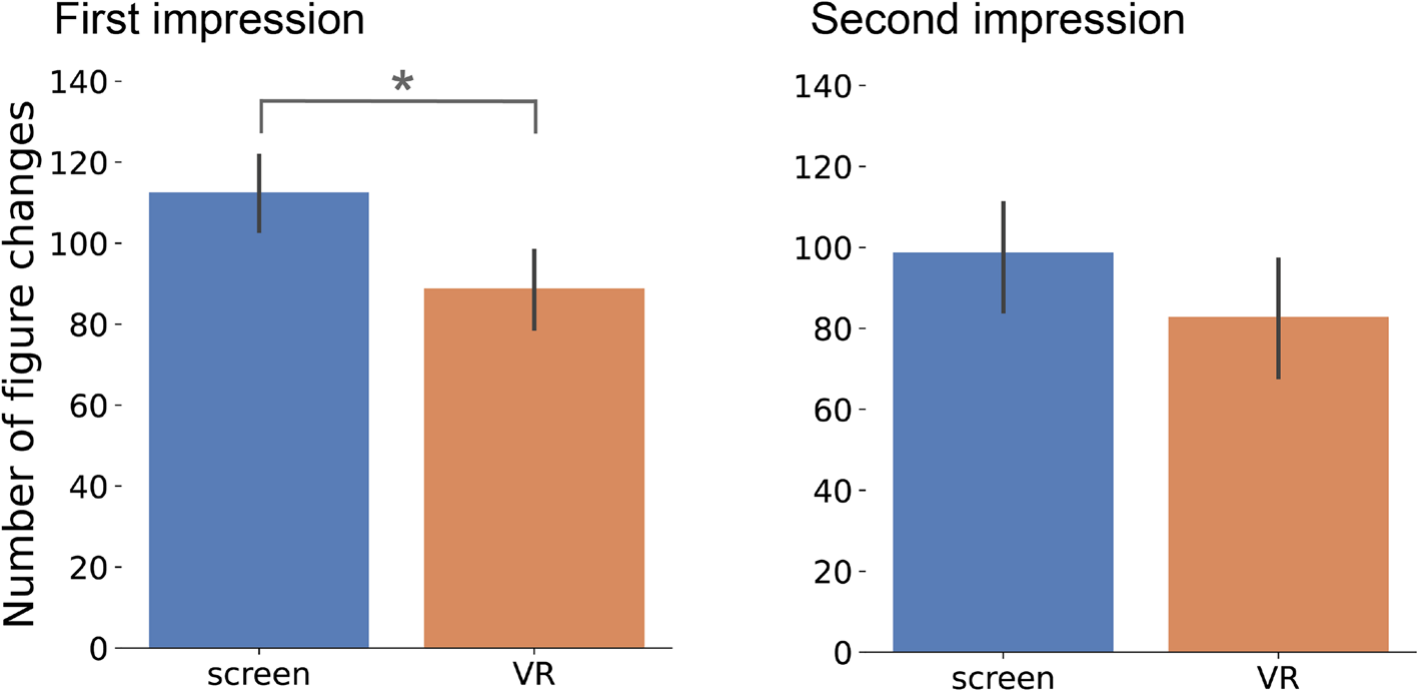
Number of figure changes. Mean across participants of the number of times that they switch from observing one shape to another for the first (left) and second (right) time they watch the animation. Asterisks mark significant differences. Error bars correspond to a 95% confidence interval.

 This metric quantifies the frequency of changes in the participant’s gaze from one shape to another, indicating the level of interest in each shape. If a participant does not fixate on any shape, it is assumed that they are still fixated on the previous shape. Similarly to the previous metric, this metric can be computed for the full animation and also for each of the twelve scenes (see Sec. S6.4.3 in the supplementary for the results per scene and all the pairwise differences). Results for this metric are shown in Fig. 7. Again, the Shapiro–Wilk test shows evidence of non-normality for the *screen* group in the second impression ($W(26)=0.874$, $p=0.004$). Results suggest that participants who watched the animation in VR performed less changes that those who watch it through the regular screen ($\mu _{VR\ 1{\text{st}}}=88.808$, $\sigma _{VR\ 1{\text{st}}}=24.712$, $\mu _{screen\ 1{\text{st}}}=112.538$, $\sigma _{screen\ 1{\text{st}}}=23.670$, $U=164.000$, $p=0.006$) during the first impression. This may indicate that participants are more engaged with the action, switching their attention between the characters only when meaningful events occur. Detailed scene-wise analysis reveals that the difference in gaze shifts between *VR* and *screen* users was significant in most scenes during the first impression (refer to the supplementary for all test results), confirming a consistent pattern of engagement of the VR participants except for scene two (apparition of the *small triangle* and *circle*) and nine (the *big triangle* opens the door and comes out of the house). In contrast, during the second impression, there are no significant differences in gaze shifts between VR and screen users across all scenes ($\mu _{VR\ 2\text{nd}}=82.769$, $\sigma _{VR\ 2{\text{nd}}}=40.127$, $\mu _{screen\ 2\text{nd}}=98.731$, $\sigma _{screen\ 2^{\text{nd}}}=35.590$, $U=237.500$, $p=0.108$). This may indicate that, as users become more familiar with the content, the influence of the viewing modality on gaze behavior diminishes.

Results do not show evidence that the number of changes is different from the first to the second impression for the same group. Additionally, the interaction of time (first impression, second impression) vs. modality (*screen*, *VR*) is also not significant ($U=307.000$, $p=0.734$).

### Viewing-personality correlation: How does viewing behavior relate to the perception of the elements in the scene?

To study the relationship between participants’ subjective perceptions and their objective gaze patterns, we conduct a correlation analysis, considering the SAT indices, Greenberg-Strickland questionnaire, global AUC, figure saliency, and the number of figure changes. To define the degree of relationship between variables, we consider the guidelines established by Dancey and Reidy^40^, where a Spearman coefficient ($r_s$)>= 0.7 is considered a strong correlation,>= 0.4 moderate, and >= 0.1 weak. Here we only report those relevant insights that have at least a moderate correlation. Please refer to the Table S6.21 and Figure S6.15 in the supplementary for all the pairwise correlations.

#### Distinct perceptual dynamics in VR and screen viewing

 In the *VR* group, our data shows a moderate, negative correlation between the circle’s perceived activity and figure changes ($r_s(26)=-0.438, p=0.025$). This might suggest a trend where more figure changes are associated with attributing less activity to the circle in VR, indicating a more distinct perception enabled by the immersive VR environment. In the *screen* group, the positive correlation between the small triangle’s activity and global AUC ($r_s(26)=0.470, p=0.015$) could indicate that higher agreement levels are associated with a perception of higher activity, suggesting a more straightforward perceptual process compared to the more complex engagement observed in VR. The *screen* group also exhibits a moderate, negative correlation between the circle’s evaluative attribute and global AUC ($r_s(26)=-0.418, p=0.033$), and a positive correlation between the pertinence index and global AUC ($r_s(26)=0.466, p=0.016$). These correlations suggest possible trends in viewing agreement and character perception, suggesting a more superficial engagement with the narrative in the screen setting.

#### Enhanced narrative engagement in VR

 This is particularly evident in the diverse viewing patterns observed in participants in the *VR* group. For example, we observe a moderate, negative correlation between the problem-solving index and the global AUC in the first impression among participants in the *VR* group ($r_s(26)=-0.476, p=0.014$), suggesting that those with higher problem-solving scores, reflecting a better grasp of the plot, engaged with the narrative in a more complex manner, as indicated by their varied viewing patterns. Additionally, the negative correlation between the pertinence index and the time spent watching the small triangle in the second impression ($r_s(26)=-0.562, p=0.003$) suggests that participants in the *VR* group may be focusing more on narrative-relevant elements, leading to a deeper engagement with the story.

#### Narrative processing and attention in VR

 The immersive nature of VR seems to encourage a holistic processing of the narrative. This is supported by the participants’ ability to notice and interpret complex story elements accurately. The negative correlation in the *VR* group between the pertinence index and figure changes ($r_s(26)=-0.529, p=0.005$) suggests that VR participants, when exposed to more figure changes, tended to report less irrelevant information. This implies a focused and comprehensive engagement with the narrative in VR. In contrast, the *screen* group showed a different pattern of narrative interaction. More figure changes in this group correlated with increased irrelevant information ($r_s(25)=0.421, p=0.036$) and reduced recognition of key events as denoted by the salience index ($r_s(25)=-0.415, p=0.039$), indicating a less focused narrative comprehension. Furthermore, in the *screen* group, the positive correlations between the small triangle’s evaluative attribute and viewing time ($r_s(26)=0.422, p=0.032$), and between the circle’s perceived activity and time spent watching the big triangle ($r_s(26)=0.445, p=0.023$), imply that prolonged observation of one element influenced the perception of another. This highlights a dynamic interplay in shape assessment, contrasting with the more singular narrative focus observed in VR.

### Presence: Is there a difference in the feeling of presence between VR and 2D displays?

**Figure 8:**
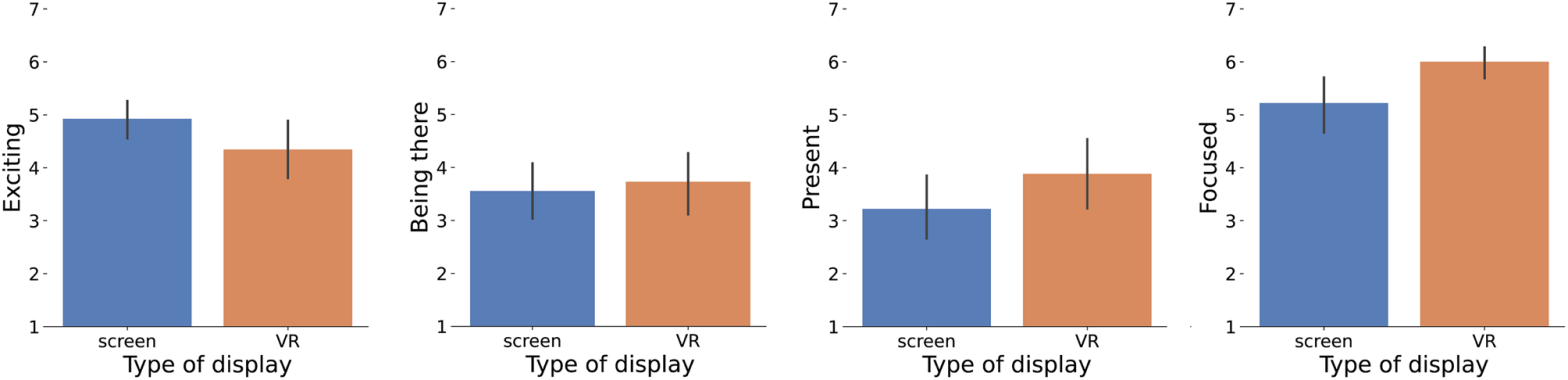
Mean across participants for the questions in the presence questionnaire performed at the end of the experiment. Answers for each question are between 1 and 7. From left to right: answer to the question “*How exciting was the animation?*”; answer to the affirmation “*Watching the animated scene I had the feeling of being there*”; answer to the affirmation “*I felt present in the virtual scene*”; and answer to the question “*To what extent were you aware of the real world around you while viewing the animation?*” where a 7 means that the participant was less aware and more focused. Error bars correspond to a 95% confidence interval.

To assess the participants’ feeling of presence, we ask a subset of questions from the Igroup Presence Questionnaire (IPQ)^41^ at the end of the experiment (see Sec. S2 in the supplementary for the questionnaire).

Results for these questions are displayed in Fig. 8. Please refer to Sec. S6.6 in the supplementary for the significance values of all the comparisons.

Results show a possible trend where participants appear to be more focused on the animation in VR. A potential interpretation could be that the immersive nature of the VR environment may have reduced distractions from the external surroundings, possibly contributing to increased attention to the task. For the questions regarding *being there* and *being present* there are no significant differences but the trend seems to favor the visualization in VR. On the other hand, for the question regarding *how exciting the scene was* the trend seems to favor the visualization on the screen. During the debriefing sessions, we found that it is possible that participants may have perceived the screen as a more traditional way of viewing content and expected a more passive experience, while the use of VR may have raised expectations for a more immersive and engaging experience. This may have influenced their perception of the excitement level of the animation, leading to the observed trend in responses.

## Discussion

In this work, we have extended the classic Heider and Simmel experiment into the VR realm and we have explored the implications of using VR devices in social meaning attribution. Our study provides both quantitative and qualitative data, and we have relied on existing metrics while also introducing novel ones for analyzing participants’ behavior and answers.

Our results suggest that there are differences in social meaning attribution depending on the device used, with the VR device eliciting stronger emotional connections and engaging narratives for the shapes in the animation. This effect may be due to the immersion capabilities of VR, rather than differences in viewing patterns imposed by the visualization conditions, as suggested by our eye-tracking metrics.

When comparing our results to the original Heider and Simmel experiment, we observe some differences in the participants’ first impressions. During the debriefing session following the experiment, several participants commented on their curiosity regarding the very simple stimuli of the experiment. In reflecting upon the experimental results, it is plausible to consider the evolution of computer graphics over the past 80 years may have played a role. Eight decades ago, when exposure to computer-generated imagery was limited, participants might have readily accepted and engaged with simpler animations. Their lack of familiarity with complex graphics could have led them to interpret these basic animations more in terms of real-life scenarios, whereas our participants might have perceived the same simple animations differently, especially in their first impression, when they were expecting something more complex. However, our results for the *screen* group do match those of Heider and Simmel when participants are encouraged to express the narrative in terms of animated beings in their second impression. Nevertheless, please note that this does not affect our insights, since we compare in our experiment both devices (*VR* and *screen* groups) in the same conditions.

As a main conclusion, our study suggests that the sense of presence provided by VR impacts social meaning attribution for abstract stimuli. In particular, participants who viewed the animation in VR placed greater emphasis on perceived feelings and formed stronger emotional connections with geometric shapes than those who viewed it on a 2D display. This observation is supported by the significant differences in cognition and affective indices. These findings have potential implications for the fields of psychology, games, and cinematic VR. For example, our results suggest that VR could be used as a tool for investigating social meaning attribution and other related cognitive processes, as well as for creating more engaging and immersive gaming and cinematic experiences. VR may offer a more immersive and engaging experience for participants, which could lead to more accurate and detailed assessments of social perception and interaction.

### Limitations and future work

 Similarly to other studies of the same nature, our results are only strictly valid for our chosen stimuli. In this work we have used a simple animation composed of geometrical shapes, as proposed by Heider and Simmel^1^ to avoid confounding factors such as the complexity of the narrative or the realism of the characters. Further research in this direction is needed to investigate how the derived insights extend to more complex narratives or visual content. Many other variables and parameters can also be explored in future work, such as the influence of color, field of view, point of view, and camera motion, or sound.

## Methods

### Stimulus and apparatus

Participants watched an accurate recreation of the original Heider and Simmel experiment^1^ (see the supplementary for the video of the animation). The animation has a duration of two minutes in which three geometric shapes (a big triangle, a small triangle, and a circle) move in various directions and at various speeds through the display. In the animation, there is also an enclosed rectangle, and one of the *walls* has a section that actuates as a *door*. The animation is kept simple to avoid confounding factors such as the realism of the animation or the physics of complex interactions.

The animation can be understood as a composition of scenes where each scene is represented by a certain shape’s behavior. For simplicity, the big triangle is called *T*, the small triangle is called *t*, the circle is called *c*, and the rectangle is called *house*. Note that the stimulus is composed of simple animations and interactions between the geometric shapes with no further meaning. For facilitating the reading, the twelve scenes are described attributing certain subjective interpretations:

1. *T* moves towards the house, opens the door, moves into the house, and closes the door.
2. *t* and *c* appear from above and move around near the door.
3. *T* opens the door and moves out the house toward *t*.
4. *T* and *t* fight. *T* wins. During the fight, *c* moves inside the house.
5. *T* moves into the house, closing the door.
6. *T* chases *c* within the house while *t* is moving along outside of the house towards the door.
7. *t* opens the door and *c* runs out of the house. Both close the door.
8. *T* tries to get out of the house while *t* and *c* are moving in circles outside the house and touching each other several times.
9. *T* opens the door and comes out of the house.
10. *T* chases *t* and *c* twice around the house.
11. *t* and *c* run away, disappearing from the viewer’s view.
12. *T* hits the walls of the house several times, breaking it.

The animation is implemented in the Unity game engine. In the traditional screen the figures are two-dimensional while in VR the figures have depth so that the figures resemble a more natural interpretation of the 3D world. In the three-dimensional version, the original two-dimensional elements become three-dimensional elements, i.e., the triangles are tetrahedrons, the circle is a sphere and the house walls gain the high dimension. The animation is projected in front of the participants so that they do not have to roam around the $360^{\circ }$ in VR to find the action. The movements of figures remain in the same axis to ensure that moving along the new axis (depth) does not influence the results. The relative size of each element is adjusted for both devices so that they cover the same degrees of visual angle and ensure the same for all the participants (Sec. S5 in the supplementary). Note that the goal of our research is to compare these two media, therefore we keep the experimental conditions in VR as close as possible to the 2D display counterpart.

The traditional screen is a 21-inches display with a resolution of $1920 \times 1080$ pixels, equipped with a Grazepoint GP3 eye-tracker recording at 150 Hz. Participants sat down and used a chinrest to keep their position fixed and ensure accurate eye-tracking after the calibration. The VR device is an HTC Vive Pro with a nominal field of view of $110^{\circ }$, a resolution of $1440 \times 1600$ pixels per eye, and a frame rate of 90 fps equiped with a binocular eye-tracker from pupil-labs. It records data at 200 Hz but the logging frequency is limited to the animation frame rate played in Unity, which is 60 fps. Participants sat down on a rotating stool. Both eye-tracking devices have a theoretical spatial accuracy of 1 degree of visual angle but in practice 4 degrees have been used as the confidence interval to account for calibration errors and small displacements while performing the study^38^.

### Participants

A total of 53 participants took part in the study, 27 watched the stimuli on a traditional screen (14 female and 13 male, average age 23.6 years old, $\sigma =6.2$) and 26 watched the stimuli through a VR device (13 female and 13 male, average age 23.9 years old, $\sigma =4.8$). From this last group, sixteen of them had tried VR before, and none of them used VR frequently. None of the participants reported a neurodivergent diagnosis. All participants were economically compensated and provided written consent for their voluntary participation in the study. They were naïve about the final purpose of the experiment, and they all reported normal or corrected-to-normal vision and audition. The research protocol was approved by the Regional Ethics Committee of Aragon (CEICA) and the Data Protection protocol was approved by the University of Zaragoza.

### Design and procedure

Before starting with the experiment, the experimenter explained the experiment procedure and asked participants to sign the informed consent. Each participant completed a brief demographic questionnaire (Sec. S1 in the supplementary) and was randomly assigned one group (*screen* or *VR*). For the *screen* group, the chinrest was adjusted comfortably before starting the experiment. Then, the eye-tracker was calibrated. To ensure that every participant watched the same content, the canvas was centered once they were in a comfortable position. Then, the participants were instructed to watch the animation after which a series of questions would follow. In the case of VR, they were informed that the main action would happen in front of them. During a pilot study we found that participants were more comfortable answering orally to the questions, so we recorded and faithfully transcribed the interview.

Participants watched the animation twice. During the first visualization, they were asked to explain what they had seen in the video. This question is extracted from the original work of Heider and Simmel^1^ (Question 1). For the second visualization, following previous work^1,20^, participants were encouraged to imagine that the shapes were acting as humans. Then, the animation was played again and they had to answer more detailed questions about their actions and personalities (Questions 2 to 11). The list of questions from this part of the experiment is as follows:

- **Question 1.** What happened in the animation?
- **Question 2.** What kind of a person is the big triangle?
- **Question 3.** What kind of a person is the small triangle?
- **Question 4.** What kind of a person is the circle?
- **Question 5.** Why did the two triangles fight?
- **Question 6.** Why did the circle go into the house?
- **Question 7.** In one part of the movie the big triangle and the circle were in the house together. What did the big triangle do then? Why?
- **Question 8.** What did the circle do when it was in the house with the big triangle? Why?
- **Question 9.** In one part of the movie the big triangle was shut up in the house and tried to get out. What did the small triangle and the circle do then?
- **Question 10.** Why did the big triangle break the house?
- **Question 11.** Tell the story of the movie.

With these questions the data collected consists of free-text answers, which are usually difficult to analyze quantitatively. For this reason, participants were asked to fill an additional questionnaire to obtain additional first-hand quantitative measurements. This questionnaire is adapted and simplified from the work of Greenberg and Strickland^15^, in which they replicate the Heider and Simmel experiment with a different measurement technique. Participants were asked to rate each shape depending on how they had perceived their behavior. For each shape, they had to select in a Likert-scale going from one to seven, how they agree in the following pairwise adjectives: *bad-good*, *foolish-wise*, *disreputable-reputable*, *passive-active*, *moderate-violent*, *slow-fast*, *fragile-tough*, *cowardly-brave* and *weak-strong*. The order of presentation (left-right position of the items and order of the shapes being evaluated) was randomized across participants.

Finally, to measure presence and engagement, participants were asked four questions: *How exciting was the scene?*^42^, *Watching the animated scene I had the feeling of “being there”*^43^, *I felt present in the virtual scene*^44^, and *To what extent were you aware of the real world around you while viewing the animation? (the sounds, the temperature of the room, other people, etc.)*^44^. These questions are included in the Igroup Presence Questionnaire (IPQ)^41^, which is usually taken as reference. Note that only a meaningful subset of questions has been used to avoid disengagement in the task and fatigue due to the length of the experiment. The overall duration of the experiment was about 30 minutes for both modalities (*screen* and *VR*).

### SAT index

In order to compute the seven indices that compose the SAT, the orally given answers are transcribed into text and then they are split into minimum meaningful units of information (*propositions*), which usually take the form of a verb + complement. Please see Sec. S3 in the supplementary for more details about the followed guidelines for labeling. Note that to compute the SAT index in the second impression, we use the answer to question 11 (tell the story of the movie).

Two trained *labelers* labeled the participants’ answers to compute the indices in order to ensure the reliability of the results. For quantifying the agreement between labelers, Intraclass Correlation Coefficient (ICC)^45,46^ is computed for all SAT indices. The mean ICC across SAT indices is 0.79 (considered excellent following the Cicchetti guidelines^47^) with a minimum of 0.58 (considered between fair and good). Please refer to Sec. S4 in the supplementary for all agreements. For the subsequent analysis, we use the mean values between the two labelers.

### Ethics statement

The experiments were conducted in accordance with the guidelines and regulations of Universidad de Zaragoza (Spain). Our experimental protocols comply with the requirements approved by the Comité de Ética de la Investigación de la Comunidad de Aragón (CEICA, Government Council). Written informed consent was obtained from participants before experiments began, and particular attention was paid to ensure that research data could be curated in an anonymized manner. At the outset of the experiment it was made clear to participants that they participated voluntarily and that they had the right to withdraw from the research at any time without giving a reason.

### Supplementary Information

Supplementary Information 1. Supplementary.

Supplementary Information 2. Gaze points video VR group first impression.

Supplementary Information 3. Gaze points video VR group second impression.

Supplementary Information 4. Gaze points video screen group first impression.

Supplementary Information 5. Gaze points video screen group second impression.

Supplementary Information 6. Saliency video VR group first impression.

Supplementary Information 7. Saliency video VR group second impression.

Supplementary Information 8. Saliency video screen group first impression.

Supplementary Information 9. Saliency video screen group second impression.

Supplementary Information 10. The Heider and Simmel animation as reproduced in our experiment.

## Data Availability

The data generated and analyzed in the present study are available at: https://graphics.unizar.es/projects/HeiderSimmelVR/.
