# Supplementary material for: Revisiting the Heider and Simmel experiment for social meaning attribution in virtual reality
Source: Sci Rep. 2024 Jul 24;14:17103. doi: 10.1038/s41598-024-65532-0 (PMC11269668; doi:10.1038/s41598-024-65532-0)
Supplement: Supplementary file 1 — Supplementary Information 1. Supplementary. [file 41598_2024_65532_MOESM1_ESM.pdf]

Supplementary Material:

Revisiting the Heider and Simmel experiment for social meaning attribution in virtual reality

The suplementary material is composed of:

- This document.
- The Heider and Simmel animation [2] as reproduced in our experiment.
- Videos with saliency and gaze points from participants overlayed on top of the animation for those who watched the experiment with the Head Mounted Display (HMD) and through the traditional display (screen) for the first impression and second impression. Additionally, we have also uploaded these videos in the following hidden playlist: `https://www.youtube.com/playlist?list=PLzb92_pAXzPUUOYsTXMVVBLw1Zu8ZpeIV`.

This document offers additional information and details about the following topics of the main paper:

- S1: Demographic questionnaire
- S2: Presence questionnaire
- S3: Computation of SAT indices
- S4: Agreement between labelers
- S5: Implementation details
- S6: Detailed statistical analyses and plots

S1 Demographic questionnaire

The following demographic questionnaire is fulfilled by the users after filling out the informed consent.

Demographic questionnaire

1. **Subject ID** ____
2. **I agree to participate in this research study. I understand the purpose and nature of this study and am participating voluntarily. I understand that I may withdraw from the study at any time without penalty or consequence. I consent to the use of the data generated from this questionnaire in the researcher's publications on this topic. Any information obtained in connection with this study that can be identified with you will remain confidential and will be released only with your permission.** □ I agree
3. **I authorize the recording of this session for further study.** □ I agree
4. **Age:** ________
5. **Genre**
   - □ Male
   - □ Female
   - □ Other: __________
6. **Do you play video games on a daily basis?**
   - □ Yes
   - □ No
7. **Do you watch series and/or movies frequently?**
   - □ Yes
   - □ No
8. **Do you have a vision problem?**
   - □ Yes
   - □ No
9. **If you have a vision problem, what type of problem is it (e.g. poor distance vision, etc.)?** ____

10. **If you have any vision problems, do you have them corrected? (e.g. wearing glasses or contact lenses)**
    - ☐ Yes, I am wearing glasses
    - ☐ Yes, I am wearing contact lenses
    - ☐ No
11. **Do you have any characteristics that make you fall into the neurodivergent group (e.g., dyslexia, autism, etc.)? If so, please indicate your condition.**

    ____

12. **Add any information you consider relevant.**

    ____

Virtual reality related questions

Now you are going to answer questions related to the use of virtual reality devices. Skip this part if you are not going to perform the experiment with a virtual reality device. If you do not know, ask the experimenter.

13. **Have you ever used a virtual reality device?**
    - ☐ Yes
    - ☐ No
14. **If you have used any virtual reality devices, how often?**
    - ☐ Low (about 5 times in total)
    - ☐ Moderate (occasionally)
    - ☐ High (in my daily life)
15. **If you have used it, check those that apply**
    - ☐ I have tested computer-type devices (HTV Vive, Oculus, PlayStation VR...)
    - ☐ I have tested devices that use a smartphone
    - ☐ I use virtual reality devices in my daily life
16. **Have you ever experienced eyestrain, sickness, headaches, or nausea when using virtual reality?**
    - ☐ Yes
    - ☐ No

S2 Presence questionnaire

The following questionnaire is fulfilled by the users once they have finished the experiment.

Presence questionnaire

17. How exciting was the animation?

not exciting at all □—□—□—□—□—□—□ very exciting

18. Watching the animated scene I had the feeling of "being there".

nothing at all □—□—□—□—□—□—□ a lot

19. I felt present in the virtual scene.

totally disagree □—□—□—□—□—□—□ totally agree

20. To what extent were you aware of the real world around you while viewing the animation? (the sounds, the temperature of the room, other people, etc.).

I was extremely aware of what was going on around me	□—□—□—□—□—□—□	I was completely unaware of what was going on around me

S3 Computation of SAT indices

The Social Attribution Task (SAT) indices have been computed following the guidelines indicated by Klin [3] and extended by Ratajska et al. [5]. However, the experimental conditions presented in this project are different with respect to the conditions presented in the work of Klin, so some indices computation has slightly varied to better fit into this project context. These differences are indicated in each index computation description.

S3.1 Transcription guidelines

Once the participant has finished the experiment, the answers given in the interview are manually transcribed by the instructor to facilitate the analysis process. For the transcription process several guidelines have been taken into account:

- Pauses where the participant is thinking are omitted, i.e., "*uhm...*", "*let me think...*".
- When synonyms without a meaningful value are used to refer to the main figures they are replaced by the big triangle, small triangle, and circle, accordingly. For example, a ball or sphere would be replaced by a circle. However, pronouns remain untouched.
- If the participant reports lots of information at once without any pauses, a punctuation mark is added accordingly.

S3.2 Pertinence Index

The goal of the metrics is to measure the capacity to make social attributions. For this reason, those propositions provided in the narratives that have nothing to do with social attributions are marked as non-pertinent propositions. This includes:

1. *vague references*. A proposition that does not contribute with meaningful information usually takes the form of describing a geometric event, e.g., some figures spinning around.
2. *irrelevant attributions*. A proposition that can be omitted and the plot of the movie can be equally understood, e.g., the triangle has made a strange pattern.

In this project, *misattributions* and *inconstant propositions* are not taken into account as non-pertinent propositions, unlike Klin's work. This is because they include social attribution even if they had nothing to do with the movie plot, so they are equally valid. For example, if a subject

says that the figures are playing tag, it is actually making a social attribution although it can be seen that the large triangle behaves violently with the small triangle.

Additionally to Klin's work, when marking propositions as pertinent, every proposition that includes a cognitive or affective term is considered as pertinent (see Sections S3.3 and S3.4 for the guidelines to identify these terms, respectively). A proposition can have one affective term and one cognitive term at the same time, but it cannot contain two or more terms of the same type. In that case, the proposition is split into two propositions.

Then, this index indicates the percentage of the propositions that are non-relevant and it is computed as the Equation Eq. 1 shows.

$$\begin{aligned} \text{Pertinence Index} = (&\text{Total number of propositions} - \\ &\text{Number of pertinent propositions}) \; / \\ &\text{Total number of propositions} \end{aligned} \qquad \text{(Eq. 1)}$$

S3.3 Cognition Index

This index corresponds to the percentage of pertinent propositions containing mental state terms (denoting desire, knowledge, thoughts, motivation, intention, or behaviors intended to alter another person's mental states) from the total number of pertinent propositions included in the participant's narratives. Cognitive mental states are defined as:

1. Terms expressing one character's desire for knowledge. Some examples include nosing around, finding, etc.

2. Behaviors which not only implicitly indicate a shared cognition, thought, or plan between two characters but which cannot exist without it. This includes terms such as trapping, protecting, taking cover, turning away, following, rescuing, sneaking, hiding, spying, collaborating, etc.

3. Terms expressing one character's belief, thought, imagination, plan, or motivation. Some examples include hitting, taking something away, breaking down the door, destroying, attempting, shouting, etc.

Note that in the original work it is also considered behaviors that explicitly indicate a shared cognition, thought, or plan between two characters in which one character intentionally impacts the other's cognitive state, e.g., bullying, arguing, joking, etc. In this case, these terms are

considered affective terms, since they have affective connotations which involve emotions. Additionally, comparisons that make an allusion to people's domains such as a dorm or a home or directly to people's roles such as the house owner, thief, man, woman, etc. are also considered as cognitive propositions.

The index is computed as the Equation Eq. 2 shows:

$$\text{Cognitive Index} = \frac{\text{Number of cognitive propositions}}{\text{Number of pertinent propositions}} \qquad \text{(Eq. 2)}$$

S3.4 Affective Index

This index corresponds to the percentage of propositions containing emotional terms, e.g, happy, sad, alarmed, etc. attributed to the characters of the video from the total number of pertinent propositions made. Affective mental states are defined as:

1. Emotional terms that may not be the result of social interaction or may not be uniquely human. Some examples include fooling around, defeated, not liking, being cooler, territorial, achieving something, playing, winner, etc.

2. Behaviors which not only implicitly indicate a shared emotional state between two characters but which cannot exist without it. Some examples include getting along with, celebratring, high-fiving, allying, being together, meeting, taking sides, recruiting someone, cheering, hugging, etc.

3. Emotional terms which result only from a social situation. Some examples include ruining something, being left alone, scared, screaming, calming, envious, jealous, sulking, admiration, etc.

4. Behaviors which explicitly indicate a shared cognition, thought, or plan between two characters in which one character intentionally impacts on the other's state. Some examples include attacking, fighting, combatting, beating someone, going for someone, cornering someone, facing someone, robbing, disturbing, arguing, kicking out, assaulting, ignoring, scolding someone, leaving alone, engaging, bullying, joking, etc.

The index is computed as the Equation Eq. 3 shows:

$$\text{Affective Index} = \frac{\text{Number of affective propositions}}{\text{Number of pertinent propositions}} \qquad \text{(Eq. 3)}$$

S3.5 Salience Index

The movie narrative can be interpreted in very different forms. However, there are salient items of social attribution that can be identified along with the narratives. The same 20 items that in Klin's experiment [3] are considered for this index computation:

1. Rectangle is a human enclosure.
2. Recognition of three actors (the rectangle is not an actor, three agents throughout).
3. Little triangle and circle are together (may be implicit).
4. The big triangle and the small triangle fight.
5. Indication of the direction of hostility: The big triangle is the aggressor, the little triangle is resistant.
6. The little triangle is overwhelmed by the big triangle (e.g., The big triangle wins, the big triangle scares off the little triangle).
7. The little circle tries to avoid conflict, e.g., hides, cowers, seeks protection.
8. The big triangle searches for the little circle, e.g., entraps, tries to catch.
9. The little circle panics, e.g., is afraid, scared, terrified.
10. Indication that the little triangle comes to the little circle's aid, e.g., save, rescue, help.
11. The little circle escapes the big triangle, e.g., evades, flees, gets away from.
12. The big triangle is trapped inside the enclosure.
13. The little circle and the little triangle celebrate, e.g., are happy, dance, rejoice.
14. Proposition explaining the reason for celebration (e.g., escaped from the big triangle, are free).
15. Indication that the big triangle chases the little triangle and the little circle, e.g., goes after, pursues them.
16. Indication that the big triangle momentarily does not know where the little triangle and circle are (as a result of the big triangle's momentary search of the two other shapes inside the rectangle).

17. The little triangle and the little circle are successful at evading the big triangle, e.g., they escape, ran away.

18. The big triangle is frustrated, e.g., mad, angry.

19. Proposition of explanation for the big triangle's anger (e.g., because he failed to catch them).

20. The big triangle breaks the enclosure.

The metric indicates the percentage of salient forms detected and it is computed following the Equation Eq. 4:

$$\text{Salience Index} = \frac{\text{Number of salient items detected}}{20} \quad \text{(Eq. 4)}$$

S3.6 Animation Index

This index corresponds to a summary measure of the narrative's general level of social attribution. It includes:

1. Behaviors (doing something):

   A. Behaviors that necessitate actors or agents, but which are not uniquely or necessarily human behaviors, nor do they necessarily require any attribution of mental or feeling states, i.e., chasing, fighting, destroying, dancing.

   B. Verbs or behaviors that do not involve an explicit mental state but are uniquely human, i.e., talking, saying, or a quotation.

   C. Behaviors that are uniquely human by virtue of implied indication of a shared mental state without which the behavior cannot occur, i.e., cheering, celebrating, trapping, hiding, escaping, running away, playing, helping.

   D. Behaviors that are uniquely human by virtue of direct indication of awareness by one character of another's mental state, accompanied by an attempt to alter the second character's mental state, i.e., intimidation, deception, trickery, bullying, arguing, joking, rebuffing, taunting, distract, making trouble.

2. Perceptions:

   E. Sensory experiences or attention which are not uniquely human, i.e., look, watch, see, hearing, notice.

3. Emotions (feeling something):

   F. Emotional terms that usually result from a behavior or an action, but which do not necessarily result from a social action, or which are not uniquely human, i.e., happy, sad, scared, mad, alarmed, panicked, nervous, annoyed.

   G. Emotional terms which result only from a social situation, i.e., envious, jealous, sulking, bitter, enraged.

4. Cognition, intention, motivation (usually thinking something):

   H. Lower developmental level: mental state terms expressing desire or knowledge, i.e., want to, know, ask for something, achieving something.

   I. Higher developmental level: mental state terms expressing beliefs, thoughts, imagination, plans, i.e., pretending, remembering, decision, robbing.

5. Relationships or personality traits:

   J. Allusion to a person as constrained by his or her features, i.e., big guy, little guy, kid.

   K. Allusion to a person as constrained by his or her relationship to another, i.e., is a daddy, mommy, or baby.

   L. Allusion to a person as constrained by his or her actions or attribution of personality traits, i.e., to be a bully, friends, companions, curious, timid, shy.

6. Symbolic nature:

   M. An acknowledgement of the symbolic nature of an object or shape, e.g., represents, stands for, symbolizes, a home, domain.

The index value goes from 0 to 6. A value of 0 means that the subject has interpreted the whole movie purely in geometric terms. The algorithm to compute the metric is indicated in Table S3.1:

S3.7 Person Index

As part of the experiment, participants are explicitly instructed to see the shapes as they behave like people, and then to answer the question of what kind of a person they were. It was intended to measure the participant's ability to derive invariant or stable personality features from the

Animation Index	Criteria
0	No human agency; mechanistic; geometric reasoning only
1	A or E or J
2	B or C or F or H or K or M
3	D or G or I or L
4	At least two of D or G or I or L, but not two of the same category
5	At least three of D or G or I or L, but not two of the same category
6	Four of D or G or I or L, but at least one of each

Table S3.1: Algorithm to compute the animation index.

shapes' actions in the video. This ability was graded in ascending level based on the participant's use of the characters.

1. *Physical properties (PP)*: descriptions based on the shapes' form, i.e., big, small, skinny.

2. *Relative properties (RP)*: descriptions of the interrelated social roles of the characters although still related to their relative shape, i.e., adult, dad, mother, grown-up, kid, boy, baby.

3. *Behaviorally derived attributes (BDA)*: descriptions based on specific actions of the characters, i.e., protector, trapping kind of person, scared, fighter, coward, naughty.

4. *Psychologically derived features (PDF)*: these attributions reflect characterological statements, i.e., features that the characters would carry with them beyond the specific events portrayed in the video, e.g., curious, timid, aggressive, bitter, wicked, cautious, brainless, quick-tempered, immature, toxic, lazy.

Like the animation index, this index is scored hierarchically, in terms of the level achieved, rather than the frequency of attributes generated for each character. Ratings are used to measure the level of person attribution on an ordinal scale of 0 to 6. The algorithm to compute the metric is indicated in Table S3.2:

S3.8 Problem-Solving Index

In the experiment, participants are instructed to answer specific questions after once they have watched a second time the animation. The index was intended to assess the participant's ability

Person Index	Criteria
0	Do not know, or all PPs
1	One or more RPs
2	At least 1 BDA
3	At least 1 BDA for each character or at least 1 PDF
4	At least 3 PDFs
5	At least 1 PDF for each character
6	Four or more PDFs (i.e., more than one PDF for each character)

Table S3.2: Algorithm to compute the person index.

to answer correctly explicit questions about the cartoon. The index score indicates the percentage of the items answered correctly. The index was also intended to measure the participant's ability to profit from the explicit verbal instruction to make social attributions describing a specific part from the animation story.

Question 5. Why did the two triangles fight?

Item 1: Recognition that the large triangle and the small triangle (and/or circle) had antagonistic intentions, motivations, or beliefs that put them at odds (i.e., "they disagree", "they think differently", "they have a conflict of interest", "the big triangle wanted to take something away from the little triangle").

Question 6. Why did the circle go into the house?

Item 2: Recognition that the small circle was trying to escape the conflict (i.e., "hiding", "seeking protection") (to say that it was scared is not sufficient).

Question 7. In one part of the movie, the big triangle and the circle were in the house together. What did the big triangle do then?

Item 3: Indication that the big triangle wanted to catch, entrap the small circle (an indication

that the big triangle "notices the circle" or "finds the circle" are not sufficient).

Question 8. What did the circle do when it was in the house with the big triangle? Why?

Item 4: Indication that the small circle panics, is afraid, etc.
Item 5: Indication that the small circle is trying to escape.

Question 9. In one part of the movie the big triangle was shut up in the house and tried to get out. What did the little triangle and the circle do then?

Item 6: The small triangle and circle celebrated, danced together because they were happy (it is important that the answer includes some form of celebration or happiness as a result of the event; simply saying that the two small shapes were playing together is not sufficient).
Item 7: An explanation of why the two shapes are happy, i.e., the small circle, which was in some peril, is now safe.

Question 10. Why did the big triangle break the house?

Item 8: The large triangle broke the house because it was angry, or a different negative (frustrating) emotion.
Item 9: Explanation for the large triangle's anger (i.e., it could not catch the other two shapes, or a different reason resulting from the story).

This index has one item less than the one from the work of Klin [3], which corresponds to a question that was not considered in this adapted version of the experiment. The metric is computed as the Equation Eq. 5 shows:

$$\text{Problem-Solving Index} = \frac{\text{Number of items answered correctly}}{9} \qquad \text{(Eq. 5)}$$

S3.9 Example of labeled responses

The original experiment was conducted in Spanish, the native language of the participants, to ensure language did not hinder their descriptive abilities. For clarity in this document, we

provide two authentic participant responses, translated from Spanish into English. Note that in Spanish, in many cases, the subject is not explicitly used, so in those omissions we translate it as *he/she* and *him/her* when the subject attributes characteristics of a human being but without specifying the gender.

Following the procedure detailed in the main document, once subjects have finished watching for the first time the animation, they answer Question 1, *What happened in the animation?* Then, this answer can be used to compute the first impression SAT indices. First, the pertinence, cognition and affective indices are computed. Each proposition is enclosed by parentheses and it has associated a superscript indicating if it contains a cognitive term (CT) or affective term (AT), if applicable. Note that a proposition is pertinent if contains either a cognitive or affective term.

Subject 1. *(The big triangle entered the rectangle.) (To me it was as if he/she was at home.)^{CT} (Then a circle and a small triangle appeared.) (Somehow they wanted to enter the house.)^{CT} (At first he/she seemed like a struggle.)^{AT} (The big triangle didn't get along well with the small triangle,)^{AT} (and the circle was there kind of going unnoticed,)^{CT} (and then sneaking into the house.)^{CT} (He was like a gentleman who from the perspective of the big triangle,) (was a nuisance,)^{AT} (but for the other two it was more like a game.)^{CT} (They were collaborating to get into the house.)$^{CT,\,AT}$ (The big triangle is like a grandfather,) (and then there are the two kids causing trouble.)^{AT} (The small triangle was distracting the big triangle,)^{CT} (and the circle was entering the house,)^{CT} (exploring it.)^{CT} (Later when he realized,)^{CT} (he came out,) (and there was a bit of a chase around the house.)^{CT} (In the end the big triangle destroyed the house.)^{CT} (He somehow destroyed his surroundings.)^{CT}*
Total number of propositions = 22
Number of cognitive propositions = 13
Number of affective propositions = 5
Number of pertinent propositions = 17
Pertinence index = $\frac{22-17}{17} \approx 0.22$
Cognition index = $\frac{13}{17} \approx 0.76$
Affective index = $\frac{5}{17} \approx 0.29$

Subject 2. *(There was a box.) (Let's say it's a room,)^{CT} (because it seemed to have a door.)^{CT} (There was a big triangle.) (The big triangle at first I think entered the box,) (and must have come out.) (Then a small triangle and a circle appeared,) (and it seemed like there was a fight among them.)^{AT} (I believe the circle participated the least in the fight.)^{AT} (It seemed more*

like a conflict between the two triangles that were battling each other.)^{AT} (There was a lot of movement with people entering,)^{CT} (and leaving the box.)^{CT} (There was also people colliding with each other,)^{CT} (both with the box and the triangles among themselves.) (It seemed like the small triangle and the circle were together,)^{AT} (fleeing from the big triangle.)^{CT} (The big triangle broke the door of the house,)^{CT} (and entered inside.)
Total number of propositions = 18
Number of cognitive propositions = 7
Number of affective propositions = 4
Number of pertinent propositions = 11
Pertinence index = $\frac{18-11}{18} \approx 0.39$
Cognition index = $\frac{7}{11} \approx 0.64$
Affective index = $\frac{4}{11} \approx 0.36$

Regarding to the salience index, Subject 1 notice the items 1, 2, 3, 4, 7, 11, 15, 20, as indicated in Section S3.5, then salience index $= \frac{8}{20} = 0.40$. Subject 2 notice the items 1, 3, 4, 7, 15, 20, then salience index $= \frac{6}{20} = 0.30$.

Concerning the animation index, Subject 1 meets A, B, C, D, E, G, H, I, J, as indicated in Section S3.6, then the animation index is 5. Subject 2 meets A, C, M, then then the animation index is 2.

Then, the subjects watch the animation again and they are asked to answer the questions 2, 3, and 4 indicated in the main document, which are related to the kind of person of each figure. These answers allow to compute the person index. We report the answers of the two subjects, indicating with a superscript if the terms are physical properties (PP), relative properties (RP), behaviorally derived attributes (BDA), or phsychologically derived features (PDF).

Question 2. What kind of a person is the big triangle?

Subject 1. *A person with a bit of a (bad temper)^{PDF}. A (grumpy person)^{PDF}.*
Subject 2. *The large triangle is a very (aggressive person)^{PDF}. From what I've observed, it seems like the small triangle, who appears to be his (child)^{RP}, and the circle, a (friend)^{RP}, approached. What I've interpreted is that the large triangle didn't like the circle, perhaps disapproving of his child bringing a friend. So, (it became very violent because he doesn't tolerate the circle or doesn't like circles)^{BDA}.*

Question 3. What kind of a person is the small triangle?

Subject 1. *A bit (cheeky)^{PDF}. A (troublemaker)^{PDF}. A (nuisance)^{PDF}.*

Subject 2. *More (tolerant)*^{PDF}. *(It brought the circle along)*^{BDA}. *He/She appears as a (friendly person)*^{PDF} *who (protects its friends)*^{BDA}. *He/She was seen with (good intentions)*^{BDA} *and (tried to help the circle)*^{BDA} *escape. (He/She comes across as a kinder person)*^{PDF}.

Question 4. What kind of a person is the circle?

Subject 1. *Someone more (cautious)*^{PDF}. *(He/She seems a bit more fearful)*^{BDA}. *(He/She dislikes confrontation)*^{BDA}. *When the two triangles argue, (the circle tends to stay more on the sidelines)*^{BDA}*, observing.*

Subject 2. *More (timid)*^{PDF} *because (he/she didn't interact much with people)*^{BDA}. *People simply interacted with him/her. Generally (shy)*^{PDF}.

According to the guidelines established in Section S3.7 both subjects achieve a person index of 6.

Then the subjects continue answering questions 5 to 10, used to compute the problem-solving index.

Question 5. Why did the two triangles fight?

Subject 1. *I suppose the small triangle has a more provocative and noisy attitude, provoking the large triangle. Perhaps the large triangle doesn't want anyone around his/her environment, and then they appear in his/her garden. The large triangle is like, "Get out of my garden!" and the small one responds, "No!" They fight over something like that, let's say.*

Subject 2. *From what I've interpreted, it seems like the large triangle is the father. The small triangle brought a friend that the large triangle didn't like. Perhaps it's because the circle belongs to a different race, maybe it's something the large triangle doesn't approve of. It's as if the large triangle is saying to its child, "What are you doing bringing these friends?"*

Question 6. Why did the circle go into the house?

Subject 1. *The circle is scared when the large and small triangles start fighting. He/She seeks a place to feel more protected.*

Subject 2. *To protect himself/herself because the large and small triangles were fighting outside the house. It's the only safe place. He/She could escape, but initially, he/she seemed to hide behind the door and then entered to avoid the two triangles.*

Question 7. In one part of the movie the big triangle and the circle were in the house together. What did the big triangle do then? Why?

Subject 1. *It seems like the large triangle is blocking the door, the exit. So, I get the impression that the circle tries to run, distract him/her to be able to leave, to escape from the house.*

Subject 2. *The large triangle approaches the circle stealthily. I imagine it's because the circle*

represents a race or a form that the large triangle doesn't like, and he/she slowly approaches. Then I think they have a small fight. It's not as intense as the large triangle versus the small triangle, but there's movement between the two. The circle seemed a bit agitated. It's more of a disagreement or discussion between them, but without violence.

Question 8. What did the circle do when it was in the house with the big triangle? Why?

Subject 1. *He/She is waiting to see what the big triangle does. He/She is afraid of the big triangle. He/She is on the lookout to try to get out. He/She stays still and then starts moving very quickly. He/She is trying to confuse the big triangle to escape. I think he/she is afraid of the big triangle.*

Subject 2. *The circle pleaded with the large triangle, trying to explain that being a circle isn't so bad.*

Question 9. In one part of the movie the big triangle was shut up in the house and tried to get out. What did the small triangle and the circle do then?

Subject 1. *They circled around the house.*

Subject 2. *They were circling around the house, well, that's later. Honestly, I don't remember.*

Question 10. Why did the big triangle break the house?

Subject 1. *Because he/she is angry. He/She has a bad temper.*

Subject 2. *Because, in the end, the small triangle and the circle fled. I'm not sure. It seems like the large triangle wanted more violence with them or something. They fled, and due to the rejection of the situation, frustration led him/her to break the house door.*

According to the guidelines specified in Section S3.8, Subject 1 satisfy the items 1, 2, 3, 4, 5, 8. Then problem-index $= \frac{6}{9} \approx 0.67$. Subject 2 satisfy the items 1, 2, 4, 8, 9. Then problem-index $= \frac{5}{9} \approx 0.56$.

Finally, the subjects answer to question 11, *tell the story of the movie*. The answer is analyzed in the same way as the answer of question 1 previously analyzed.

Subject 1. *(The big triangle is a grumpy old man who is alone.)^{AT} (It reminds me a bit of a movie.) (The grandfather is at his house,)^{CT} (checking his lawn,)^{CT} (his fence or whatever it is.) (Then these two youngsters arrive,)^{CT} (and the small triangle goes into the lawn.)^{CT} (Somehow he is dragging the circle,)^{CT} (and the grandfather sees them,)^{CT} (calls their attention.)^{CT} (There's a commotion.)^{AT} (The circle gets scared,)^{AT} (tries to hide,)^{CT} (goes into the house.)^{CT} (Then the big triangle sees it,)^{CT} (and gets angry.)^{AT} (The big triangle enters the house.) (Afterward the small triangle and the circle escape,)^{CT} (and there's a bit of a fight before they leave.)^{AT} (Then*

the grandfather gets upset,)^{AT} *(and starts destroying everything.)*^{CT}

Total number of propositions = 21

Number of cognitive propositions = 12

Number of affective propositions = 6

Number of pertinent propositions = 18

Pertinence index = $\frac{21-18}{21} \approx 0.14$

Cognition index = $\frac{12}{18} \approx 0.67$

Affective index = $\frac{6}{18} \approx 0.33$

Subject 2. *(A small triangle befriends a circle,)*^{AT} *(and takes him/her to meet his father.)*^{CT} *(The small triangle introduces the circle to the big triangle,)*^{CT} *(who is not pleased at all that his son brought a circle,)*^{AT} *(as he's not fond of circles.)*^{AT} *(Initially he confronts his son.)*^{AT} *(The circle hides.)*^{CT} *(The big triangle enters the house to confront the circle.)*$^{CT, AT}$ *(In the end the circle and the small triangle manage to escape.)*^{CT} *(The big triangle releases his frustration,)*^{AT} *(by breaking the door and letting off steam with the house.)*^{AT}

Total number of propositions = 11

Number of cognitive propositions = 5

Number of affective propositions = 7

Number of pertinent propositions = 11

Pertinence index = $\frac{11-11}{11} = 0$

Cognition index = $\frac{5}{11} \approx 0.45$

Affective index = $\frac{7}{11} \approx 0.64$

Regarding to the salience index, Subject 1 notice the items 1, 2, 3, 4, 7, 8, 11, 17, 18, 19, 20, then salience index $= \frac{11}{20} = 0.55$. Subject 2 notice the items 1, 2, 3, 4, 5, 7, 8, 17, 18, 19, 20, then salience index $= \frac{11}{20} = 0.55$.

Concerning the animation index, Subject 1 meets A, B, D, E, G, H, J, L, M, then the animation index is 5. Subject 2 meets A, B, C, D, G, I, J, K, M, then the animation index is 5.

S4 Agreement between labelers

Given the subjectivity of the indices' computation, two labelers have been needed to compute them. Each of the labelers computed by their own index for each subject and, in the end, intraclass correlation coefficient (ICC) was performed across all the indices. Following the Fleiss convention [6], we follow an ICC(3,k) because the two raters are fixed and each rater is going to evaluate each subject. Additionally, the final metric per subject will be averaged among raters and we ensure consistency agreement [4]. The mean ICC across SAT indices is 0.79 (considered excellent following the Cicchetti guidelines [1]) with a minimum of 0.58 (considered between fair and good). The agreement per each SAT index can be observed in Table S4.3. The computation has been done with the Pingouin statistical package for Python [1].

SAT Index	Watching time	Type	Description	ICC	F	df1	df2	pval	CI95%
Pertinence Index	1st	ICC(3,k)	Average fixed raters	0.82	5.50	51	51	4.69e-09	[0.68, 0.9]
	2nd	ICC(3,k)	Average fixed raters	0.81	5.20	51	51	1.26e-08	[0.67, 0.89]
Salience Index	1st	ICC(3,k)	Average fixed raters	0.86	6.99	51	51	5.18e-11	[0.75, 0.92]
	2nd	ICC(3,k)	Average fixed raters	0.91	10.94	51	51	5.48e-15	[0.84, 0.95]
Cognition Index	1st	ICC(3,k)	Average fixed raters	0.80	4.95	51	51	3.04e-08	[0.65, 0.88]
	2nd	ICC(3,k)	Average fixed raters	0.58	2.38	51	51	0.001	[0.27, 0.76]
Affective Index	1st	ICC(3,k)	Average fixed raters	0.80	5.10	51	51	1.81e-08	[0.66, 0.89]
	2nd	ICC(3,k)	Average fixed raters	0.70	3.36	51	51	1.4e-5	[0.48, 0.83]
Animation Index	1st	ICC(3,k)	Average fixed raters	0.80	5.12	51	51	1.64e-08	[0.66, 0.89]
	2nd	ICC(3,k)	Average fixed raters	0.74	3.91	51	51	1.48e-06	[0.55, 0.85]
Person Index	2nd	ICC(3,k)	Average fixed raters	0.88	8.53	51	51	9.82e-13	[0.8, 0.93]
Problem-Solving Index	2nd	ICC(3,k)	Average fixed raters	0.81	5.14	51	51	1.53e-08	[0.66, 0.89]

Table S4.3: Agreement between labelers per each SAT Index for the first and the second impression.

[1] `https://pingouin-stats.org/index.html`

S5 Implementation details

This section presents the experiment's implementation details regarding how the animation is coded, the chinrest placement, and how the physiological measurements are computed.

S5.1 Animation implementation

In order to have comparable metrics between both versions of the experiment (*screen* and *VR* groups), some aspects regarding the visualization should be considered. The main difference is that for the *screen* version the original condition of the experiment is preserved, i.e., figures are two-dimensional. To achieve this, the experiment is developed as three-dimensional but an orthographic instead of a perspective camera is used, generating the illusion of a two-dimensional video.

To take into account visual errors when detecting which figures are the subject watching, a sphere is projected from the screen to the Unity world of a pre-defined radius. On the other hand, for the *VR* it is projected from the camera (subjects' eye) to the Unity world with a pre-defined radius. The radius of the projected sphere is computed taking into account the visual error on the scene plane, which remains the whole time over the same axes. It would be more correct to project a cone but projecting a sphere is a correct approximation since figures do not move in the depth axis.

S5.2 Chinrest placement

To ensure that both, *screen* and *VR* participants groups, observe the stimulus in the same visual angle some computations have to be done. To do so, the bottom wall of the stimulus' *house* is taken as a reference. In this case, this wall measures 10 meters (a unit in Unity resembles a meter in the real world), and it is seen from 49.5 meters. Then, the visual angle β should be the same in the *screen* situation, α. This situation is represented in Figure S5.1. The same visual angle is ensured with the equation Eq. 6.

$$tan(\alpha) = \frac{x}{y} = \frac{x'}{y'} = tan(\beta) \qquad \text{(Eq. 6)}$$

In this case, x is fixed to 0.117 m, so clearing y, it remains:

$$y = \frac{x * y'}{x'} = \frac{0.117 * 49.5}{10} = \frac{5.8}{10} = 0.58m \qquad \text{(Eq. 7)}$$

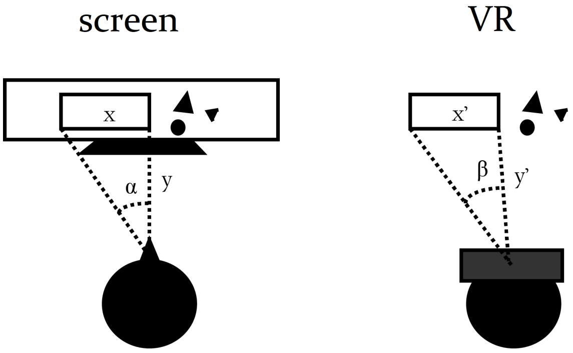

Figure S5.1: Geometric relations to place the chinrest in order to get the animation to cover the same visual angle in the *screen* and *VR* conditions.

Then, we know that the chinrest should be placed at 0.58m from the screen to guarantee that the visual angle covers the same elements in the *screen* and *VR* groups.

S6 Additional results

In this section, we present additional plots and results for the various computed metrics reported in the main document.

S6.1 Mean, standard deviation and normality tests

In this subsection, we provide the means and standard deviations for each of the variables analyzed in the main document. Additionally, we conduct an analysis to assess the normality of the variables for each of the groups, *VR* and *screen*, using the Shapiro-Wilk Test. The results are presented in Tables S6.4 to S6.8.

S6.2 SAT indices statistical analyses

In this subsection, comprehensive statistical tables detailing the analyses conducted in the main document concerning the SAT indices are presented. Results are shown in Tables S6.9 to S6.11.

S6.3 Greenberg-Strickland questionnaire analyses

In this subsection, a complete statistical table of the analyses pertaining to the ratings of the Greenberg-Strickland questionnaire (evaluative, activity, potency) is provided. Results are shown in Table S6.12. In the main document, we stated that we computed the two-way interaction between the shape (*big triangle*, *small triangle*, *circle*) and the modality (*VR*, *screen*) as well as the three-way interaction considering the clusters (evaluative, activity, potency). To do so, we computed the differences between the rating of one shape with the other shapes from a cluster, and then computed a Mann-Whitney U-Test to contrast differences between the *VR* and *screen* groups. The three-way interaction is computed in the same way, but computing the differences of the previous already computed differences among clusters. Results are shown in Table S6.13.

S6.4 Objective analyses

S6.4.1 Global AUC

In this section we report the global AUC metric full statistical results. They are shown in Table S6.14.

	Mean	*SD*	Shapiro-Wilk Test
No. of propositions 1st	$VR = (\mu = 19.038)$<br>$screen = (\mu = 21.333)$	$VR = (\sigma = 5.41)$<br>$screen = (\sigma = 8.0)$	$VR = (W(26) = 0.972, p = 0.678)$<br>$screen = (W(27) = 0.971, p = 0.633)$
No. of propositions 2nd	$VR = (\mu = 22.577)$<br>$screen = (\mu = 21.692)$	$VR = (\sigma = 11.025)$<br>$screen = (\sigma = 8.004)$	$VR = (W(26) = 0.84, p < 0.001)^*$<br>$screen = (W(26) = 0.984, p = 0.946)$
Pertinence Index 1st	$VR = (\mu = 46.47)$<br>$screen = (\mu = 54.724)$	$VR = (\sigma = 17.381)$<br>$screen = (\sigma = 16.019)$	$VR = (W(26) = 0.929, p = 0.072)$<br>$screen = (W(27) = 0.94, p = 0.125)$
Pertinence Index 2nd	$VR = (\mu = 23.253)$<br>$screen = (\mu = 26.218)$	$VR = (\sigma = 11.619)$<br>$screen = (\sigma = 13.031)$	$VR = (W(26) = 0.929, p = 0.072)$<br>$screen = (W(26) = 0.956, p = 0.322)$
Salience Index 1st	$VR = (\mu = 39.808)$<br>$screen = (\mu = 38.333)$	$VR = (\sigma = 13.639)$<br>$screen = (\sigma = 14.938)$	$VR = (W(26) = 0.963, p = 0.453)$<br>$screen = (W(27) = 0.965, p = 0.468)$
Salience Index 2nd	$VR = (\mu = 53.462)$<br>$screen = (\mu = 50.481)$	$VR = (\sigma = 12.011)$<br>$screen = (\sigma = 13.463)$	$VR = (W(26) = 0.945, p = 0.179)$<br>$screen = (W(26) = 0.97, p = 0.618)$
Cognition Index 1st	$VR = (\mu = 77.807)$<br>$screen = (\mu = 73.082)$	$VR = (\sigma = 13.794)$<br>$screen = (\sigma = 18.814)$	$VR = (W(26) = 0.956, p = 0.325)$<br>$screen = (W(27) = 0.909, p = 0.022)^*$
Cognition Index 2nd	$VR = (\mu = 62.56)$<br>$screen = (\mu = 71.272)$	$VR = (\sigma = 9.055)$<br>$screen = (\sigma = 12.938)$	$VR = (W(26) = 0.968, p = 0.574)$<br>$screen = (W(26) = 0.983, p = 0.926)$
Affective Index 1st	$VR = (\mu = 23.272)$<br>$screen = (\mu = 27.915)$	$VR = (\sigma = 13.774)$<br>$screen = (\sigma = 18.479)$	$VR = (W(26) = 0.962, p = 0.431)$<br>$screen = (W(27) = 0.918, p = 0.035)^*$
Affective Index 2nd	$VR = (\mu = 39.916)$<br>$screen = (\mu = 30.727)$	$VR = (\sigma = 9.621)$<br>$screen = (\sigma = 13.655)$	$VR = (W(26) = 0.978, p = 0.831)$<br>$screen = (W(26) = 0.984, p = 0.949)$
Animation Index 1st	$VR = (\mu = 2.288)$<br>$screen = (\mu = 2.222)$	$VR = (\sigma = 0.868)$<br>$screen = (\sigma = 0.809)$	$VR = (W(26) = 0.918, p = 0.041)^*$<br>$screen = (W(27) = 0.93, p = 0.071)$
Animation Index 2nd	$VR = (\mu = 4.154)$<br>$screen = (\mu = 3.462)$	$VR = (\sigma = 0.907)$<br>$screen = (\sigma = 1.055)$	$VR = (W(26) = 0.913, p = 0.03)^*$<br>$screen = (W(26) = 0.934, p = 0.097)$
Person Index	$VR = (\mu = 3.173)$<br>$screen = (\mu = 3.574)$	$VR = (\sigma = 1.581)$<br>$screen = (\sigma = 1.682)$	$VR = (W(26) = 0.919, p = 0.043)^*$<br>$screen = (W(27) = 0.908, p = 0.02)^*$
Problem-Solving Index	$VR = (\mu = 53.419)$<br>$screen = (\mu = 49.588)$	$VR = (\sigma = 14.044)$<br>$screen = (\sigma = 10.468)$	$VR = (W(26) = 0.975, p = 0.76)$<br>$screen = (W(27) = 0.947, p = 0.183)$

Table S6.4: Mean, standard deviation (*SD*) and Shapiro-Wilk Test for the SAT indices for the first (1st), and second (2nd) impression. Asterisks (*) indicate departure from normality ($p < 0.05$).

	Mean	*SD*	Shapiro-Wilk Test
Evaluative T	$VR = (\mu = 2.615)$<br>$screen = (\mu = 2.938)$	$VR = (\sigma = 1.061)$<br>$screen = (\sigma = 1.391)$	$VR = (W(26) = 0.897, p = 0.014)^{*}$<br>$screen = (W(27) = 0.88, p = 0.005)^{*}$
Evaluative t	$VR = (\mu = 3.962)$<br>$screen = (\mu = 4.383)$	$VR = (\sigma = 1.406)$<br>$screen = (\sigma = 1.203)$	$VR = (W(26) = 0.93, p = 0.076)$<br>$screen = (W(27) = 0.97, p = 0.611)$
Evaluative c	$VR = (\mu = 5.167)$<br>$screen = (\mu = 4.963)$	$VR = (\sigma = 1.245)$<br>$screen = (\sigma = 0.991)$	$VR = (W(26) = 0.945, p = 0.18)$<br>$screen = (W(27) = 0.967, p = 0.513)$
Activity T	$VR = (\mu = 5.679)$<br>$screen = (\mu = 5.605)$	$VR = (\sigma = 0.67)$<br>$screen = (\sigma = 0.743)$	$VR = (W(26) = 0.967, p = 0.552)$<br>$screen = (W(27) = 0.961, p = 0.386)$
Activity t	$VR = (\mu = 5.679)$<br>$screen = (\mu = 5.333)$	$VR = (\sigma = 1.048)$<br>$screen = (\sigma = 1.054)$	$VR = (W(26) = 0.806, p < 0.001)^{*}$<br>$screen = (W(27) = 0.891, p = 0.008)^{*}$
Activity c	$VR = (\mu = 3.256)$<br>$screen = (\mu = 3.296)$	$VR = (\sigma = 1.006)$<br>$screen = (\sigma = 1.105)$	$VR = (W(26) = 0.879, p = 0.006)^{*}$<br>$screen = (W(27) = 0.952, p = 0.24)$
Potency T	$VR = (\mu = 5.846)$<br>$screen = (\mu = 5.753)$	$VR = (\sigma = 0.723)$<br>$screen = (\sigma = 0.712)$	$VR = (W(26) = 0.939, p = 0.125)$<br>$screen = (W(27) = 0.969, p = 0.578)$
Potency t	$VR = (\mu = 5.038)$<br>$screen = (\mu = 4.901)$	$VR = (\sigma = 1.279)$<br>$screen = (\sigma = 1.14)$	$VR = (W(26) = 0.959, p = 0.37)$<br>$screen = (W(27) = 0.878, p = 0.004)^{*}$
Potency c	$VR = (\mu = 2.769)$<br>$screen = (\mu = 2.531)$	$VR = (\sigma = 1.377)$<br>$screen = (\sigma = 0.833)$	$VR = (W(26) = 0.91, p = 0.026)^{*}$<br>$screen = (W(27) = 0.933, p = 0.083)$

Table S6.5: Mean, standard deviation (*SD*) and Shapiro-Wilk Test for the Greenberg-Strickland questionnaire. Asterisks (*) indicate departure from normality ($p < 0.05$).

	Mean	*SD*	Shapiro-Wilk Test
Global AUC 1st	$VR = (\mu = 53.194)$ $screen = (\mu = 65.201)$	$VR = (\sigma = 21.653)$ $screen = (\sigma = 13.187)$	$VR = (W(26) = 0.98, p = 0.871)$ $screen = (W(26) = 0.942, p = 0.154)$
Global AUC 2nd	$VR = (\mu = 59.172)$ $screen = (\mu = 71.797)$	$VR = (\sigma = 23.353)$ $screen = (\sigma = 23.582)$	$VR = (W(26) = 0.898, p = 0.014)^*$ $screen = (W(26) = 0.735, p < 0.001)^*$
Figure saliency T 1st	$VR = (\mu = 0.605)$ $screen = (\mu = 0.594)$	$VR = (\sigma = 0.068)$ $screen = (\sigma = 0.042)$	$VR = (W(26) = 0.963, p = 0.451)$ $screen = (W(26) = 0.934, p = 0.097)$
Figure saliency t 1st	$VR = (\mu = 0.248)$ $screen = (\mu = 0.264)$	$VR = (\sigma = 0.054)$ $screen = (\sigma = 0.043)$	$VR = (W(26) = 0.947, p = 0.195)$ $screen = (W(26) = 0.973, p = 0.706)$
Figure saliency c 1st	$VR = (\mu = 0.226)$ $screen = (\mu = 0.244)$	$VR = (\sigma = 0.046)$ $screen = (\sigma = 0.046)$	$VR = (W(26) = 0.957, p = 0.34)$ $screen = (W(26) = 0.89, p = 0.009)^*$
Figure saliency T 2nd	$VR = (\mu = 0.634)$ $screen = (\mu = 0.603)$	$VR = (\sigma = 0.09)$ $screen = (\sigma = 0.06)$	$VR = (W(26) = 0.974, p = 0.717)$ $screen = (W(26) = 0.964, p = 0.487)$
Figure saliency t 2nd	$VR = (\mu = 0.217)$ $screen = (\mu = 0.241)$	$VR = (\sigma = 0.076)$ $screen = (\sigma = 0.069)$	$VR = (W(26) = 0.973, p = 0.702)$ $screen = (W(26) = 0.948, p = 0.209)$
Figure saliency c 2nd	$VR = (\mu = 0.216)$ $screen = (\mu = 0.252)$	$VR = (\sigma = 0.055)$ $screen = (\sigma = 0.059)$	$VR = (W(26) = 0.989, p = 0.99)$ $screen = (W(26) = 0.884, p = 0.007)^*$
Number of figure changes 1st	$VR = (\mu = 88.808)$ $screen = (\mu = 112.538)$	$VR = (\sigma = 24.712)$ $screen = (\sigma = 23.67)$	$VR = (W(26) = 0.935, p = 0.1)$ $screen = (W(26) = 0.951, p = 0.251)$
Number of figure changes 2nd	$VR = (\mu = 82.769)$ $screen = (\mu = 98.731)$	$VR = (\sigma = 40.127)$ $screen = (\sigma = 35.59)$	$VR = (W(26) = 0.937, p = 0.114)$ $screen = (W(26) = 0.874, p = 0.004)^*$

Table S6.6: Mean, standard deviation (*SD*) and Shapiro-Wilk Test for the objective metrics for the first (1st), and second (2nd) impression. Asterisks (*) indicate departure from normality ($p < 0.05$).

	Mean	*SD*	Shapiro-Wilk Test
Number of figure changes 1st - Scene 1	$VR = (\mu = 1.692)$<br>$screen = (\mu = 0.0)$	$VR = (\sigma = 1.792)$<br>$screen = (\sigma = 0.0)$	$VR = (W(26) = 0.832, p < 0.001)^*$<br>$screen = (W(26) = 1.0, p = 1.0)$
Number of figure changes 1st - Scene 2	$VR = (\mu = 8.577)$<br>$screen = (\mu = 7.5)$	$VR = (\sigma = 5.024)$<br>$screen = (\sigma = 3.629)$	$VR = (W(26) = 0.957, p = 0.33)$<br>$screen = (W(26) = 0.892, p = 0.011)^*$
Number of figure changes 1st - Scene 3	$VR = (\mu = 6.308)$<br>$screen = (\mu = 7.577)$	$VR = (\sigma = 2.197)$<br>$screen = (\sigma = 3.053)$	$VR = (W(26) = 0.949, p = 0.22)$<br>$screen = (W(26) = 0.92, p = 0.045)^*$
Number of figure changes 1st - Scene 4	$VR = (\mu = 31.962)$<br>$screen = (\mu = 40.923)$	$VR = (\sigma = 12.62)$<br>$screen = (\sigma = 10.887)$	$VR = (W(26) = 0.974, p = 0.732)$<br>$screen = (W(26) = 0.935, p = 0.102)$
Number of figure changes 1st - Scene 5	$VR = (\mu = 6.423)$<br>$screen = (\mu = 8.692)$	$VR = (\sigma = 3.342)$<br>$screen = (\sigma = 3.098)$	$VR = (W(26) = 0.926, p = 0.062)$<br>$screen = (W(26) = 0.963, p = 0.464)$
Number of figure changes 1st - Scene 6	$VR = (\mu = 8.731)$<br>$screen = (\mu = 11.269)$	$VR = (\sigma = 4.752)$<br>$screen = (\sigma = 2.822)$	$VR = (W(26) = 0.953, p = 0.266)$<br>$screen = (W(26) = 0.97, p = 0.632)$
Number of figure changes 1st - Scene 7	$VR = (\mu = 6.692)$<br>$screen = (\mu = 8.731)$	$VR = (\sigma = 3.729)$<br>$screen = (\sigma = 2.58)$	$VR = (W(26) = 0.89, p = 0.009)^*$<br>$screen = (W(26) = 0.969, p = 0.603)$
Number of figure changes 1st - Scene 8	$VR = (\mu = 4.538)$<br>$screen = (\mu = 6.385)$	$VR = (\sigma = 3.354)$<br>$screen = (\sigma = 2.558)$	$VR = (W(26) = 0.927, p = 0.067)$<br>$screen = (W(26) = 0.963, p = 0.456)$
Number of figure changes 1st - Scene 9	$VR = (\mu = 3.077)$<br>$screen = (\mu = 3.846)$	$VR = (\sigma = 1.979)$<br>$screen = (\sigma = 1.634)$	$VR = (W(26) = 0.949, p = 0.221)$<br>$screen = (W(26) = 0.923, p = 0.053)$
Number of figure changes 1st - Scene 10	$VR = (\mu = 7.577)$<br>$screen = (\mu = 11.231)$	$VR = (\sigma = 3.353)$<br>$screen = (\sigma = 3.598)$	$VR = (W(26) = 0.893, p = 0.011)^*$<br>$screen = (W(26) = 0.953, p = 0.269)$
Number of figure changes 1st - Scene 11	$VR = (\mu = 1.346)$<br>$screen = (\mu = 2.885)$	$VR = (\sigma = 1.44)$<br>$screen = (\sigma = 1.625)$	$VR = (W(26) = 0.825, p < 0.001)^*$<br>$screen = (W(26) = 0.926, p = 0.063)$
Number of figure changes 1st - Scene 12	$VR = (\mu = 1.885)$<br>$screen = (\mu = 3.5)$	$VR = (\sigma = 1.908)$<br>$screen = (\sigma = 2.406)$	$VR = (W(26) = 0.852, p = 0.002)^*$<br>$screen = (W(26) = 0.871, p = 0.004)^*$
Number of figure changes 2nd - Scene 1	$VR = (\mu = 0.923)$<br>$screen = (\mu = 0.0)$	$VR = (\sigma = 1.412)$<br>$screen = (\sigma = 0.0)$	$VR = (W(26) = 0.693, p < 0.001)^*$<br>$screen = (W(26) = 1.0, p = 1.0)$
Number of figure changes 2nd - Scene 2	$VR = (\mu = 7.0)$<br>$screen = (\mu = 7.808)$	$VR = (\sigma = 5.084)$<br>$screen = (\sigma = 3.34)$	$VR = (W(26) = 0.937, p = 0.117)$<br>$screen = (W(26) = 0.967, p = 0.546)$
Number of figure changes 2nd - Scene 3	$VR = (\mu = 6.423)$<br>$screen = (\mu = 7.962)$	$VR = (\sigma = 5.839)$<br>$screen = (\sigma = 2.968)$	$VR = (W(26) = 0.76, p < 0.001)^*$<br>$screen = (W(26) = 0.954, p = 0.293)$
Number of figure changes 2nd - Scene 4	$VR = (\mu = 30.615)$<br>$screen = (\mu = 36.962)$	$VR = (\sigma = 19.093)$<br>$screen = (\sigma = 14.171)$	$VR = (W(26) = 0.955, p = 0.299)$<br>$screen = (W(26) = 0.845, p = 0.001)^*$
Number of figure changes 2nd - Scene 5	$VR = (\mu = 6.038)$<br>$screen = (\mu = 6.154)$	$VR = (\sigma = 4.155)$<br>$screen = (\sigma = 3.022)$	$VR = (W(26) = 0.917, p = 0.038)^*$<br>$screen = (W(26) = 0.949, p = 0.219)$
Number of figure changes 2nd - Scene 6	$VR = (\mu = 8.346)$<br>$screen = (\mu = 9.462)$	$VR = (\sigma = 4.169)$<br>$screen = (\sigma = 4.822)$	$VR = (W(26) = 0.964, p = 0.48)$<br>$screen = (W(26) = 0.867, p = 0.003)^*$
Number of figure changes 2nd - Scene 7	$VR = (\mu = 5.654)$<br>$screen = (\mu = 7.115)$	$VR = (\sigma = 2.841)$<br>$screen = (\sigma = 3.555)$	$VR = (W(26) = 0.962, p = 0.443)$<br>$screen = (W(26) = 0.936, p = 0.11)$
Number of figure changes 2nd - Scene 8	$VR = (\mu = 5.154)$<br>$screen = (\mu = 4.962)$	$VR = (\sigma = 3.505)$<br>$screen = (\sigma = 2.968)$	$VR = (W(26) = 0.952, p = 0.26)$<br>$screen = (W(26) = 0.949, p = 0.221)$
Number of figure changes 2nd - Scene 9	$VR = (\mu = 2.5)$<br>$screen = (\mu = 3.615)$	$VR = (\sigma = 2.206)$<br>$screen = (\sigma = 2.114)$	$VR = (W(26) = 0.877, p = 0.005)^*$<br>$screen = (W(26) = 0.945, p = 0.175)$
Number of figure changes 2nd - Scene 10	$VR = (\mu = 6.423)$<br>$screen = (\mu = 8.5)$	$VR = (\sigma = 3.26)$<br>$screen = (\sigma = 4.917)$	$VR = (W(26) = 0.908, p = 0.024)^*$<br>$screen = (W(26) = 0.936, p = 0.105)$
Number of figure changes 2nd - Scene 11	$VR = (\mu = 1.538)$<br>$screen = (\mu = 2.615)$	$VR = (\sigma = 1.151)$<br>$screen = (\sigma = 1.982)$	$VR = (W(26) = 0.847, p = 0.001)^*$<br>$screen = (W(26) = 0.901, p = 0.017)^*$
Number of figure changes 2nd - Scene 12	$VR = (\mu = 2.154)$<br>$screen = (\mu = 3.577)$	$VR = (\sigma = 1.994)$<br>$screen = (\sigma = 1.885)$	$VR = (W(26) = 0.878, p = 0.005)^*$<br>$screen = (W(26) = 0.906, p = 0.022)^*$

Table S6.7: Mean, standard deviation (*SD*) and Shapiro-Wilk Test per each scene and impression for the number of figure changes metric for the first (1st), and second (2nd) impression. Asterisks (*) indicate departure from normality ($p < 0.05$).

	Mean	*SD*	Shapiro-Wilk Test
Exciting	$VR = (\mu = 4.346)$<br>$screen = (\mu = 4.926)$	$VR = (\sigma = 1.299)$<br>$screen = (\sigma = 0.979)$	$VR = (W(26) = 0.875, p = 0.005)^{*}$<br>$screen = (W(27) = 0.849, p = 0.001)^{*}$
Being there	$VR = (\mu = 3.731)$<br>$screen = (\mu = 3.556)$	$VR = (\sigma = 1.558)$<br>$screen = (\sigma = 1.449)$	$VR = (W(26) = 0.838, p < 0.001)^{*}$<br>$screen = (W(27) = 0.913, p = 0.027)^{*}$
Present	$VR = (\mu = 3.885)$<br>$screen = (\mu = 3.222)$	$VR = (\sigma = 1.761)$<br>$screen = (\sigma = 1.474)$	$VR = (W(26) = 0.93, p = 0.076)$<br>$screen = (W(27) = 0.92, p = 0.04)^{*}$
Focused	$VR = (\mu = 6.0)$<br>$screen = (\mu = 5.222)$	$VR = (\sigma = 0.734)$<br>$screen = (\sigma = 1.37)$	$VR = (W(26) = 0.821, p < 0.001)^{*}$<br>$screen = (W(27) = 0.839, p < 0.001)^{*}$

Table S6.8: Mean, standard deviation (*SD*) and Shapiro-Wilk Test for the presence questionnaire. Asterisks (*) indicate departure from normality ($p < 0.05$).

S6.4.2 Figure saliency (% time watched)

The figure saliency metric metric results presented in the main document can also be computed per each stimulus' scene. Results are shown in Figures S6.2 to S6.13. Additionally, full statistical tables of the analyses performed in the main document are reported. Results are shown in Tables S6.15 to S6.17.

S6.4.3 Number of figure changes

The number of figure changes metric results presented in the main document can also be computed per each stimulus' scene. Results are shown in Figure S6.14. Additionally, full statistical tables of the analyses performed in the main document are reported. Results are shown in Tables S6.18 to S6.20.

S6.5 Correlation statistical analyses

In this subsection, we present correlation tables that discuss the results outlined in the main document. A comprehensive statistical table showcasing the correlation analysis between the SAT indices and the Greenberg-Strickland questionnaire (subjective measures) alongside global AUC, figure saliency, and the number of figure changes (objective measures) is provided in Table S6.21. Additionally, to reinforce these correlation results, we introduce the pairwise plot depicted in Figure S6.15. Furthermore, Spearman's Rank-Order Correlation coefficient of the Greenberg-Strickland questionnaire, as discussed in the main document, is included in

Index	Test		VR 1st vs. screen 1st	VR 2nd vs. screen 2nd
Number of propositions	Mann-Whitney U	U	288.500	334.500
		p	0.265	0.949
		$p_{adjusted}$	0.688	0.949
Pertinence Index	Mann-Whitney U	U	266.000	285.000
		p	0.130	0.332
		$p_{adjusted}$	0.530	0.637
Salience Index	Mann-Whitney U	U	338.000	310.500
		p	0.817	0.613
		$p_{adjusted}$	0.894	0.697
Cognition Index	Mann-Whitney U	U	312.000	194.500
		p	0.488	0.009*
		$p_{adjusted}$	0.488	0.044*
Affective Index	Mann-Whitney U	U	306.500	192.500
		p	0.428	0.008*
		$p_{adjusted}$	0.688	0.044*
Animation Index	Mann-Whitney U	U	345.000	208.500
		p	0.912	0.017*
		$p_{adjusted}$	0.912	0.056
Person Index	Mann-Whitney U	U	-	302.000
		p	-	0.377
		$p_{adjusted}$	-	0.637
Problem-Solving Index	Mann-Whitney U	U	-	296.500
		p	-	0.328
		$p_{adjusted}$	-	0.637

Table S6.9: Statistical analyses of the social attribution index of the *VR* and *screen* groups across the same impression. We also report the adjusted p-value using the Benjamini-Hochberg method. Asterisks (*) indicate indicate statistically significant differences ($p < 0.05$).

Index	Test		VR 1st vs. VR 2nd	screen 1st vs. screen 2nd
Number of propositions	Wilcoxon Signed-Rank	Z	-1.617	-0.166
		p	0.106	0.869
		$p_{adjusted}$	0.109	0.869
Pertinence Index	Wilcoxon Signed-Rank	Z	-3.915	-4.407
		p	0.000*	0.000*
		$p_{adjusted}$	0.000*	0.000*
Salience Index	Wilcoxon Signed-Rank	Z	-3.284	-3.088
		p	0.001*	0.002*
		$p_{adjusted}$	0.003*	0.003*
Cognition Index	Wilcoxon Signed-Rank	Z	-3.340	-1.435
		p	0.001*	0.151
		$p_{adjusted}$	0.001*	0.177
Affective Index	Wilcoxon Signed-Rank	Z	-3.492	-1.709
		p	0.000*	0.088
		$p_{adjusted}$	0.000*	0.120
Animation Index	Wilcoxon Signed-Rank	Z	-4.185	-3.808
		p	0.000*	0.000*
		$p_{adjusted}$	0.000*	0.000*

Table S6.10: Statistical analyses of the SAT indices of a same group across impressions. We also report the adjusted p-value using the Benjamini-Hochberg method. Asterisks (*) indicate indicate statistically significant differences ($p < 0.05$).

Index	Test		(VR 1st - VR 2nd) vs. (screen 1st - screen 2nd)
Number of propositions	Mann-Whitney U	*U*	279.500
		p	0.284
Pertinence Index	Mann-Whitney U	*U*	303.000
		p	0.522
Salience Index	Mann-Whitney U	*U*	654.000
		p	0.522
Cognition Index	Mann-Whitney U	*U*	232.500
		p	0.054
Affective Index	Mann-Whitney U	*U*	224.000
		p	0.037*
Animation Index	Mann-Whitney U	*U*	314.000
		p	0.660

Table S6.11: Statistical analyses of the social attribution index of the *VR* and *screen* for the interaction time (first impression, second impression) vs. modality (*screen*, *VR*). Asterisks (*) indicate indicate statistically significant differences ($p < 0.05$).

Table S6.22, along with correlations with the SAT indices as shown in Table S6.23. Corresponding pairwise plots supporting these results are presented in Figure S6.16 and Figure S6.17, respectively.

Group 1	Figure 1	Group 2	Figure 2	Test		Evaluative	Activity	Potency
VR	Small Triangle (t)	VR	Big Triangle (T)	Wilcoxon Signed-Rank	Z	-2.617	-0.201	-2.309
					p	0.009*	0.840	0.021*
					$p_{adjusted}$	0.015*	0.864	0.033*
VR	Circle (c)	VR	Big Triangle (T)	Wilcoxon Signed-Rank	Z	-3.877	-4.272	-4.227
					p	0.000*	0.000*	0.000*
					$p_{adjusted}$	0.001*	0.000*	0.000*
VR	Circle (c)	VR	Small Triangle (t)	Wilcoxon Signed-Rank	Z	-3.847	-3.917	-3.762
					p	0.000*	0.000*	0.000*
					$p_{adjusted}$	0.001*	0.000*	0.000*
screen	Small Triangle (t)	screen	Big Triangle (T)	Wilcoxon Signed-Rank	Z	-2.684	-0.687	-2.912
					p	0.007*	0.492	0.000*
					$p_{adjusted}$	0.014*	0.617	0.000*
screen	Circle (c)	screen	Big Triangle (T)	Wilcoxon Signed-Rank	Z	-3.448	-4.233	-4.547
					p	0.001*	0.000*	0.000*
					$p_{adjusted}$	0.002*	0.000*	0.000*
screen	Circle (c)	screen	Small Triangle (t)	Wilcoxon Signed-Rank	Z	-3.019	-4.256	-4.125
					p	0.003*	0.000*	0.000*
					$p_{adjusted}$	0.007*	0.000*	0.000*
VR	Big Triangle (T)	screen	Big Triangle (T)	Mann-Whitney U	U	309.000	337.000	324.500
					p	0.452	0.801	0.634
					$p_{adjusted}$	0.457	0.864	0.824
VR	Small Triangle (t)	screen	Small Triangle (t)	Mann-Whitney U	U	292.500	283.000	333.000
					p	0.296	0.221	0.747
					$p_{adjusted}$	0.386	0.405	0.848
VR	Circle (c)	screen	Circle (c)	Mann-Whitney U	U	308.000	341.000	346.000
					p	0.441	0.857	0.929
					$p_{adjusted}$	0.457	0.864	0.936

Table S6.12: Statistical analyses of the Greenberg-Strickland questionnaire, comparing evaluations of the *VR* and *screen* groups and between themselves. We also report the adjusted p-value using the Benjamini-Hochberg method. Asterisks (*) indicate indicate statistically significant differences ($p < 0.05$).

Interaction	Differences	Test	U	p
two-way	(Big Triangle, evaluative) - (Circle , evaluative)	Mann-Whitney U	305.000	0.412
two-way	(Big Triangle, evaluative) - (Small Triangle, evaluative)	Mann-Whitney U	344.000	0.901
two-way	(Small Triangle, evaluative) - (Circle, evaluative)	Mann-Whitney U	245.000	0.058
two-way	(Big Triangle, activity) - (Circle , activity)	Mann-Whitney U	336.000	0.789
two-way	(Big Triangle, activity) - (Small Triangle, activity)	Mann-Whitney U	311.000	0.475
two-way	(Small Triangle, activity) - (Circle, activity)	Mann-Whitney U	289.500	0.270
two-way	(Big Triangle, potency) - (Circle , potency)	Mann-Whitney U	334.000	0.762
two-way	(Big Triangle, potency) - (Small Triangle, potency)	Mann-Whitney U	349.000	0.971
two-way	(Small Triangle, potency) - (Circle, potency)	Mann-Whitney U	342.000	0.872
three-way	[(Big Triangle, evaluative) - (Circle , evaluative)] - [(Big Triangle, activity) - (Circle, activity)]	Mann-Whitney U	316.500	0.539
three-way	[(Big Triangle, evaluative) - (Circle , evaluative)] - [(Big Triangle, potency) - (Circle, potency)]	Mann-Whitney U	325.000	0.643
three-way	[(Big Triangle, activity) - (Circle , activity)] - [(Big Triangle, potency) - (Circle, potency)]	Mann-Whitney U	320.000	0.580
three-way	[(Big Triangle, evaluative) - (Small Triangle, evaluative)] - [(Big Triangle, activity) - (Small Triangle, activity)]	Mann-Whitney U	320.000	0.581
three-way	[(Big Triangle, evaluative) - (Small Triangle, evaluative)] - [(Big Triangle, potency) - (Small Triangle, potency)]	Mann-Whitney U	333.500	0.755
three-way	[(Big Triangle, activity) - (Small Triangle, activity)] - [(Big Triangle, potency) - (Small Triangle, potency)]	Mann-Whitney U	339.000	0.831
three-way	[(Small Triangle, evaluative) - (Circle , evaluative)] - [(Small Triangle, activity) - (Circle, activity)]	Mann-Whitney U	243.500	0.055
three-way	[(Small Triangle, evaluative) - (Circle , evaluative)] - [(Small Triangle, potency) - (Circle, potency)]	Mann-Whitney U	277.000	0.187
three-way	[(Small Triangle, activity) - (Circle , activity)] - [(Small Triangle, potency) - (Circle, potency)]	Mann-Whitney U	260.500	0.106

Table S6.13: Two-way and three-way interaction for the Greenberg-Strickland questionnaire. Asterisks (*) indicate indicate statistically significant differences ($p < 0.05$).

Group 1	Group 2	Test		Global AUC
VR 1st	screen 1st	Mann-Whitney U	U	234.000
			p	0.057
			$p_{adjusted}$	0.078
VR 2nd	screen 2nd	Mann-Whitney U	U	227.500
			p	0.043*
			$p_{adjusted}$	0.078
VR 1st	VR 2nd	Wilcoxon Signed-Rank	Z	-1.105
			p	0.269
			$p_{adjusted}$	0.269
screen 1st	screen 2nd	Wilcoxon Signed-Rank	Z	-2.085
			p	0.037*
			$p_{adjusted}$	0.078

Table S6.14: Statistical analyses of the global AUC metric. We also report the adjusted p-value using the Benjamini-Hochberg method. Asterisks (*) indicate indicate statistically significant differences ($p < 0.05$).

Group 1	Figure 1	Group 2	Figure 2	Test		Figure saliency 1st	Figure saliency 2nd
VR	Small Triangle (t)	VR	Big Triangle (T)	Wilcoxon Signed-Rank	Z	-4.457	-4.457
					p	0.000*	0.000*
					$p_{adjusted}$	0.000*	0.000*
VR	Circle (c)	VR	Big Triangle (T)	Wilcoxon Signed-Rank	Z	-4.457	-4.457
					p	0.000*	0.000*
					$p_{adjusted}$	0.000*	0.000*
VR	Circle (c)	VR	Small Triangle (t)	Wilcoxon Signed-Rank	Z	-1.714	-0.165
					p	0.086	0.869
					$p_{adjusted}$	0.133	0.869
screen	Small Triangle (t)	screen	Big Triangle (T)	Wilcoxon Signed-Rank	Z	-4.457	-4.457
					p	0.000*	0.000*
					$p_{adjusted}$	0.000*	0.000*
screen	Circle (c)	screen	Big Triangle (T)	Wilcoxon Signed-Rank	Z	-4.457	-4.457
					p	0.000*	0.000*
					$p_{adjusted}$	0.000*	0.000*
screen	Circle (c)	screen	Small Triangle (t)	Wilcoxon Signed-Rank	Z	-1.740	-0.394
					p	0.082	0.694
					$p_{adjusted}$	0.721	0.147
VR	Big Triangle (T)	screen	Big Triangle (T)	Mann-Whitney U	U	318.000	247.00
					p	0.714	0.096
					$p_{adjusted}$	0.714	0.147
VR	Small Triangle (t)	screen	Small Triangle (t)	Mann-Whitney U	U	291.000	267.000
					p	0.390	0.194
					$p_{adjusted}$	0.444	0.253
VR	Circle (c)	screen	Circle (c)	Mann-Whitney U	U	268.000	205.000
					p	0.200	0.015*
					$p_{adjusted}$	0.262	0.028*

Table S6.15: Statistical analyses of the figure saliency metric between the *VR* and *screen* groups and between themselves. We also report the adjusted p-value using the Benjamini-Hochberg method. Asterisks (*) indicate indicate statistically significant differences ($p < 0.05$).

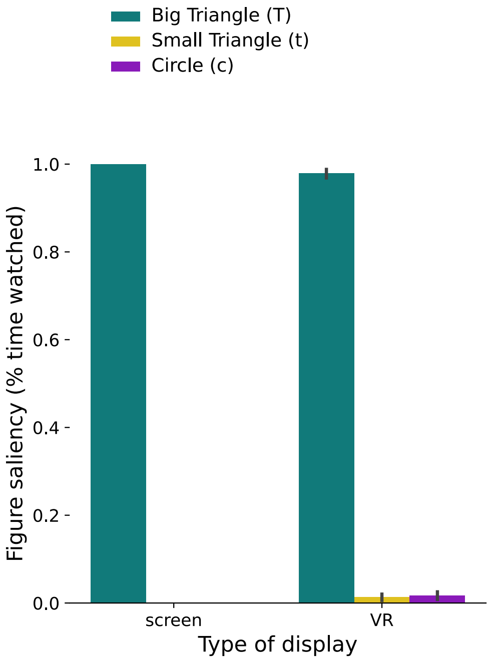

(a) First impression.

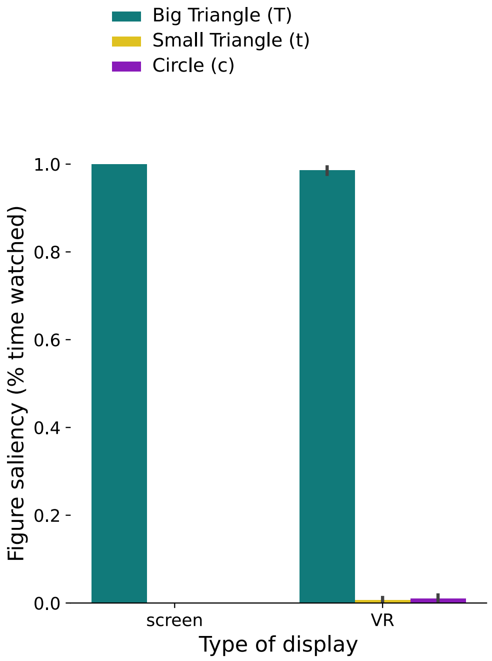

(b) Second impression.

Figure S6.2: Figure saliency metric in scene 1 per each group in the first and second impression, respectively. Scene descriptions are presented in the main paper. Error bars correspond to a 95% confidence interval.

Figure	Test		VR 1st vs. VR 2nd	screen 1st vs. screen 2nd
Big Triangle (T)	Wilcoxon Signed-Rank	Z	-1.841	-1.170
		p	0.066	0.242
Small Triangle (t)	Wilcoxon Signed-Rank	Z	-1.816	-1.762
		p	0.069	0.078
Circle (c)	Wilcoxon Signed-Rank	Z	-0.851	-0.821
		p	0.395	0.412

Table S6.16: Statistical analyses of the figure saliency metric per groups in the same group across impressions. Asterisks (*) indicate statistically significant differences ($p < 0.05$).

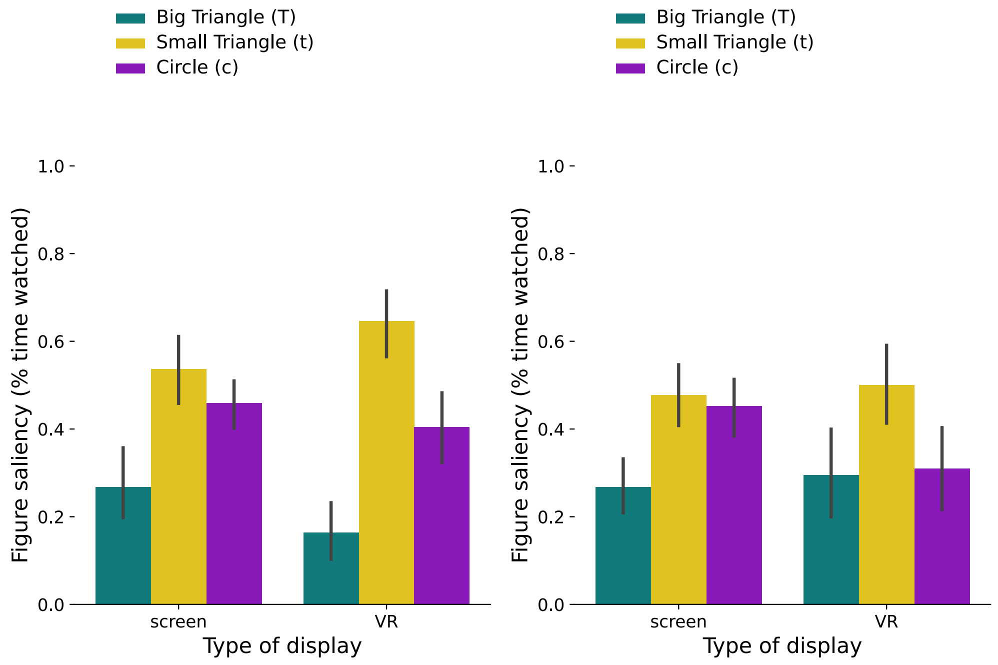

(a) First impression. **(b)** Second impression.

Figure S6.3: Figure saliency metric in scene 2 per each group in the first and second impression, respectively. Scene descriptions are presented in the main paper. Error bars correspond to a 95% confidence interval.

**Interaction**	**Differences**	**Test**	**U**	**p**
two-way	(Big Triangle, first impression) - (Big Triangle, second impression)	Mann-Whitney U	274.000	0.337
two-way	(Small Triangle, first impression) - (Small Triangle, second impression)	Mann-Whitney U	299.000	0.624
two-way	(Circle, first impression) - (Circle, second impression)	Mann-Whitney U	267.000	0.274
three-way	[(Big Triangle, first impression) - (Circle , first impression)] - [(Big Triangle, second impression) - (Circle, second impression)]	Mann-Whitney U	264.000	0.250
three-way	[(Small Triangle, first impression) - (Big Triangle, first impression)] - [(Small Triangle, second impression) - (Big Triangle, second impression)]	Mann-Whitney U	271.000	0.309
three-way	[(Circle, first impression) - (Small Triangle, first impression)] - [(Circle, second impression) - (Small Triangle, second impression)]	Mann-Whitney U	313.000	0.821

Table S6.17: Two-way and three-way interaction for the figure saliency metric. Asterisks (*) indicate statistically significant differences ($p < 0.05$).

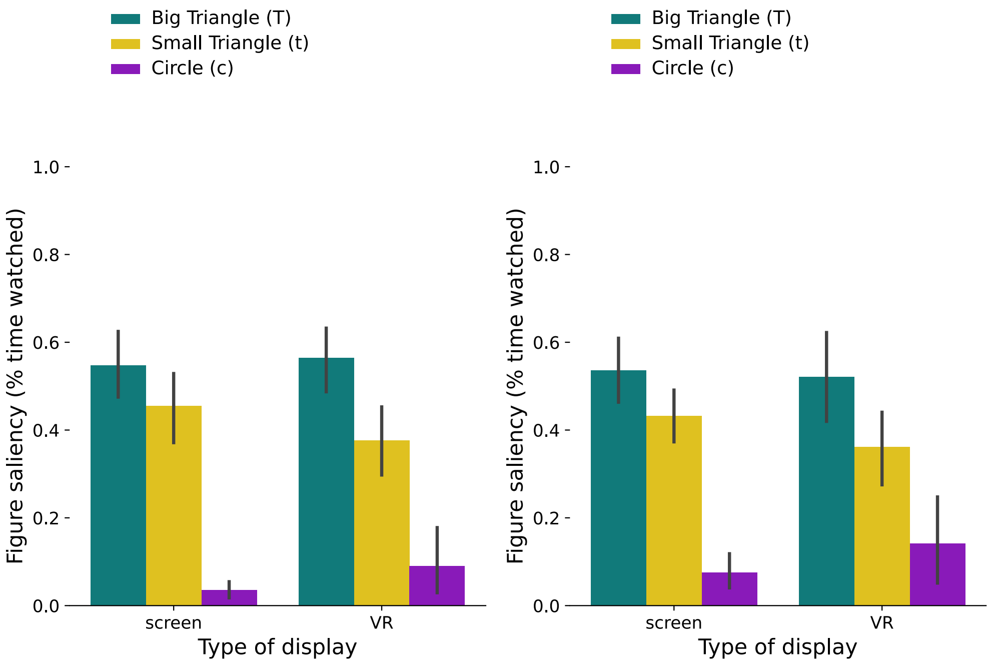

(a) First impression. **(b)** Second impression.

Figure S6.4: Figure saliency metric in scene 3 per each group in the first and second impression, respectively. Scene descriptions are presented in the main paper. Error bars correspond to a 95% confidence interval.

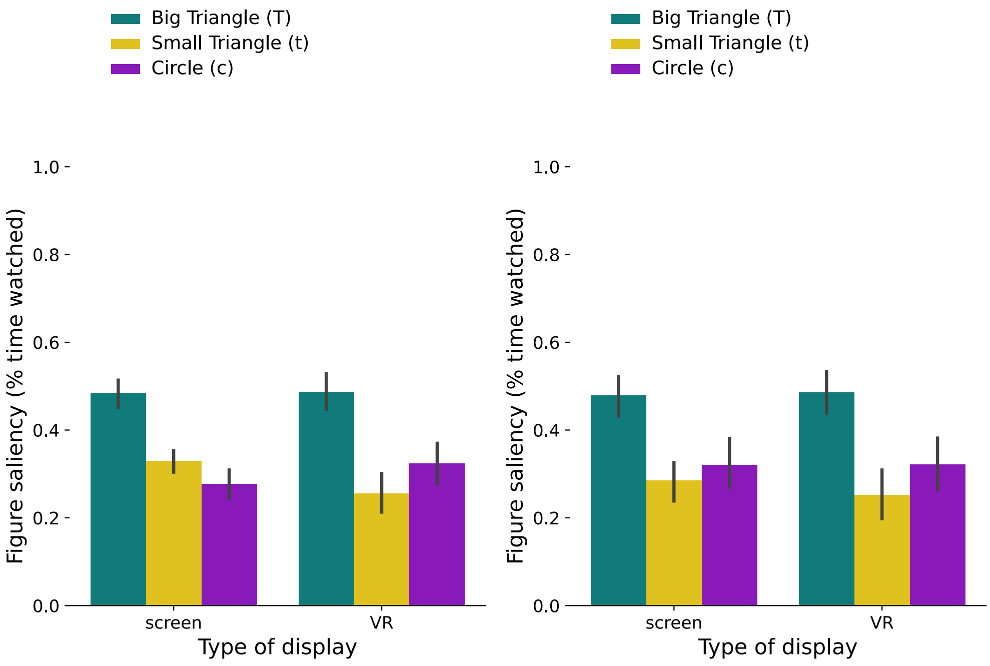

(a) First impression.

(b) Second impression.

Figure S6.5: Figure saliency metric in scene 4 per each group in the first and second impression, respectively. Scene descriptions are presented in the main paper. Error bars correspond to a 95% confidence interval.

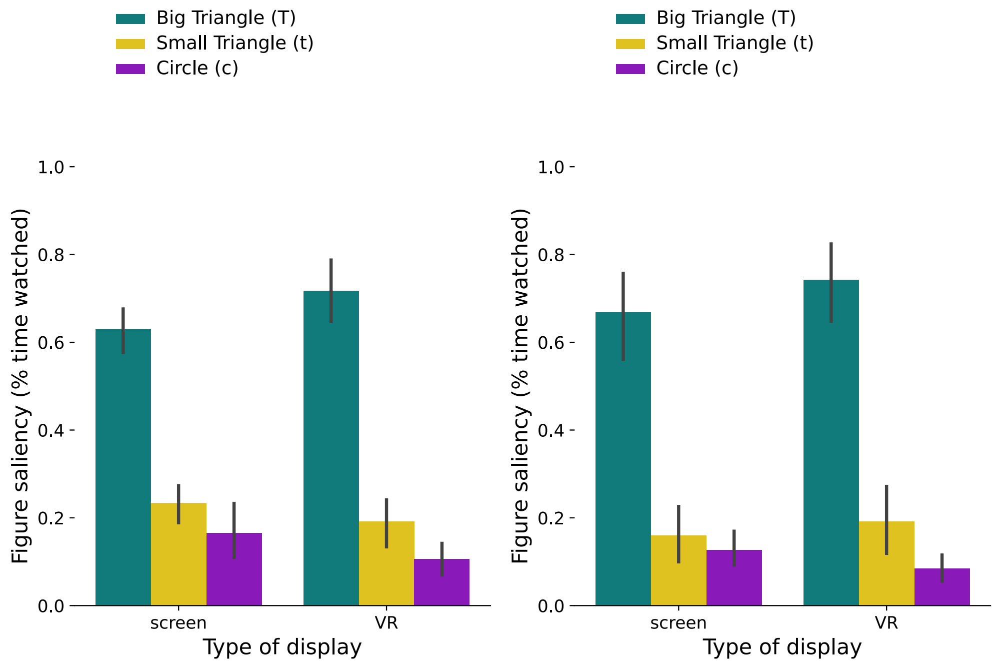

(a) First impression. **(b)** Second impression.

Figure S6.6: Figure saliency metric in scene 5 per each group in the first and second impression, respectively. Scene descriptions are presented in the main paper. Error bars correspond to a 95% confidence interval.

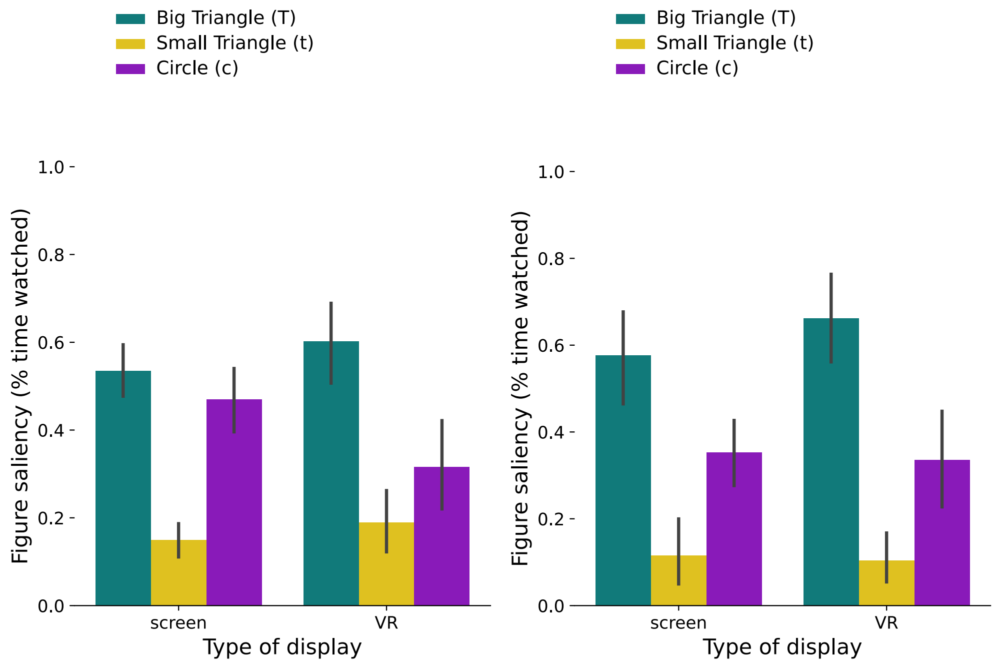

(a) First impression.

(b) Second impression.

Figure S6.7: Figure saliency metric in scene 6 per each group in the first and second impression, respectively. Scene descriptions are presented in the main paper. Error bars correspond to a 95% confidence interval.

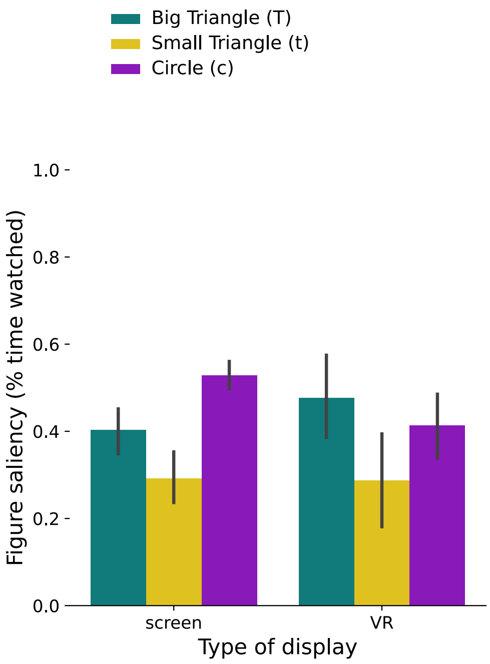

(a) First impression.

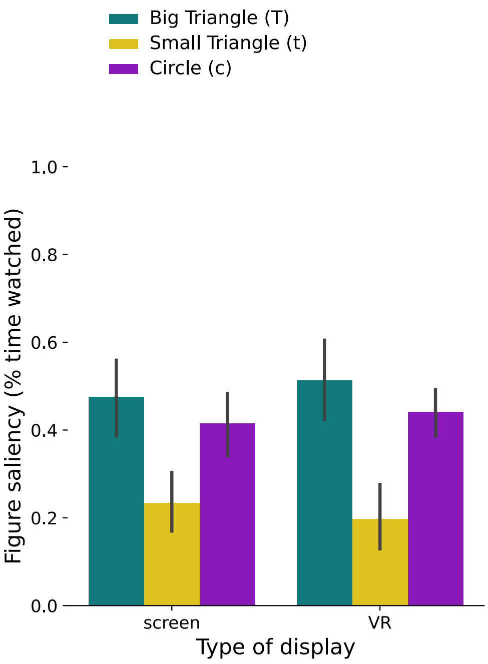

(b) Second impression.

Figure S6.8: Figure saliency metric in scene 7 per each group in the first and second impression, respectively. Scene descriptions are presented in the main paper. Error bars correspond to a 95% confidence interval.

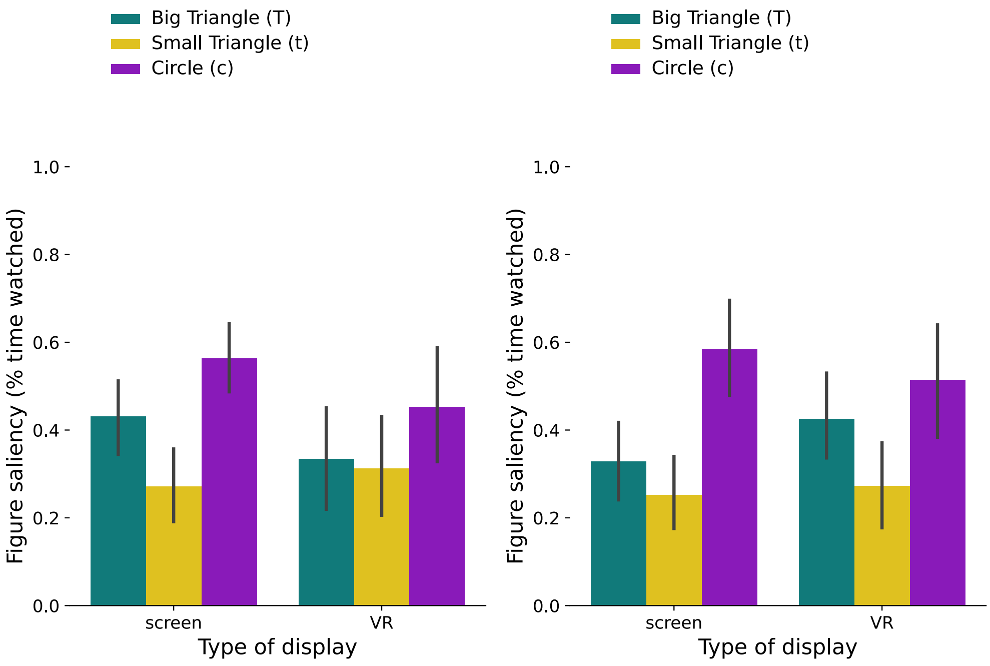

(a) First impression. **(b)** Second impression.

Figure S6.9: Figure saliency metric in scene 8 per each group in the first and second impression, respectively. Scene descriptions are presented in the main paper. Error bars correspond to a 95% confidence interval.

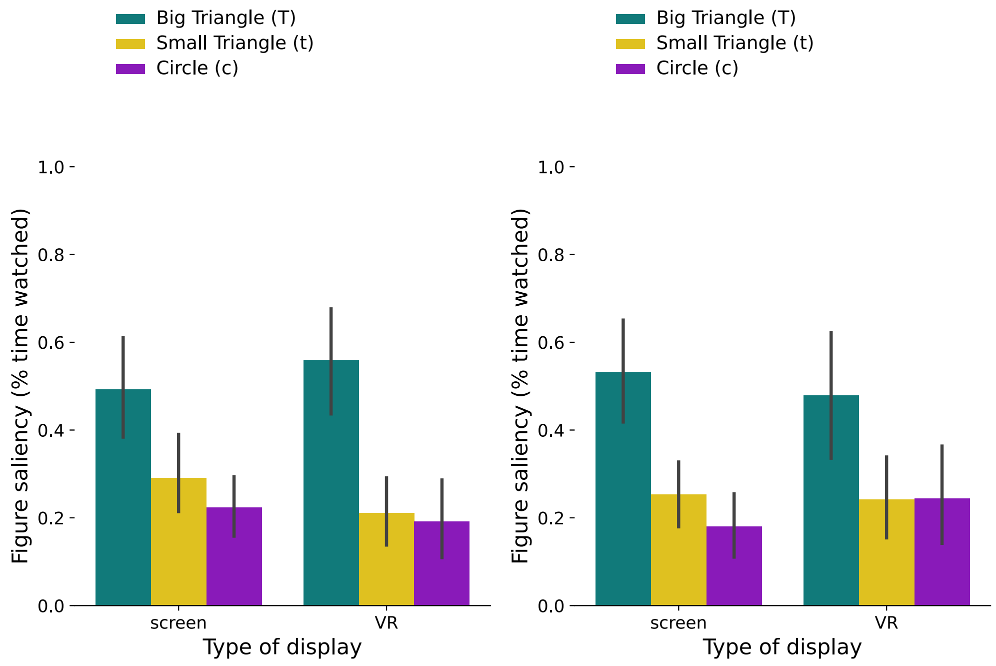

(a) First impression. **(b)** Second impression.

Figure S6.10: Figure saliency metric in scene 9 per each group in the first and second impression, respectively. Scene descriptions are presented in the main paper. Error bars correspond to a 95% confidence interval.

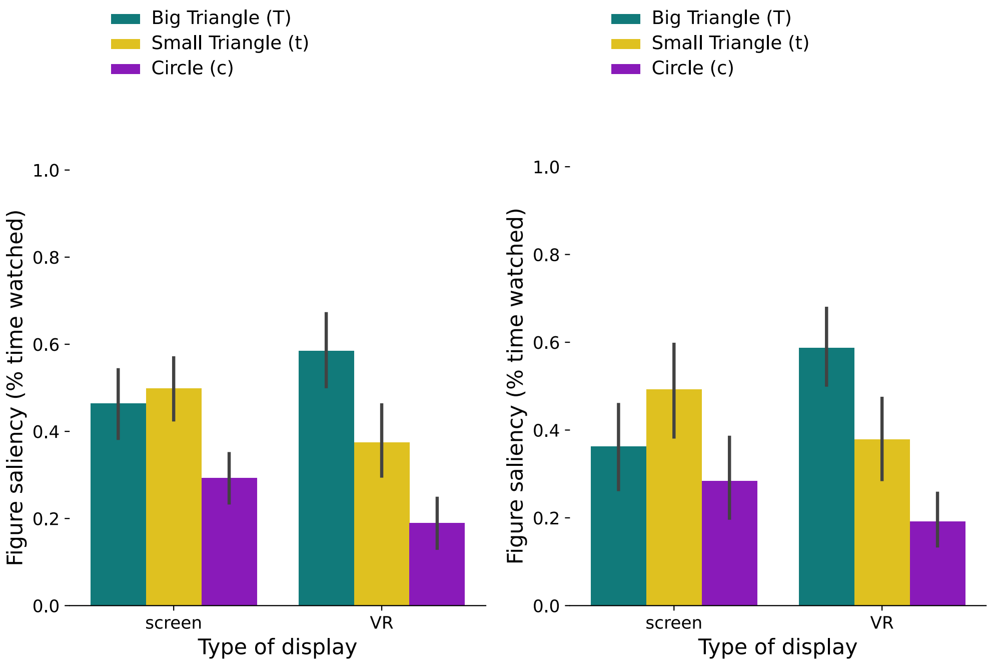

(a) First impression.

(b) Second impression.

Figure S6.11: Figure saliency metric in scene 10 per each group in the first and second impression, respectively. Scene descriptions are presented in the main paper. Error bars correspond to a 95% confidence interval.

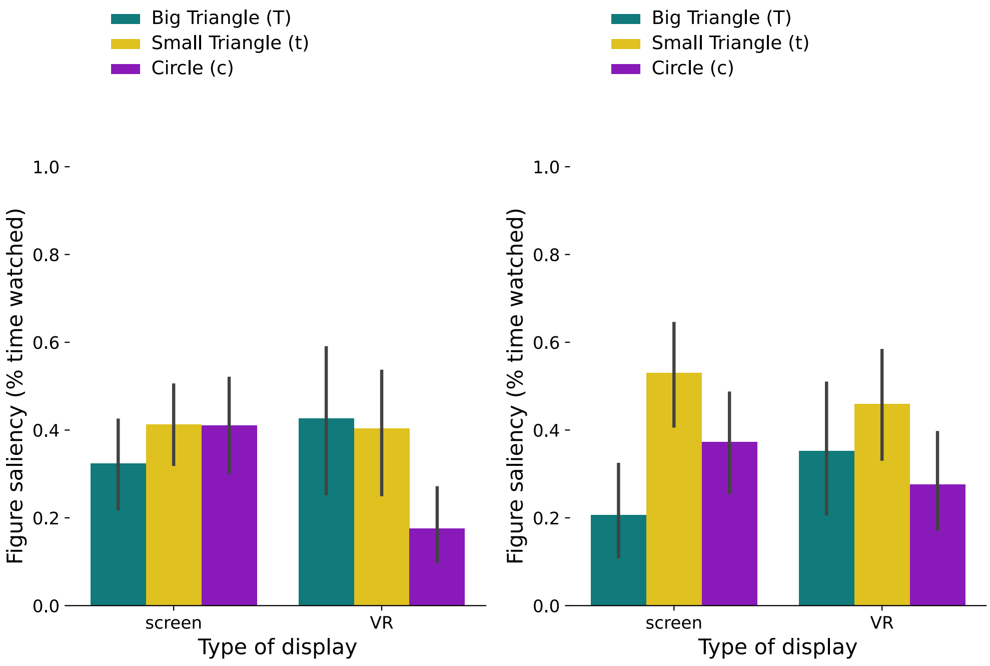

(a) First impression.

(b) Second impression.

Figure S6.12: Figure saliency metric in scene 11 per each group in the first and second impression, respectively. Scene descriptions are presented in the main paper. Error bars correspond to a 95% confidence interval.

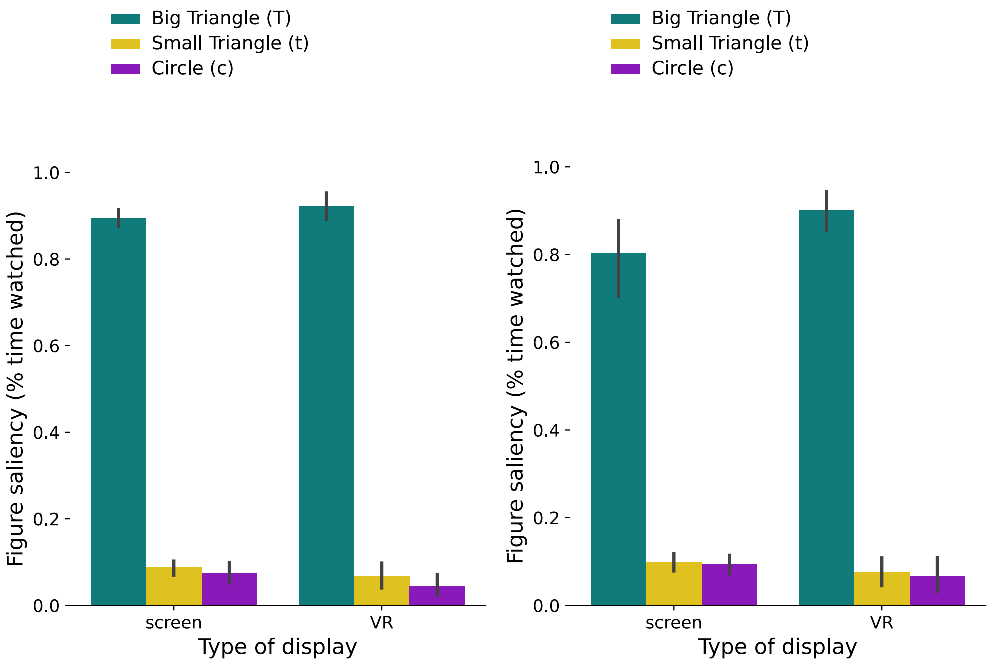

(a) First impression. **(b)** Second impression.

Figure S6.13: Figure saliency metric in scene 12 per each group in the first and second impression, respectively. Scene descriptions are presented in the main paper. Error bars correspond to a 95% confidence interval.

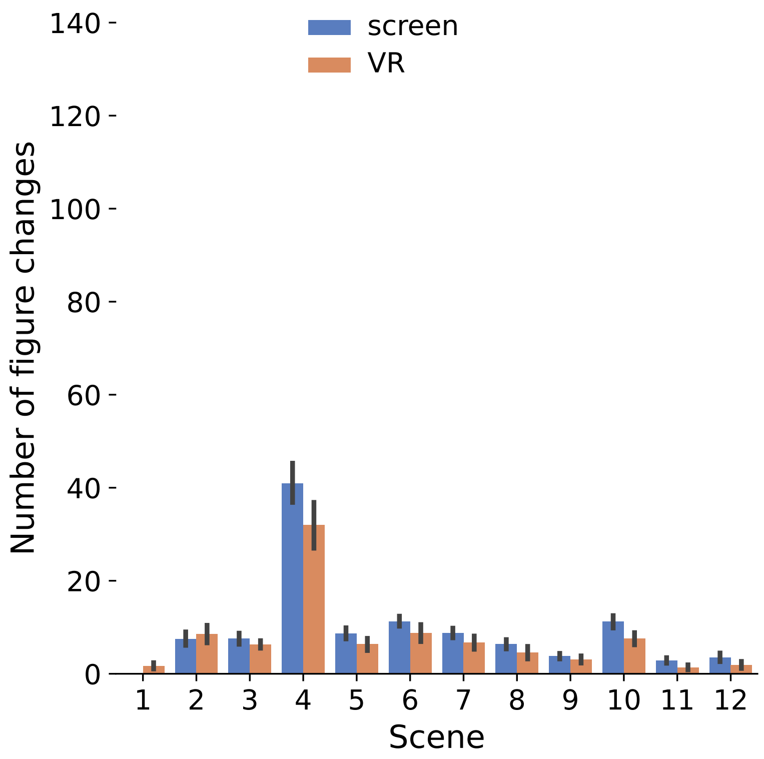

(a) First impression.

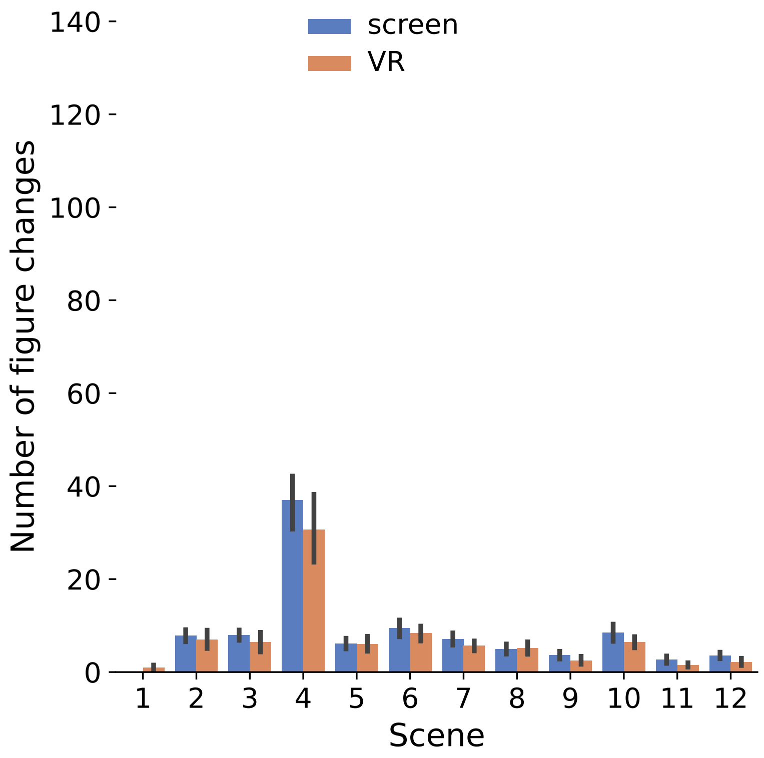

(b) Second impression.

Figure S6.14: Number of figure changes per scene for the first and second impression, respectively. Scene descriptions are presented in the main paper. Error bars correspond to a 95% confidence interval.

Group 1	Group 2	Test		Number of figure changes
VR 1st	screen 1st	Mann-Whitney U	U	164.000
			p	0.001*
			$p_{adjusted}$	0.006*
VR 2nd	screen 2nd	Mann-Whitney U	U	237.500
			p	0.066
			$p_{adjusted}$	0.108
VR 1st	VR 2nd	Wilcoxon Signed-Rank	Z	-1.480
			p	0.139
			$p_{adjusted}$	0.139
screen 1st	screen 2nd	Wilcoxon Signed-Rank	Z	-1.758
			p	0.079
			$p_{adjusted}$	0.108

Table S6.18: Statistical analyses of the number of figure changes metric. We also report the adjusted p-value using the Benjamini-Hochberg method. Asterisks (*) indicate indicate statistically significant differences ($p < 0.05$).

Scene	Group 1	Group 2	Test	U	p	$p_{adjusted}$
1	VR 1st	screen 1st	Mann-Whitney U	156.000	0.000^{*}	0.000^{*}
2	VR 1st	screen 1st	Mann-Whitney U	301.500	0.502	0.502
3	VR 1st	screen 1st	Mann-Whitney U	215.500	0.024^{*}	0.032^{*}
4	VR 1st	screen 1st	Mann-Whitney U	199.500	0.011^{*}	0.020^{*}
5	VR 1st	screen 1st	Mann-Whitney U	225.000	0.038^{*}	0.046^{*}
6	VR 1st	screen 1st	Mann-Whitney U	200.500	0.012^{*}	0.020^{*}
7	VR 1st	screen 1st	Mann-Whitney U	184.000	0.005^{*}	0.014^{*}
8	VR 1st	screen 1st	Mann-Whitney U	209.500	0.018^{*}	0.028^{*}
9	VR 1st	screen 1st	Mann-Whitney U	244.500	0.083	0.092
10	VR 1st	screen 1st	Mann-Whitney U	151.000	0.001^{*}	0.003^{*}
11	VR 1st	screen 1st	Mann-Whitney U	158.500	0.001^{*}	0.003^{*}
12	VR 1st	screen 1st	Mann-Whitney U	201.000	0.011^{*}	0.020^{*}

Table S6.19: Statistical analyses of the number of figure changes per scene in the first impression. We also report the adjusted p-value using the Benjamini-Hochberg method. Asterisks (*) indicate indicate statistically significant differences ($p < 0.05$).

Scene	Group 1	Group 2	Test	*U*	*p*	$p_{adjusted}$
1	VR 2nd	screen 2nd	Mann-Whitney U	195.000	0.000*	0.003*
2	VR 2nd	screen 2nd	Mann-Whitney U	288.500	0.363	0.442
3	VR 2nd	screen 2nd	Mann-Whitney U	213.500	0.022*	0.091
4	VR 2nd	screen 2nd	Mann-Whitney U	250.000	0.107	0.163
5	VR 2nd	screen 2nd	Mann-Whitney U	329.000	0.869	0.955
6	VR 2nd	screen 2nd	Mann-Whitney U	275.000	0.247	0.335
7	VR 2nd	screen 2nd	Mann-Whitney U	231.500	0.050	0.101
8	VR 2nd	screen 2nd	Mann-Whitney U	337.000	0.985	0.993
9	VR 2nd	screen 2nd	Mann-Whitney U	229.000	0.044*	0.101
10	VR 2nd	screen 2nd	Mann-Whitney U	234.500	0.057	0.101
11	VR 2nd	screen 2nd	Mann-Whitney U	225.000	0.034*	0.101
12	VR 2nd	screen 2nd	Mann-Whitney U	207.500	0.015*	0.091

Table S6.20: Statistical analyses of the number of figure changes per scene in the second impression. We also report the adjusted p-value using the Benjamini-Hochberg method. Asterisks (*) indicate indicate statistically significant differences ($p < 0.05$).

	Global AUC 1st	Global AUC 2nd	Figure saliency T 1st	Figure saliency t 1st	Figure saliency c 1st	Figure saliency T 2nd	Figure saliency t 2nd	Figure saliency c 2nd	Number of figure changes 1st	Number of figure changes 2nd
No. of propositions 1st	$VR = (r_s(26) = -0.189, p = 0.356)$<br>$screen = (r_s(26) = 0.148, p = 0.471)$	$VR = (r_s(26) = -0.371, p = 0.062)$<br>$screen = (r_s(26) = 0.165, p = 0.421)$	$VR = (r_s(26) = 0.316, p = 0.116)$<br>$screen = (r_s(26) = 0.344, p = 0.085)$	$VR = (r_s(26) = -0.188, p = 0.358)$<br>$screen = (r_s(26) = -0.092, p = 0.653)$	$VR = (r_s(26) = -0.355, p = 0.075)$<br>$screen = (r_s(26) = -0.243, p = 0.231)$	$VR = (r_s(26) = 0.32, p = 0.111)$<br>$screen = (r_s(26) = 0.003, p = 0.989)$	$VR = (r_s(26) = -0.263, p = 0.194)$<br>$screen = (r_s(26) = -0.29, p = 0.151)$	$VR = (r_s(26) = -0.268, p = 0.186)$<br>$screen = (r_s(26) = -0.025, p = 0.905)$	$VR = (r_s(26) = -0.021, p = 0.92)$<br>$screen = (r_s(26) = 0.065, p = 0.751)$	$VR = (r_s(26) = -0.288, p = 0.154)$<br>$screen = (r_s(26) = -0.245, p = 0.228)$
No. of propositions 2nd	$VR = (r_s(26) = -0.314, p = 0.118)$<br>$screen = (r_s(25) = -0.021, p = 0.921)$	$VR = (r_s(26) = -0.31, p = 0.124)$<br>$screen = (r_s(25) = 0.075, p = 0.723)$	$VR = (r_s(26) = 0.178, p = 0.383)$<br>$screen = (r_s(25) = 0.08, p = 0.703)$	$VR = (r_s(26) = 0.015, p = 0.943)$<br>$screen = (r_s(25) = 0.159, p = 0.449)$	$VR = (r_s(26) = -0.121, p = 0.558)$<br>$screen = (r_s(25) = -0.007, p = 0.975)$	$VR = (r_s(26) = -0.088, p = 0.669)$<br>$screen = (r_s(25) = -0.311, p = 0.131)$	$VR = (r_s(26) = 0.018, p = 0.929)$<br>$screen = (r_s(25) = 0.136, p = 0.516)$	$VR = (r_s(26) = 0.213, p = 0.296)$<br>$screen = (r_s(25) = 0.177, p = 0.396)$	$VR = (r_s(26) = 0.04, p = 0.846)$<br>$screen = (r_s(25) = 0.077, p = 0.715)$	$VR = (r_s(26) = -0.042, p = 0.839)$<br>$screen = (r_s(25) = 0.117, p = 0.578)$
Pertinence Index 1st	$VR = (r_s(26) = 0.244, p = 0.231)$<br>$screen = (r_s(26) = -0.122, p = 0.551)$	$VR = (r_s(26) = 0.079, p = 0.7)$<br>$screen = (r_s(26) = 0.466, p = 0.016)^*$	$VR = (r_s(26) = 0.198, p = 0.333)$<br>$screen = (r_s(26) = 0.028, p = 0.893)$	$VR = (r_s(26) = 0.034, p = 0.871)$<br>$screen = (r_s(26) = 0.122, p = 0.552)$	$VR = (r_s(26) = -0.161, p = 0.433)$<br>$screen = (r_s(26) = -0.246, p = 0.226)$	$VR = (r_s(26) = 0.298, p = 0.139)$<br>$screen = (r_s(26) = 0.18, p = 0.38)$	$VR = (r_s(26) = -0.314, p = 0.118)$<br>$screen = (r_s(26) = -0.075, p = 0.717)$	$VR = (r_s(26) = -0.076, p = 0.713)$<br>$screen = (r_s(26) = -0.156, p = 0.446)$	$VR = (r_s(26) = -0.21, p = 0.302)$<br>$screen = (r_s(26) = 0.147, p = 0.475)$	$VR = (r_s(26) = -0.105, p = 0.611)$<br>$screen = (r_s(26) = 0.194, p = 0.341)$
Pertinence Index 2nd	$VR = (r_s(26) = 0.213, p = 0.295)$<br>$screen = (r_s(25) = -0.137, p = 0.513)$	$VR = (r_s(26) = -0.135, p = 0.509)$<br>$screen = (r_s(25) = 0.363, p = 0.075)$	$VR = (r_s(26) = -0.19, p = 0.353)$<br>$screen = (r_s(25) = 0.041, p = 0.845)$	$VR = (r_s(26) = 0.164, p = 0.423)$<br>$screen = (r_s(25) = 0.046, p = 0.827)$	$VR = (r_s(26) = 0.172, p = 0.401)$<br>$screen = (r_s(25) = 0.082, p = 0.697)$	$VR = (r_s(26) = 0.33, p = 0.1)$<br>$screen = (r_s(25) = -0.17, p = 0.415)$	$VR = (r_s(26) = -0.562, p = 0.003)^*$<br>$screen = (r_s(25) = 0.103, p = 0.625)$	$VR = (r_s(26) = 0.119, p = 0.561)$<br>$screen = (r_s(25) = 0.093, p = 0.658)$	$VR = (r_s(26) = -0.104, p = 0.614)$<br>$screen = (r_s(25) = 0.029, p = 0.89)$	$VR = (r_s(26) = -0.529, p = 0.005)^*$<br>$screen = (r_s(25) = 0.421, p = 0.036)^*$
Salience Index 1st	$VR = (r_s(26) = -0.131, p = 0.524)$<br>$screen = (r_s(26) = 0.269, p = 0.185)$	$VR = (r_s(26) = -0.392, p = 0.047)^*$<br>$screen = (r_s(26) = -0.23, p = 0.259)$	$VR = (r_s(26) = 0.206, p = 0.312)$<br>$screen = (r_s(26) = 0.275, p = 0.174)$	$VR = (r_s(26) = -0.155, p = 0.448)$<br>$screen = (r_s(26) = -0.27, p = 0.182)$	$VR = (r_s(26) = -0.148, p = 0.472)$<br>$screen = (r_s(26) = 0.067, p = 0.746)$	$VR = (r_s(26) = 0.092, p = 0.654)$<br>$screen = (r_s(26) = -0.21, p = 0.303)$	$VR = (r_s(26) = -0.133, p = 0.516)$<br>$screen = (r_s(26) = -0.101, p = 0.624)$	$VR = (r_s(26) = -0.001, p = 0.995)$<br>$screen = (r_s(26) = 0.244, p = 0.23)$	$VR = (r_s(26) = 0.032, p = 0.876)$<br>$screen = (r_s(26) = -0.125, p = 0.542)$	$VR = (r_s(26) = -0.215, p = 0.292)$<br>$screen = (r_s(26) = -0.317, p = 0.115)$
Salience Index 2nd	$VR = (r_s(26) = -0.299, p = 0.139)$<br>$screen = (r_s(25) = 0.052, p = 0.806)$	$VR = (r_s(26) = -0.03, p = 0.886)$<br>$screen = (r_s(25) = 0.089, p = 0.672)$	$VR = (r_s(26) = 0.333, p = 0.097)$<br>$screen = (r_s(25) = 0.104, p = 0.621)$	$VR = (r_s(26) = -0.086, p = 0.678)$<br>$screen = (r_s(25) = 0.053, p = 0.801)$	$VR = (r_s(26) = -0.348, p = 0.081)$<br>$screen = (r_s(25) = -0.16, p = 0.444)$	$VR = (r_s(26) = -0.254, p = 0.211)$<br>$screen = (r_s(25) = -0.008, p = 0.969)$	$VR = (r_s(26) = 0.393, p = 0.047)^*$<br>$screen = (r_s(25) = -0.205, p = 0.325)$	$VR = (r_s(26) = 0.15, p = 0.464)$<br>$screen = (r_s(25) = 0.124, p = 0.556)$	$VR = (r_s(26) = 0.079, p = 0.701)$<br>$screen = (r_s(25) = -0.031, p = 0.884)$	$VR = (r_s(26) = 0.265, p = 0.19)$<br>$screen = (r_s(25) = -0.415, p = 0.039)^*$
Cognition Index 1st	$VR = (r_s(26) = 0.189, p = 0.354)$<br>$screen = (r_s(26) = -0.062, p = 0.765)$	$VR = (r_s(26) = 0.19, p = 0.353)$<br>$screen = (r_s(26) = -0.091, p = 0.658)$	$VR = (r_s(26) = 0.001, p = 0.997)$<br>$screen = (r_s(26) = 0.159, p = 0.439)$	$VR = (r_s(26) = -0.129, p = 0.53)$<br>$screen = (r_s(26) = -0.186, p = 0.363)$	$VR = (r_s(26) = -0.095, p = 0.644)$<br>$screen = (r_s(26) = 0.039, p = 0.85)$	$VR = (r_s(26) = -0.022, p = 0.914)$<br>$screen = (r_s(26) = -0.218, p = 0.284)$	$VR = (r_s(26) = 0.077, p = 0.707)$<br>$screen = (r_s(26) = -0.077, p = 0.71)$	$VR = (r_s(26) = -0.087, p = 0.674)$<br>$screen = (r_s(26) = 0.203, p = 0.319)$	$VR = (r_s(26) = -0.116, p = 0.573)$<br>$screen = (r_s(26) = 0.048, p = 0.816)$	$VR = (r_s(26) = -0.013, p = 0.95)$<br>$screen = (r_s(26) = -0.13, p = 0.528)$
Cognition Index 2nd	$VR = (r_s(26) = -0.301, p = 0.135)$<br>$screen = (r_s(25) = -0.011, p = 0.959)$	$VR = (r_s(26) = -0.028, p = 0.89)$<br>$screen = (r_s(25) = 0.287, p = 0.165)$	$VR = (r_s(26) = -0.027, p = 0.894)$<br>$screen = (r_s(25) = 0.133, p = 0.527)$	$VR = (r_s(26) = -0.035, p = 0.866)$<br>$screen = (r_s(25) = -0.213, p = 0.306)$	$VR = (r_s(26) = 0.03, p = 0.884)$<br>$screen = (r_s(25) = 0.05, p = 0.812)$	$VR = (r_s(26) = 0.001, p = 0.995)$<br>$screen = (r_s(25) = 0.128, p = 0.543)$	$VR = (r_s(26) = -0.09, p = 0.662)$<br>$screen = (r_s(25) = 0.097, p = 0.645)$	$VR = (r_s(26) = 0.254, p = 0.21)$<br>$screen = (r_s(25) = -0.072, p = 0.733)$	$VR = (r_s(26) = -0.08, p = 0.697)$<br>$screen = (r_s(25) = -0.266, p = 0.2)$	$VR = (r_s(26) = -0.225, p = 0.269)$<br>$screen = (r_s(25) = 0.087, p = 0.679)$
Affective Index 1st	$VR = (r_s(26) = -0.211, p = 0.3)$<br>$screen = (r_s(26) = 0.047, p = 0.819)$	$VR = (r_s(26) = -0.208, p = 0.309)$<br>$screen = (r_s(26) = 0.064, p = 0.755)$	$VR = (r_s(26) = -0.026, p = 0.9)$<br>$screen = (r_s(26) = -0.172, p = 0.402)$	$VR = (r_s(26) = 0.106, p = 0.605)$<br>$screen = (r_s(26) = 0.211, p = 0.301)$	$VR = (r_s(26) = 0.109, p = 0.597)$<br>$screen = (r_s(26) = -0.034, p = 0.87)$	$VR = (r_s(26) = -0.004, p = 0.985)$<br>$screen = (r_s(26) = 0.233, p = 0.252)$	$VR = (r_s(26) = -0.088, p = 0.671)$<br>$screen = (r_s(26) = 0.087, p = 0.674)$	$VR = (r_s(26) = 0.121, p = 0.557)$<br>$screen = (r_s(26) = -0.224, p = 0.272)$	$VR = (r_s(26) = 0.09, p = 0.661)$<br>$screen = (r_s(26) = -0.078, p = 0.705)$	$VR = (r_s(26) = 0.013, p = 0.948)$<br>$screen = (r_s(26) = 0.103, p = 0.617)$
Affective Index 2nd	$VR = (r_s(26) = 0.306, p = 0.129)$<br>$screen = (r_s(25) = 0.002, p = 0.991)$	$VR = (r_s(26) = 0.111, p = 0.59)$<br>$screen = (r_s(25) = -0.361, p = 0.076)$	$VR = (r_s(26) = 0.043, p = 0.833)$<br>$screen = (r_s(25) = -0.073, p = 0.728)$	$VR = (r_s(26) = 0.028, p = 0.89)$<br>$screen = (r_s(25) = 0.199, p = 0.34)$	$VR = (r_s(26) = -0.065, p = 0.754)$<br>$screen = (r_s(25) = -0.112, p = 0.596)$	$VR = (r_s(26) = 0.031, p = 0.88)$<br>$screen = (r_s(25) = -0.079, p = 0.707)$	$VR = (r_s(26) = 0.066, p = 0.749)$<br>$screen = (r_s(25) = -0.059, p = 0.779)$	$VR = (r_s(26) = -0.249, p = 0.219)$<br>$screen = (r_s(25) = 0.013, p = 0.951)$	$VR = (r_s(26) = 0.062, p = 0.765)$<br>$screen = (r_s(25) = 0.243, p = 0.242)$	$VR = (r_s(26) = 0.275, p = 0.174)$<br>$screen = (r_s(25) = -0.128, p = 0.543)$
Animation Index 1st	$VR = (r_s(26) = -0.237, p = 0.243)$<br>$screen = (r_s(26) = 0.087, p = 0.673)$	$VR = (r_s(26) = -0.234, p = 0.249)$<br>$screen = (r_s(26) = -0.299, p = 0.137)$	$VR = (r_s(26) = -0.104, p = 0.614)$<br>$screen = (r_s(26) = 0.133, p = 0.517)$	$VR = (r_s(26) = -0.071, p = 0.731)$<br>$screen = (r_s(26) = 0.127, p = 0.537)$	$VR = (r_s(26) = 0.111, p = 0.589)$<br>$screen = (r_s(26) = -0.085, p = 0.68)$	$VR = (r_s(26) = -0.074, p = 0.721)$<br>$screen = (r_s(26) = 0.049, p = 0.812)$	$VR = (r_s(26) = 0.025, p = 0.903)$<br>$screen = (r_s(26) = -0.024, p = 0.908)$	$VR = (r_s(26) = -0.097, p = 0.639)$<br>$screen = (r_s(26) = -0.112, p = 0.587)$	$VR = (r_s(26) = 0.029, p = 0.888)$<br>$screen = (r_s(26) = 0.004, p = 0.984)$	$VR = (r_s(26) = 0.079, p = 0.701)$<br>$screen = (r_s(26) = -0.111, p = 0.59)$
Animation Index 2nd	$VR = (r_s(26) = -0.151, p = 0.461)$<br>$screen = (r_s(25) = 0.176, p = 0.401)$	$VR = (r_s(26) = 0.089, p = 0.665)$<br>$screen = (r_s(25) = -0.174, p = 0.405)$	$VR = (r_s(26) = 0.17, p = 0.405)$<br>$screen = (r_s(25) = 0.124, p = 0.555)$	$VR = (r_s(26) = 0.014, p = 0.947)$<br>$screen = (r_s(25) = 0.004, p = 0.987)$	$VR = (r_s(26) = -0.353, p = 0.077)$<br>$screen = (r_s(25) = -0.23, p = 0.269)$	$VR = (r_s(26) = -0.107, p = 0.604)$<br>$screen = (r_s(25) = -0.001, p = 0.997)$	$VR = (r_s(26) = 0.177, p = 0.388)$<br>$screen = (r_s(25) = -0.181, p = 0.387)$	$VR = (r_s(26) = -0.159, p = 0.439)$<br>$screen = (r_s(25) = 0.147, p = 0.484)$	$VR = (r_s(26) = 0.147, p = 0.473)$<br>$screen = (r_s(25) = 0.071, p = 0.738)$	$VR = (r_s(26) = 0.065, p = 0.751)$<br>$screen = (r_s(25) = -0.393, p = 0.052)$
Person Index	$VR = (r_s(26) = 0.025, p = 0.905)$<br>$screen = (r_s(26) = -0.092, p = 0.656)$	$VR = (r_s(26) = 0.26, p = 0.199)$<br>$screen = (r_s(26) = -0.086, p = 0.675)$	$VR = (r_s(26) = -0.357, p = 0.073)$<br>$screen = (r_s(26) = -0.078, p = 0.703)$	$VR = (r_s(26) = 0.246, p = 0.226)$<br>$screen = (r_s(26) = -0.011, p = 0.956)$	$VR = (r_s(26) = 0.262, p = 0.196)$<br>$screen = (r_s(26) = 0.351, p = 0.079)$	$VR = (r_s(26) = -0.124, p = 0.548)$<br>$screen = (r_s(26) = -0.093, p = 0.651)$	$VR = (r_s(26) = 0.104, p = 0.612)$<br>$screen = (r_s(26) = 0.271, p = 0.18)$	$VR = (r_s(26) = 0.057, p = 0.782)$<br>$screen = (r_s(26) = 0.03, p = 0.883)$	$VR = (r_s(26) = 0.197, p = 0.334)$<br>$screen = (r_s(26) = 0.17, p = 0.405)$	$VR = (r_s(26) = 0.061, p = 0.767)$<br>$screen = (r_s(26) = 0.023, p = 0.912)$
Problem-Solving Index	$VR = (r_s(26) = -0.476, p = 0.014)^*$<br>$screen = (r_s(26) = -0.263, p = 0.195)$	$VR = (r_s(26) = -0.178, p = 0.383)$<br>$screen = (r_s(26) = -0.188, p = 0.357)$	$VR = (r_s(26) = 0.266, p = 0.189)$<br>$screen = (r_s(26) = -0.001, p = 0.997)$	$VR = (r_s(26) = -0.105, p = 0.608)$<br>$screen = (r_s(26) = 0.092, p = 0.654)$	$VR = (r_s(26) = -0.235, p = 0.248)$<br>$screen = (r_s(26) = 0.087, p = 0.674)$	$VR = (r_s(26) = -0.359, p = 0.072)$<br>$screen = (r_s(26) = 0.011, p = 0.957)$	$VR = (r_s(26) = 0.458, p = 0.019)^*$<br>$screen = (r_s(26) = 0.296, p = 0.142)$	$VR = (r_s(26) = 0.03, p = 0.885)$<br>$screen = (r_s(26) = -0.069, p = 0.737)$	$VR = (r_s(26) = 0.17, p = 0.407)$<br>$screen = (r_s(26) = 0.044, p = 0.831)$	$VR = (r_s(26) = 0.524, p = 0.006)^*$<br>$screen = (r_s(26) = 0.082, p = 0.689)$
Evaluative T	$VR = (r_s(26) = -0.144, p = 0.482)$<br>$screen = (r_s(26) = 0.278, p = 0.168)$	$VR = (r_s(26) = -0.069, p = 0.739)$<br>$screen = (r_s(26) = -0.017, p = 0.935)$	$VR = (r_s(26) = 0.233, p = 0.253)$<br>$screen = (r_s(26) = 0.291, p = 0.149)$	$VR = (r_s(26) = -0.301, p = 0.135)$<br>$screen = (r_s(26) = -0.16, p = 0.434)$	$VR = (r_s(26) = -0.056, p = 0.787)$<br>$screen = (r_s(26) = -0.275, p = 0.175)$	$VR = (r_s(26) = 0.364, p = 0.068)$<br>$screen = (r_s(26) = 0.165, p = 0.421)$	$VR = (r_s(26) = -0.294, p = 0.145)$<br>$screen = (r_s(26) = -0.231, p = 0.256)$	$VR = (r_s(26) = -0.345, p = 0.085)$<br>$screen = (r_s(26) = -0.042, p = 0.839)$	$VR = (r_s(26) = -0.216, p = 0.289)$<br>$screen = (r_s(26) = 0.014, p = 0.947)$	$VR = (r_s(26) = -0.156, p = 0.445)$<br>$screen = (r_s(26) = -0.175, p = 0.394)$
Evaluative t	$VR = (r_s(26) = 0.118, p = 0.566)$<br>$screen = (r_s(26) = -0.374, p = 0.06)$	$VR = (r_s(26) = 0.053, p = 0.799)$<br>$screen = (r_s(26) = -0.003, p = 0.989)$	$VR = (r_s(26) = 0.023, p = 0.913)$<br>$screen = (r_s(26) = -0.278, p = 0.169)$	$VR = (r_s(26) = 0.127, p = 0.538)$<br>$screen = (r_s(26) = 0.422, p = 0.032)^*$	$VR = (r_s(26) = -0.193, p = 0.344)$<br>$screen = (r_s(26) = -0.082, p = 0.69)$	$VR = (r_s(26) = -0.154, p = 0.452)$<br>$screen = (r_s(26) = -0.149, p = 0.469)$	$VR = (r_s(26) = 0.155, p = 0.448)$<br>$screen = (r_s(26) = -0.065, p = 0.751)$	$VR = (r_s(26) = 0.101, p = 0.625)$<br>$screen = (r_s(26) = 0.362, p = 0.069)$	$VR = (r_s(26) = 0.156, p = 0.446)$<br>$screen = (r_s(26) = 0.063, p = 0.761)$	$VR = (r_s(26) = 0.209, p = 0.305)$<br>$screen = (r_s(26) = 0.094, p = 0.649)$
Evaluative c	$VR = (r_s(26) = 0.034, p = 0.869)$<br>$screen = (r_s(26) = -0.355, p = 0.075)$	$VR = (r_s(26) = 0.16, p = 0.434)$<br>$screen = (r_s(26) = -0.418, p = 0.033)^*$	$VR = (r_s(26) = -0.048, p = 0.818)$<br>$screen = (r_s(26) = -0.18, p = 0.38)$	$VR = (r_s(26) = 0.292, p = 0.148)$<br>$screen = (r_s(26) = 0.194, p = 0.342)$	$VR = (r_s(26) = -0.238, p = 0.241)$<br>$screen = (r_s(26) = 0.054, p = 0.792)$	$VR = (r_s(26) = -0.222, p = 0.275)$<br>$screen = (r_s(26) = -0.108, p = 0.599)$	$VR = (r_s(26) = 0.191, p = 0.349)$<br>$screen = (r_s(26) = 0.216, p = 0.289)$	$VR = (r_s(26) = 0.189, p = 0.355)$<br>$screen = (r_s(26) = 0.04, p = 0.845)$	$VR = (r_s(26) = 0.138, p = 0.501)$<br>$screen = (r_s(26) = 0.115, p = 0.575)$	$VR = (r_s(26) = 0.066, p = 0.748)$<br>$screen = (r_s(26) = 0.273, p = 0.177)$
Activity T	$VR = (r_s(26) = 0.139, p = 0.498)$<br>$screen = (r_s(26) = 0.009, p = 0.964)$	$VR = (r_s(26) = 0.179, p = 0.383)$<br>$screen = (r_s(26) = 0.076, p = 0.713)$	$VR = (r_s(26) = -0.247, p = 0.225)$<br>$screen = (r_s(26) = -0.01, p = 0.963)$	$VR = (r_s(26) = 0.294, p = 0.146)$<br>$screen = (r_s(26) = 0.284, p = 0.16)$	$VR = (r_s(26) = -0.008, p = 0.968)$<br>$screen = (r_s(26) = -0.156, p = 0.447)$	$VR = (r_s(26) = -0.245, p = 0.227)$<br>$screen = (r_s(26) = -0.193, p = 0.344)$	$VR = (r_s(26) = 0.095, p = 0.645)$<br>$screen = (r_s(26) = 0.162, p = 0.429)$	$VR = (r_s(26) = 0.257, p = 0.205)$<br>$screen = (r_s(26) = -0.013, p = 0.948)$	$VR = (r_s(26) = 0.267, p = 0.186)$<br>$screen = (r_s(26) = 0.099, p = 0.632)$	$VR = (r_s(26) = 0.038, p = 0.855)$<br>$screen = (r_s(26) = 0.169, p = 0.409)$
Activity t	$VR = (r_s(26) = 0.031, p = 0.88)$<br>$screen = (r_s(26) = 0.47, p = 0.015)^*$	$VR = (r_s(26) = 0.14, p = 0.496)$<br>$screen = (r_s(26) = 0.06, p = 0.773)$	$VR = (r_s(26) = 0.023, p = 0.912)$<br>$screen = (r_s(26) = 0.145, p = 0.48)$	$VR = (r_s(26) = -0.03, p = 0.884)$<br>$screen = (r_s(26) = -0.141, p = 0.491)$	$VR = (r_s(26) = -0.083, p = 0.686)$<br>$screen = (r_s(26) = 0.27, p = 0.182)$	$VR = (r_s(26) = 0.169, p = 0.408)$<br>$screen = (r_s(26) = -0.155, p = 0.449)$	$VR = (r_s(26) = -0.247, p = 0.224)$<br>$screen = (r_s(26) = 0.14, p = 0.495)$	$VR = (r_s(26) = -0.061, p = 0.768)$<br>$screen = (r_s(26) = -0.039, p = 0.851)$	$VR = (r_s(26) = 0.001, p = 0.996)$<br>$screen = (r_s(26) = 0.194, p = 0.341)$	$VR = (r_s(26) = -0.086, p = 0.676)$<br>$screen = (r_s(26) = 0.07, p = 0.734)$
Activity c	$VR = (r_s(26) = -0.024, p = 0.906)$<br>$screen = (r_s(26) = 0.03, p = 0.886)$	$VR = (r_s(26) = -0.23, p = 0.258)$<br>$screen = (r_s(26) = 0.093, p = 0.652)$	$VR = (r_s(26) = 0.155, p = 0.448)$<br>$screen = (r_s(26) = 0.234, p = 0.249)$	$VR = (r_s(26) = -0.265, p = 0.191)$<br>$screen = (r_s(26) = -0.219, p = 0.282)$	$VR = (r_s(26) = 0.033, p = 0.871)$<br>$screen = (r_s(26) = -0.256, p = 0.207)$	$VR = (r_s(26) = 0.015, p = 0.944)$<br>$screen = (r_s(26) = 0.445, p = 0.023)^*$	$VR = (r_s(26) = 0.024, p = 0.907)$<br>$screen = (r_s(26) = -0.434, p = 0.027)^*$	$VR = (r_s(26) = -0.034, p = 0.869)$<br>$screen = (r_s(26) = -0.258, p = 0.203)$	$VR = (r_s(26) = -0.438, p = 0.025)^*$<br>$screen = (r_s(26) = 0.005, p = 0.982)$	$VR = (r_s(26) = -0.204, p = 0.318)$<br>$screen = (r_s(26) = -0.252, p = 0.215)$
Potency T	$VR = (r_s(26) = -0.067, p = 0.744)$<br>$screen = (r_s(26) = 0.185, p = 0.367)$	$VR = (r_s(26) = 0.303, p = 0.132)$<br>$screen = (r_s(26) = -0.073, p = 0.722)$	$VR = (r_s(26) = -0.064, p = 0.758)$<br>$screen = (r_s(26) = -0.083, p = 0.686)$	$VR = (r_s(26) = -0.208, p = 0.307)$<br>$screen = (r_s(26) = 0.229, p = 0.261)$	$VR = (r_s(26) = 0.212, p = 0.298)$<br>$screen = (r_s(26) = 0.002, p = 0.993)$	$VR = (r_s(26) = -0.083, p = 0.686)$<br>$screen = (r_s(26) = -0.065, p = 0.752)$	$VR = (r_s(26) = 0.232, p = 0.254)$<br>$screen = (r_s(26) = 0.288, p = 0.153)$	$VR = (r_s(26) = -0.183, p = 0.371)$<br>$screen = (r_s(26) = -0.227, p = 0.266)$	$VR = (r_s(26) = -0.201, p = 0.324)$<br>$screen = (r_s(26) = -0.095, p = 0.645)$	$VR = (r_s(26) = 0.103, p = 0.615)$<br>$screen = (r_s(26) = 0.088, p = 0.668)$
Potency t	$VR = (r_s(26) = -0.157, p = 0.445)$<br>$screen = (r_s(26) = 0.354, p = 0.076)$	$VR = (r_s(26) = -0.186, p = 0.364)$<br>$screen = (r_s(26) = 0.246, p = 0.226)$	$VR = (r_s(26) = 0.113, p = 0.583)$<br>$screen = (r_s(26) = -0.074, p = 0.719)$	$VR = (r_s(26) = -0.186, p = 0.362)$<br>$screen = (r_s(26) = 0.018, p = 0.93)$	$VR = (r_s(26) = -0.132, p = 0.52)$<br>$screen = (r_s(26) = 0.343, p = 0.087)$	$VR = (r_s(26) = 0.141, p = 0.492)$<br>$screen = (r_s(26) = -0.108, p = 0.6)$	$VR = (r_s(26) = -0.276, p = 0.173)$<br>$screen = (r_s(26) = -0.19, p = 0.352)$	$VR = (r_s(26) = 0.063, p = 0.762)$<br>$screen = (r_s(26) = 0.171, p = 0.405)$	$VR = (r_s(26) = 0.128, p = 0.532)$<br>$screen = (r_s(26) = 0.391, p = 0.048)^*$	$VR = (r_s(26) = 0.086, p = 0.678)$<br>$screen = (r_s(26) = 0.186, p = 0.363)$
Potency c	$VR = (r_s(26) = -0.085, p = 0.681)$<br>$screen = (r_s(26) = -0.145, p = 0.481)$	$VR = (r_s(26) = -0.082, p = 0.691)$<br>$screen = (r_s(26) = -0.201, p = 0.325)$	$VR = (r_s(26) = 0.029, p = 0.887)$<br>$screen = (r_s(26) = 0.017, p = 0.933)$	$VR = (r_s(26) = -0.141, p = 0.492)$<br>$screen = (r_s(26) = -0.105, p = 0.609)$	$VR = (r_s(26) = 0.073, p = 0.722)$<br>$screen = (r_s(26) = -0.081, p = 0.694)$	$VR = (r_s(26) = 0.144, p = 0.482)$<br>$screen = (r_s(26) = 0.321, p = 0.11)$	$VR = (r_s(26) = -0.267, p = 0.188)$<br>$screen = (r_s(26) = -0.13, p = 0.526)$	$VR = (r_s(26) = 0.153, p = 0.457)$<br>$screen = (r_s(26) = -0.461, p = 0.018)^*$	$VR = (r_s(26) = -0.265, p = 0.191)$<br>$screen = (r_s(26) = 0.201, p = 0.324)$	$VR = (r_s(26) = -0.105, p = 0.61)$<br>$screen = (r_s(26) = 0.265, p = 0.19)$

Table S6.21: Spearman correlations between objective measures (global AUC, figure saliency, number of figure changes) and subjective measures (SAT Indices, Greenberg-Strickland). Asterisks (*) indicate statistically significant differences ($p < 0.05$). Please see the main text for a discussion of the observed effects.

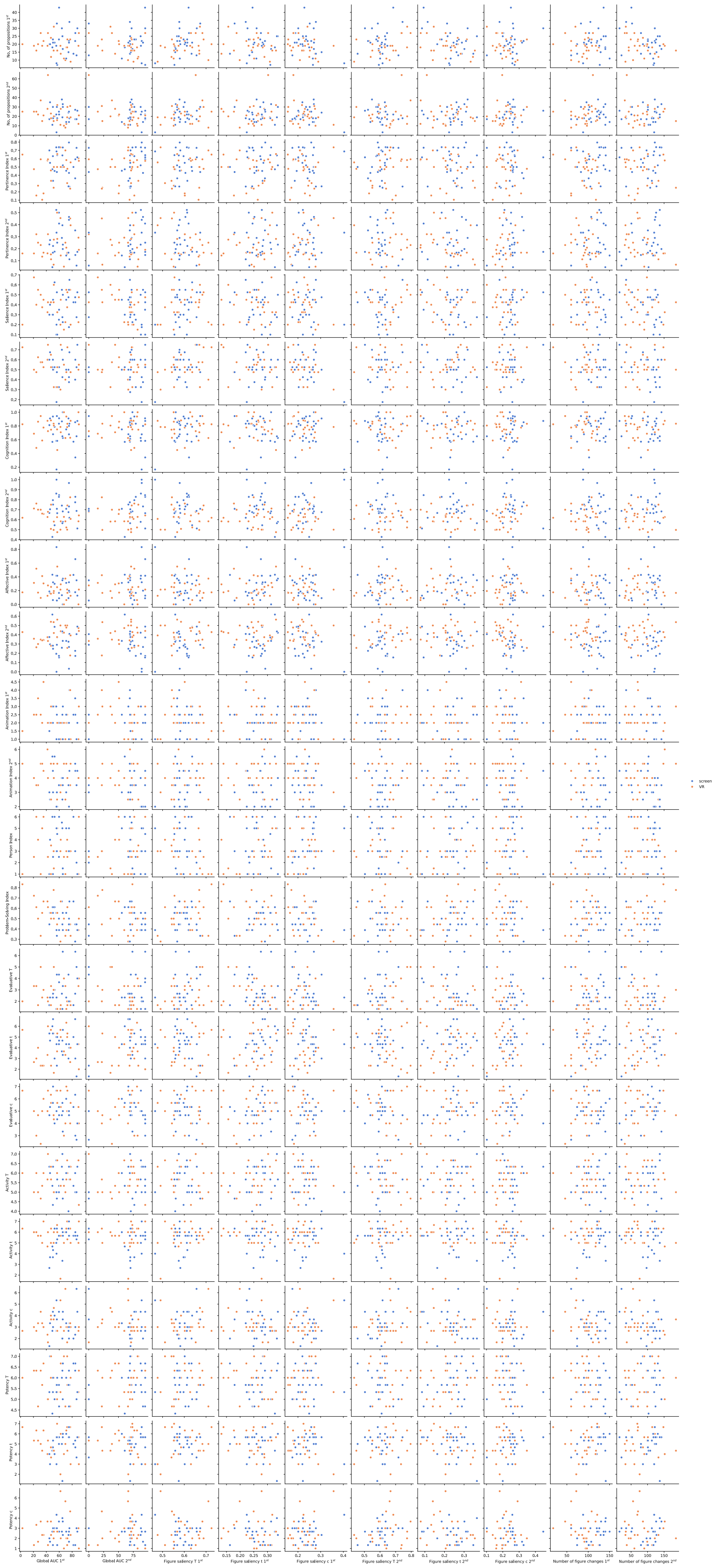

Figure S6.15: Pairwise relationships between objective measures (global AUC, figure saliency, number of figure changes) and subjective measures (SAT Indices, Greenberg-Strickland).

	Evaluative T	Evaluative t	Evaluative c	Activity T	Activity t	Activity c	Potency T	Potency t	Potency c
Evaluative T	-	-	-	-	-	-	-	-	-
Evaluative t	$VR = (r_s(26) = -0.747, p < 0.001)^*$ $screen = (r_s(27) = -0.480, p = 0.011)^*$	-	-	-	-	-	-	-	-
Evaluative c	$VR = (r_s(26) = -0.671, p < 0.001)^*$ $screen = (r_s(27) = -0.414, p = 0.032)^*$	$VR = (r_s(26) = -0.634, p < 0.001)^*$ $screen = (r_s(27) = -0.618, p = 0.001)^*$	-	-	-	-	-	-	-
Activity T	$VR = (r_s(26) = -0.452, p = 0.020)^*$ $screen = (r_s(27) = -0.602, p = 0.001)^*$	$VR = (r_s(26) = 0.502, p = 0.009)^*$ $screen = (r_s(27) = 0.405, p = 0.036)^*$	$VR = (r_s(26) = 0.570, p = 0.002)^*$ $screen = (r_s(27) = 0.288, p = 0.145)$	-	-	-	-	-	-
Activity t	$VR = (r_s(26) = 0.152, p = 0.460)$ $screen = (r_s(27) = 0.381, p = 0.050)^*$	$VR = (r_s(26) = -0.241, p = 0.236)$ $screen = (r_s(27) = -0.630, p < 0.001)^*$	$VR = (r_s(26) = 0.056, p = 0.785)$ $screen = (r_s(27) = -0.301, p = 0.128)$	$VR = (r_s(26) = 0.082, p = 0.691)$ $screen = (r_s(27) = -0.224, p = 0.261)$	-	-	-	-	-
Activity c	$VR = (r_s(26) = 0.111, p = 0.590)$ $screen = (r_s(27) = 0.559, p = 0.002)^*$	$VR = (r_s(26) = -0.267, p = 0.188)$ $screen = (r_s(27) = -0.285, p = 0.150)$	$VR = (r_s(26) = -0.210, p = 0.304)$ $screen = (r_s(27) = -0.247, p = 0.214)$	$VR = (r_s(26) = -0.622, p = 0.001)^*$ $screen = (r_s(27) = -0.523, p = 0.005)^*$	$VR = (r_s(26) = -0.207, p = 0.310)$ $screen = (r_s(27) = 0.041, p = 0.837)$	-	-	-	-
Potency T	$VR = (r_s(26) = 0.339, p = 0.090)$ $screen = (r_s(27) = -0.246, p = 0.216)$	$VR = (r_s(26) = -0.236, p = 0.247)$ $screen = (r_s(27) = 0.141, p = 0.485)$	$VR = (r_s(26) = -0.157, p = 0.444)$ $screen = (r_s(27) = 0.337, p = 0.086)$	$VR = (r_s(26) = -0.090, p = 0.661)$ $screen = (r_s(27) = 0.286, p = 0.147)$	$VR = (r_s(26) = 0.236, p = 0.246)$ $screen = (r_s(27) = 0.140, p = 0.486)$	$VR = (r_s(26) = -0.081, p = 0.695)$ $screen = (r_s(27) = -0.272, p = 0.169)$	-	-	-
Potency t	$VR = (r_s(26) = -0.166, p = 0.417)$ $screen = (r_s(27) = 0.099, p = 0.622)$	$VR = (r_s(26) = 0.187, p = 0.360)$ $screen = (r_s(27) = 0.008, p = 0.970)$	$VR = (r_s(26) = 0.152, p = 0.459)$ $screen = (r_s(27) = -0.022, p = 0.911)$	$VR = (r_s(26) = 0.382, p = 0.054)$ $screen = (r_s(27) = 0.077, p = 0.702)$	$VR = (r_s(26) = 0.575, p = 0.002)^*$ $screen = (r_s(27) = 0.491, p = 0.009)^*$	$VR = (r_s(26) = -0.405, p = 0.040)^*$ $screen = (r_s(27) = 0.035, p = 0.864)$	$VR = (r_s(26) = -0.076, p = 0.711)$ $screen = (r_s(27) = 0.015, p = 0.942)$	-	-
Potency c	$VR = (r_s(26) = -0.056, p = 0.785)$ $screen = (r_s(27) = 0.196, p = 0.328)$	$VR = (r_s(26) = -0.045, p = 0.828)$ $screen = (r_s(27) = -0.316, p = 0.109)$	$VR = (r_s(26) = 0.039, p = 0.850)$ $screen = (r_s(27) = -0.013, p = 0.948)$	$VR = (r_s(26) = 0.079, p = 0.703)$ $screen = (r_s(27) = -0.025, p = 0.901)$	$VR = (r_s(26) = -0.176, p = 0.389)$ $screen = (r_s(27) = -0.016, p = 0.937)$	$VR = (r_s(26) = 0.341, p = 0.088)$ $screen = (r_s(27) = 0.379, p = 0.052)$	$VR = (r_s(26) = -0.469, p = 0.016)^*$ $screen = (r_s(27) = -0.354, p = 0.070)$	$VR = (r_s(26) = 0.113, p = 0.582)$ $screen = (r_s(27) = -0.087, p = 0.666)$	-

Table S6.22: Spearman correlations between Greenberg-Strickland variables. Asterisks (*) indicate statistically significant differences ($p < 0.05$). Please see the main text for a discussion of the observed effects.

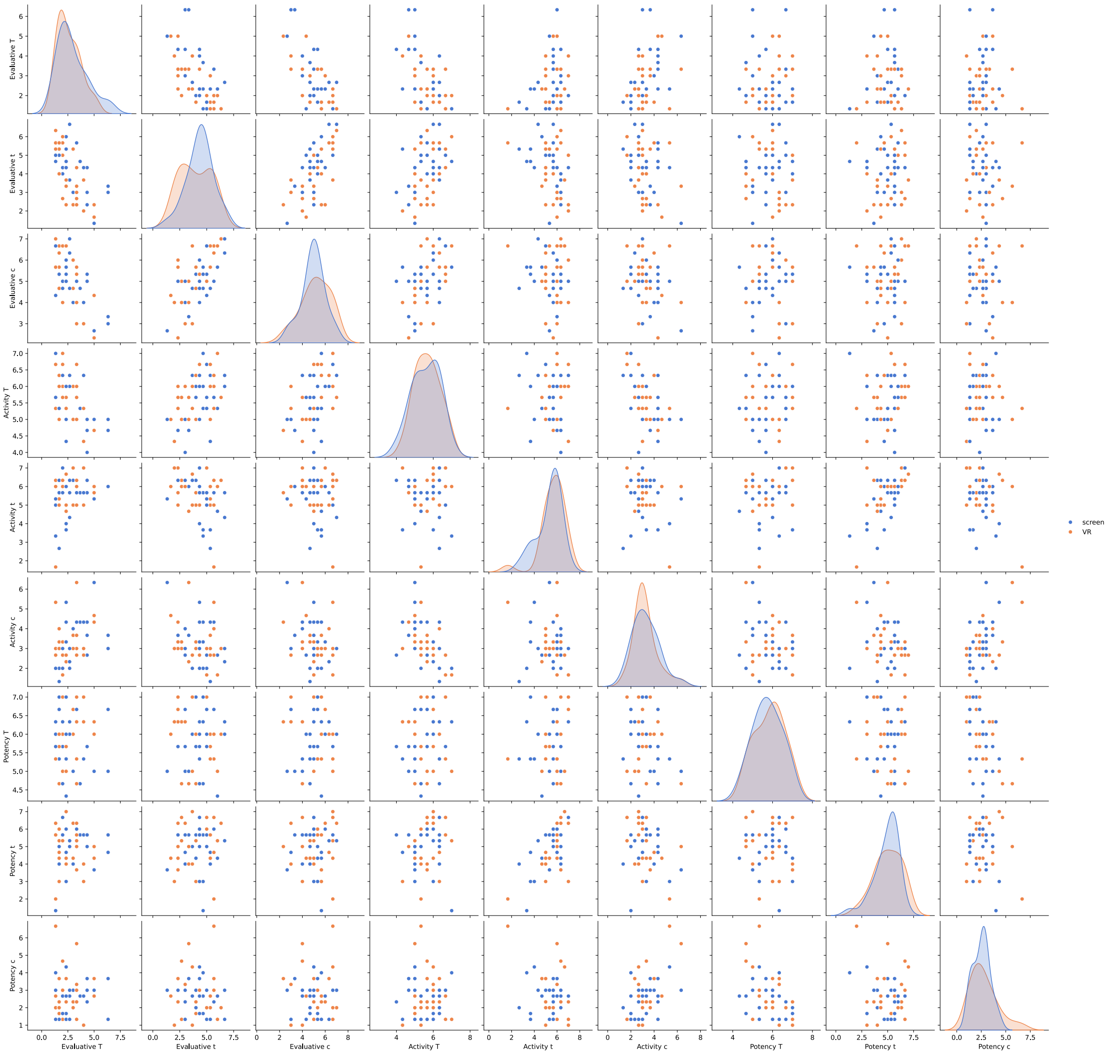

Figure S6.16: Pairwise relationships among Greenberg-Strickland variables. The diagonal plots represent univariate distributions, showing the marginal distribution of the data in each column.

	Evaluative T	Evaluative t	Evaluative c	Activity T	Activity t	Activity c	Potency T	Potency t	Potency c
No. of propositions 1st	$VR = (r_s(26) = 0.109, p = 0.597)$<br>$screen = (r_s(27) = 0.282, p = 0.153)$	$VR = (r_s(26) = -0.13, p = 0.528)$<br>$screen = (r_s(27) = -0.052, p = 0.798)$	$VR = (r_s(26) = 0.017, p = 0.933)$<br>$screen = (r_s(27) = -0.121, p = 0.548)$	$VR = (r_s(26) = -0.101, p = 0.623)$<br>$screen = (r_s(27) = 0.113, p = 0.576)$	$VR = (r_s(26) = 0.125, p = 0.542)$<br>$screen = (r_s(27) = 0.157, p = 0.435)$	$VR = (r_s(26) = 0.127, p = 0.536)$<br>$screen = (r_s(27) = 0.11, p = 0.586)$	$VR = (r_s(26) = -0.158, p = 0.442)$<br>$screen = (r_s(27) = -0.001, p = 0.995)$	$VR = (r_s(26) = 0.022, p = 0.914)$<br>$screen = (r_s(27) = 0.187, p = 0.351)$	$VR = (r_s(26) = -0.002, p = 0.993)$<br>$screen = (r_s(27) = 0.078, p = 0.697)$
No. of propositions 2nd	$VR = (r_s(26) = -0.152, p = 0.458)$<br>$screen = (r_s(26) = 0.344, p = 0.085)$	$VR = (r_s(26) = 0.011, p = 0.957)$<br>$screen = (r_s(26) = -0.002, p = 0.991)$	$VR = (r_s(26) = -0.003, p = 0.988)$<br>$screen = (r_s(26) = -0.001, p = 0.995)$	$VR = (r_s(26) = 0.19, p = 0.354)$<br>$screen = (r_s(26) = -0.094, p = 0.646)$	$VR = (r_s(26) = 0.136, p = 0.508)$<br>$screen = (r_s(26) = 0.195, p = 0.34)$	$VR = (r_s(26) = -0.258, p = 0.203)$<br>$screen = (r_s(26) = 0.128, p = 0.532)$	$VR = (r_s(26) = -0.071, p = 0.73)$<br>$screen = (r_s(26) = 0.203, p = 0.319)$	$VR = (r_s(26) = 0.246, p = 0.225)$<br>$screen = (r_s(26) = 0.249, p = 0.221)$	$VR = (r_s(26) = -0.024, p = 0.908)$<br>$screen = (r_s(26) = -0.048, p = 0.815)$
Pertinence Index 1st	$VR = (r_s(26) = -0.128, p = 0.534)$<br>$screen = (r_s(27) = 0.028, p = 0.889)$	$VR = (r_s(26) = 0.37, p = 0.063)$<br>$screen = (r_s(27) = 0.061, p = 0.764)$	$VR = (r_s(26) = 0.26, p = 0.2)$<br>$screen = (r_s(27) = -0.206, p = 0.303)$	$VR = (r_s(26) = 0.055, p = 0.791)$<br>$screen = (r_s(27) = 0.014, p = 0.943)$	$VR = (r_s(26) = -0.144, p = 0.483)$<br>$screen = (r_s(27) = -0.271, p = 0.172)$	$VR = (r_s(26) = -0.059, p = 0.774)$<br>$screen = (r_s(27) = 0.264, p = 0.184)$	$VR = (r_s(26) = -0.289, p = 0.152)$<br>$screen = (r_s(27) = -0.206, p = 0.302)$	$VR = (r_s(26) = -0.15, p = 0.465)$<br>$screen = (r_s(27) = -0.029, p = 0.886)$	$VR = (r_s(26) = 0.253, p = 0.213)$<br>$screen = (r_s(27) = 0.261, p = 0.188)$
Pertinence Index 2nd	$VR = (r_s(26) = -0.188, p = 0.358)$<br>$screen = (r_s(26) = -0.272, p = 0.18)$	$VR = (r_s(26) = 0.055, p = 0.79)$<br>$screen = (r_s(26) = 0.235, p = 0.247)$	$VR = (r_s(26) = 0.131, p = 0.523)$<br>$screen = (r_s(26) = 0.246, p = 0.226)$	$VR = (r_s(26) = 0.239, p = 0.24)$<br>$screen = (r_s(26) = 0.397, p = 0.045)^*$	$VR = (r_s(26) = 0.102, p = 0.619)$<br>$screen = (r_s(26) = -0.05, p = 0.809)$	$VR = (r_s(26) = -0.037, p = 0.857)$<br>$screen = (r_s(26) = -0.217, p = 0.288)$	$VR = (r_s(26) = -0.592, p = 0.001)^*$<br>$screen = (r_s(26) = 0.127, p = 0.537)$	$VR = (r_s(26) = 0.202, p = 0.323)$<br>$screen = (r_s(26) = 0.119, p = 0.562)$	$VR = (r_s(26) = 0.471, p = 0.015)^*$<br>$screen = (r_s(26) = 0.14, p = 0.494)$
Salience Index 1st	$VR = (r_s(26) = 0.083, p = 0.687)$<br>$screen = (r_s(27) = 0.222, p = 0.266)$	$VR = (r_s(26) = -0.277, p = 0.171)$<br>$screen = (r_s(27) = -0.25, p = 0.209)$	$VR = (r_s(26) = -0.13, p = 0.527)$<br>$screen = (r_s(27) = -0.14, p = 0.487)$	$VR = (r_s(26) = -0.373, p = 0.061)$<br>$screen = (r_s(27) = 0.024, p = 0.905)$	$VR = (r_s(26) = 0.344, p = 0.086)$<br>$screen = (r_s(27) = 0.429, p = 0.026)^*$	$VR = (r_s(26) = 0.3, p = 0.137)$<br>$screen = (r_s(27) = -0.062, p = 0.757)$	$VR = (r_s(26) = 0.086, p = 0.677)$<br>$screen = (r_s(27) = 0.052, p = 0.795)$	$VR = (r_s(26) = 0.16, p = 0.434)$<br>$screen = (r_s(27) = 0.149, p = 0.459)$	$VR = (r_s(26) = -0.291, p = 0.15)$<br>$screen = (r_s(27) = -0.172, p = 0.391)$
Salience Index 2nd	$VR = (r_s(26) = -0.151, p = 0.46)$<br>$screen = (r_s(26) = 0.267, p = 0.187)$	$VR = (r_s(26) = 0.209, p = 0.305)$<br>$screen = (r_s(26) = -0.047, p = 0.82)$	$VR = (r_s(26) = 0.126, p = 0.541)$<br>$screen = (r_s(26) = -0.462, p = 0.018)^*$	$VR = (r_s(26) = 0.11, p = 0.592)$<br>$screen = (r_s(26) = -0.084, p = 0.685)$	$VR = (r_s(26) = -0.145, p = 0.479)$<br>$screen = (r_s(26) = -0.058, p = 0.78)$	$VR = (r_s(26) = -0.271, p = 0.181)$<br>$screen = (r_s(26) = 0.125, p = 0.542)$	$VR = (r_s(26) = 0.118, p = 0.567)$<br>$screen = (r_s(26) = -0.032, p = 0.877)$	$VR = (r_s(26) = -0.036, p = 0.863)$<br>$screen = (r_s(26) = 0.031, p = 0.879)$	$VR = (r_s(26) = -0.391, p = 0.049)^*$<br>$screen = (r_s(26) = -0.274, p = 0.175)$
Cognition Index 1st	$VR = (r_s(26) = 0.053, p = 0.796)$<br>$screen = (r_s(27) = 0.415, p = 0.031)^*$	$VR = (r_s(26) = 0.222, p = 0.277)$<br>$screen = (r_s(27) = -0.204, p = 0.307)$	$VR = (r_s(26) = 0.05, p = 0.808)$<br>$screen = (r_s(27) = -0.128, p = 0.524)$	$VR = (r_s(26) = -0.055, p = 0.789)$<br>$screen = (r_s(27) = -0.213, p = 0.287)$	$VR = (r_s(26) = -0.377, p = 0.058)$<br>$screen = (r_s(27) = 0.192, p = 0.337)$	$VR = (r_s(26) = 0.005, p = 0.981)$<br>$screen = (r_s(27) = 0.193, p = 0.334)$	$VR = (r_s(26) = 0.115, p = 0.577)$<br>$screen = (r_s(27) = -0.261, p = 0.189)$	$VR = (r_s(26) = -0.284, p = 0.159)$<br>$screen = (r_s(27) = 0.234, p = 0.239)$	$VR = (r_s(26) = -0.125, p = 0.542)$<br>$screen = (r_s(27) = 0.037, p = 0.855)$
Cognition Index 2nd	$VR = (r_s(26) = 0.317, p = 0.115)$<br>$screen = (r_s(26) = 0.079, p = 0.701)$	$VR = (r_s(26) = -0.49, p = 0.011)^*$<br>$screen = (r_s(26) = 0.03, p = 0.885)$	$VR = (r_s(26) = -0.261, p = 0.197)$<br>$screen = (r_s(26) = 0.22, p = 0.279)$	$VR = (r_s(26) = -0.154, p = 0.451)$<br>$screen = (r_s(26) = -0.036, p = 0.863)$	$VR = (r_s(26) = 0.189, p = 0.354)$<br>$screen = (r_s(26) = 0.041, p = 0.841)$	$VR = (r_s(26) = -0.045, p = 0.829)$<br>$screen = (r_s(26) = 0.111, p = 0.59)$	$VR = (r_s(26) = 0.034, p = 0.869)$<br>$screen = (r_s(26) = 0.085, p = 0.681)$	$VR = (r_s(26) = 0.036, p = 0.861)$<br>$screen = (r_s(26) = 0.115, p = 0.576)$	$VR = (r_s(26) = -0.111, p = 0.59)$<br>$screen = (r_s(26) = 0.01, p = 0.962)$
Affective Index 1st	$VR = (r_s(26) = -0.035, p = 0.866)$<br>$screen = (r_s(27) = -0.44, p = 0.022)^*$	$VR = (r_s(26) = -0.272, p = 0.179)$<br>$screen = (r_s(27) = 0.223, p = 0.263)$	$VR = (r_s(26) = -0.089, p = 0.667)$<br>$screen = (r_s(27) = 0.142, p = 0.479)$	$VR = (r_s(26) = 0.047, p = 0.819)$<br>$screen = (r_s(27) = 0.222, p = 0.265)$	$VR = (r_s(26) = 0.436, p = 0.026)^*$<br>$screen = (r_s(27) = -0.218, p = 0.274)$	$VR = (r_s(26) = 0.009, p = 0.965)$<br>$screen = (r_s(27) = -0.202, p = 0.312)$	$VR = (r_s(26) = -0.091, p = 0.658)$<br>$screen = (r_s(27) = 0.286, p = 0.147)$	$VR = (r_s(26) = 0.326, p = 0.104)$<br>$screen = (r_s(27) = -0.25, p = 0.208)$	$VR = (r_s(26) = 0.118, p = 0.564)$<br>$screen = (r_s(27) = -0.064, p = 0.751)$
Affective Index 2nd	$VR = (r_s(26) = -0.199, p = 0.329)$<br>$screen = (r_s(26) = -0.089, p = 0.664)$	$VR = (r_s(26) = 0.438, p = 0.025)^*$<br>$screen = (r_s(26) = -0.038, p = 0.854)$	$VR = (r_s(26) = 0.137, p = 0.504)$<br>$screen = (r_s(26) = -0.193, p = 0.345)$	$VR = (r_s(26) = 0.039, p = 0.848)$<br>$screen = (r_s(26) = 0.031, p = 0.88)$	$VR = (r_s(26) = -0.137, p = 0.505)$<br>$screen = (r_s(26) = -0.099, p = 0.63)$	$VR = (r_s(26) = 0.066, p = 0.748)$<br>$screen = (r_s(26) = -0.02, p = 0.921)$	$VR = (r_s(26) = 0.058, p = 0.777)$<br>$screen = (r_s(26) = -0.139, p = 0.497)$	$VR = (r_s(26) = -0.064, p = 0.755)$<br>$screen = (r_s(26) = -0.148, p = 0.471)$	$VR = (r_s(26) = 0.058, p = 0.777)$<br>$screen = (r_s(26) = 0.037, p = 0.856)$
Animation Index 1st	$VR = (r_s(26) = 0.334, p = 0.096)$<br>$screen = (r_s(27) = -0.234, p = 0.24)$	$VR = (r_s(26) = -0.53, p = 0.005)^*$<br>$screen = (r_s(27) = 0.181, p = 0.367)$	$VR = (r_s(26) = -0.41, p = 0.037)^*$<br>$screen = (r_s(27) = 0.365, p = 0.061)$	$VR = (r_s(26) = -0.342, p = 0.088)$<br>$screen = (r_s(27) = 0.235, p = 0.238)$	$VR = (r_s(26) = 0.466, p = 0.017)^*$<br>$screen = (r_s(27) = 0.134, p = 0.504)$	$VR = (r_s(26) = 0.157, p = 0.442)$<br>$screen = (r_s(27) = -0.226, p = 0.258)$	$VR = (r_s(26) = 0.29, p = 0.151)$<br>$screen = (r_s(27) = 0.431, p = 0.025)^*$	$VR = (r_s(26) = 0.134, p = 0.513)$<br>$screen = (r_s(27) = 0.117, p = 0.561)$	$VR = (r_s(26) = -0.299, p = 0.138)$<br>$screen = (r_s(27) = -0.174, p = 0.387)$
Animation Index 2nd	$VR = (r_s(26) = -0.072, p = 0.728)$<br>$screen = (r_s(26) = 0.473, p = 0.015)^*$	$VR = (r_s(26) = 0.174, p = 0.396)$<br>$screen = (r_s(26) = -0.079, p = 0.703)$	$VR = (r_s(26) = 0.034, p = 0.87)$<br>$screen = (r_s(26) = -0.173, p = 0.399)$	$VR = (r_s(26) = 0.115, p = 0.575)$<br>$screen = (r_s(26) = -0.368, p = 0.064)$	$VR = (r_s(26) = -0.331, p = 0.099)$<br>$screen = (r_s(26) = 0.216, p = 0.288)$	$VR = (r_s(26) = -0.221, p = 0.279)$<br>$screen = (r_s(26) = 0.201, p = 0.325)$	$VR = (r_s(26) = 0.154, p = 0.451)$<br>$screen = (r_s(26) = -0.153, p = 0.457)$	$VR = (r_s(26) = -0.166, p = 0.417)$<br>$screen = (r_s(26) = -0.112, p = 0.585)$	$VR = (r_s(26) = -0.174, p = 0.394)$<br>$screen = (r_s(26) = -0.154, p = 0.453)$
Person Index	$VR = (r_s(26) = -0.16, p = 0.436)$<br>$screen = (r_s(27) = -0.324, p = 0.099)$	$VR = (r_s(26) = -0.229, p = 0.26)$<br>$screen = (r_s(27) = -0.071, p = 0.725)$	$VR = (r_s(26) = -0.007, p = 0.974)$<br>$screen = (r_s(27) = 0.109, p = 0.589)$	$VR = (r_s(26) = 0.036, p = 0.86)$<br>$screen = (r_s(27) = 0.078, p = 0.698)$	$VR = (r_s(26) = 0.383, p = 0.053)$<br>$screen = (r_s(27) = 0.187, p = 0.351)$	$VR = (r_s(26) = -0.208, p = 0.307)$<br>$screen = (r_s(27) = -0.308, p = 0.118)$	$VR = (r_s(26) = 0.195, p = 0.341)$<br>$screen = (r_s(27) = 0.003, p = 0.99)$	$VR = (r_s(26) = 0.15, p = 0.466)$<br>$screen = (r_s(27) = -0.198, p = 0.323)$	$VR = (r_s(26) = -0.254, p = 0.211)$<br>$screen = (r_s(27) = -0.019, p = 0.924)$
Problem-Solving Index	$VR = (r_s(26) = -0.024, p = 0.906)$<br>$screen = (r_s(27) = -0.32, p = 0.104)$	$VR = (r_s(26) = 0.119, p = 0.563)$<br>$screen = (r_s(27) = 0.094, p = 0.639)$	$VR = (r_s(26) = 0.097, p = 0.638)$<br>$screen = (r_s(27) = 0.097, p = 0.629)$	$VR = (r_s(26) = 0.138, p = 0.502)$<br>$screen = (r_s(27) = 0.113, p = 0.575)$	$VR = (r_s(26) = 0.109, p = 0.595)$<br>$screen = (r_s(27) = -0.093, p = 0.644)$	$VR = (r_s(26) = -0.409, p = 0.038)^*$<br>$screen = (r_s(27) = -0.255, p = 0.2)$	$VR = (r_s(26) = 0.293, p = 0.146)$<br>$screen = (r_s(27) = -0.025, p = 0.903)$	$VR = (r_s(26) = 0.241, p = 0.237)$<br>$screen = (r_s(27) = -0.115, p = 0.57)$	$VR = (r_s(26) = -0.39, p = 0.049)^*$<br>$screen = (r_s(27) = 0.021, p = 0.917)$

Table S6.23: Spearman correlations between Greenberg-Strickland variables and the SAT indices. Asterisks (*) indicate statistically significant differences ($p < 0.05$). Please see the main text for a discussion of the observed effects.

S6.6 Presence statistical analyses

In this subsection, full statistical table of the analyses performed in the main document are reported. Results are shown in Table S6.24.

Presence metric	Test		VR vs. screen
Exciting	Mann-Whitney U	U	265.500
		p	0.114
		$p_{adjusted}$	0.211
Being there	Mann-Whitney U	U	338.500
		p	0.820
		$p_{adjusted}$	0.820
Present	Mann-Whitney U	U	272.500
		p	0.211
		$p_{adjusted}$	0.211
Focused	Mann-Whitney U	U	244.500
		p	0.039*
		$p_{adjusted}$	0.160

Table S6.24: Presence questionnaire statistical analyses. We also report the adjusted p-value using the Benjamini-Hochberg method. Asterisks (*) indicate indicate statistically significant differences ($p < 0.05$).

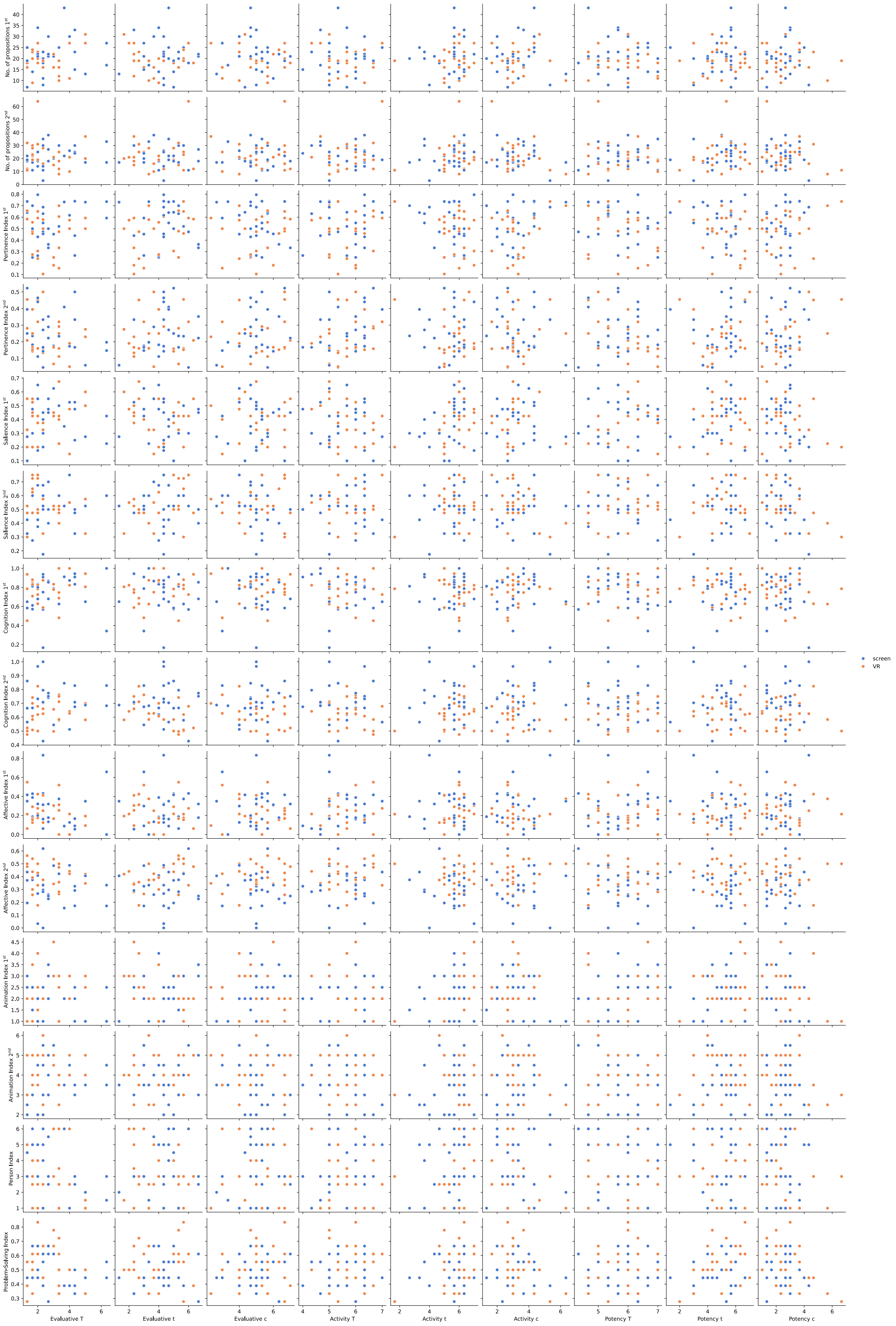

Figure S6.17: Pairwise relationships among Greenberg-Strickland variables and the SAT indices.

References

[1] Cicchetti, D. V. (1994). Guidelines, criteria, and rules of thumb for evaluating normed and standardized assessment instruments in psychology. *Psychological assessment*, 6(4):284.

[2] Heider, F. and Simmel, M. (1944). An experimental study of apparent behavior. *The American journal of psychology*, 57(2):243–259.

[3] Klin, A. (2000). Attributing social meaning to ambiguous visual stimuli in higher-functioning autism and asperger syndrome: The social attribution task. *The Journal of Child Psychology and Psychiatry and Allied Disciplines*, 41(7):831–846.

[4] Koo, T. K. and Li, M. Y. (2016). A guideline of selecting and reporting intraclass correlation coefficients for reliability research. *Journal of chiropractic medicine*, 15(2):155–163.

[5] Ratajska, A., Brown, M. I., and Chabris, C. F. (2020). Attributing social meaning to animated shapes: A new experimental study of apparent behavior. *The American Journal of Psychology*, 133(3):295–312.

[6] Shrout, P. E. and Fleiss, J. L. (1979). Intraclass correlations: uses in assessing rater reliability. *Psychological bulletin*, 86(2):420.
